# Over-optimization of academic publishing metrics: observing Goodhart’s Law in action

**DOI:** 10.1093/gigascience/giz053

**Published:** 2019-05-30

**Authors:** Michael Fire, Carlos Guestrin

**Affiliations:** 1 Software and Information Systems Engineering Department, Ben-Gurion University, Be'er Sheva 84105, Israel; 2 Paul G. Allen School of Computer Science & Engineering, University of Washington, Stevens Way NE, Seattle, WA 98195, USA

**Keywords:** science of science, scientometrics, Goodhart’s Law, data science, big data, academic publishing metrics

## Abstract

**Background:**

The academic publishing world is changing significantly, with ever-growing numbers of publications each year and shifting publishing patterns. However, the metrics used to measure academic success, such as the number of publications, citation number, and impact factor, have not changed for decades. Moreover, recent studies indicate that these metrics have become targets and follow Goodhart’s Law, according to which, “when a measure becomes a target, it ceases to be a good measure.”

**Results:**

In this study, we analyzed >120 million papers to examine how the academic publishing world has evolved over the last century, with a deeper look into the specific field of biology. Our study shows that the validity of citation-based measures is being compromised and their usefulness is lessening. In particular, the number of publications has ceased to be a good metric as a result of longer author lists, shorter papers, and surging publication numbers. Citation-based metrics, such citation number and h-index, are likewise affected by the flood of papers, self-citations, and lengthy reference lists. Measures such as a journal’s impact factor have also ceased to be good metrics due to the soaring numbers of papers that are published in top journals, particularly from the same pool of authors. Moreover, by analyzing properties of >2,600 research fields, we observed that citation-based metrics are not beneficial for comparing researchers in different fields, or even in the same department.

**Conclusions:**

Academic publishing has changed considerably; now we need to reconsider how we measure success.

## Introduction

In the past century, the academic publishing world has changed drastically in volume and velocity [1]. The volume of papers has increased sharply from <1 million papers published in 1980 to >7 million papers published in 2014 [2]. Furthermore, the speed at which researchers can share and publish their studies has increased significantly. Today’s researchers can publish not only in an ever-growing number of traditional venues, such as conferences and journals, but also in electronic preprint repositories and in mega-journals that provide rapid publication times [1, 3].

Along with the exponential increase in the quantity of published papers, the number of ranked scientific journals has increased to >34,000 active peer-reviewed journals in 2014 [1], and the number of published researchers has soared [4]. As part of this escalation, metrics such as the number of papers, number of citations, impact factor, h-index, and altmetrics are being used to compare the impact of papers, researchers, journals, and universities [5–8]. Using quantitative metrics to rank researchers contributes to a hypercompetitive research environment, which is changing academic culture—and not in a positive direction [9].

Studies suggest that publication patterns have changed as a result of Goodhart’s Law, according to which, “When a measure becomes a target, it ceases to be a good measure” [9, 10]. Goodhart’s Law, and the closely related Campbell’s Law [11], influence many systems in our everyday life, including educational [11], biological [12], and other decision-making systems [13, 14]. As an example, Goodhart’s Law can be found operating in the New York Police Department’s manipulation of crime reports (the “measure”) in order to improve crime statistics (the “target”) [15]. Another example is found in the educational system, revealing that when “test scores become the goal of the teaching process, they both lose their value as indicators of educational status and distort the educational process in undesirable ways” [11]. One more example can be found in the field of medicine, where the National Health Service in the UK sets incentives (pay for performance) for primary care physicians to improve the quality of care. Indeed, “they found the measures improved for diabetes and asthma care in the first years of the program. These improvements were on the basis of care reported in the medical records but not necessarily on care provided. The main effect of this pay-for-performance program may be to promote better recording of care rather than better care.” [16]

Recent studies indicate that when measures become targets in academic publishing, the effectiveness of the measures can be compromised, and unwelcome and unethical behaviors may develop, such as salami publications [17], ghost authorships [18], p-hacking [19], metrics manipulation [20], faking research data [21], faking of peer reviews [22], and even plagiarizing by a peer reviewer [23].

If the influence of Goodhart’s Law on academia is indeed significant, then it should be possible to observe that academic entities, such as researchers and journals, will over-optimize their own measures to achieve a desired target. Similar to the consequences of making test scores a target, chasing after certain measures in the academic publishing world to gain advantage in the battle of “impact or perish” [10] can have undesirable effects.

Certainly, newer academic publishing metrics have emerged that are less biased [20, 24–27], and these may thwart the trend of measures becoming targets. Yet, the traditional metrics retain a strong hold on the overall academic system, and they are still widely used for ranking purposes [28, 29].

In the present study, our main goal was to utilize new advances in data science tools to perform an in-depth and precise bottom-up analysis of academic publishing over the decades. Our comprehensive analysis ranged from micro to macro levels as we studied individual researchers’ behaviors as well as behavioral changes within large research domains. Additionally, we wanted to uncover how and whether Goodhart’s Law has changed academic publishing, with an in-depth look at trends within biology and genetics.

Our study was greatly influenced by a recent study by Edwards and Roy [9], who observed that academia has become a hypercompetitive environment that can lead to unethical behaviors. The driving force behind such behaviors is an effort to manipulate the metrics that measure the research’s impact solely to increase the quantitative measures (and hence the status) of the research.

To achieve our research goals, we developed an open source code framework to analyze data from several large-scale datasets containing >120 million publications, with 528 million references and 35 million authors, since the beginning of the 19th century (see Results of Author Trends section). This provided a precise and full picture of how the academic publishing world has evolved.

The objective of our study was to use this huge quantity of data to examine the validity of commonly used citation-based metrics for academic publishing. Specifically, we wanted to see whether Goodhart’s Law applied: *are researchers focusing too much on simply attaining certain target metrics at the expense of high-quality, relevant research?*

The remainder of the paper is organized as follows: in the Background section, we provide an overview of related studies. In the Data Description section, we present the datasets used in this study, and in the Analyses section, we describe the algorithms and experiments used to analyze the study’s data. In the Results, Discussion, and Conclusions sections, we present and discuss our results and offer our conclusions from the present study.

## Background

This research is a large-scale scientometrics study (also referred to as the “science of science” [30]). Scientometrics is the study of quantitative features and characteristics of scientific research. In this section, we present studies that analyze changes in academic publications in recent years (see the Changes in Publication Trends section), and we provide an overview of common metrics that measure the impact of published papers (see the Success Metrics and Citation Trends section).

### Changes in publication trends

One prevalent and increasing trend is to publish papers in preprint repositories, such as arXiv, bioRxiv, and Research Papers in Economics (RePEc) [1]. For example, the use of arXiv surged from 4,275 submitted papers in September 2006 to 11,973 papers in November 2018 [31]. Additionally, >1 million papers are now downloaded from bioRxiv every month [32]. Another current trend is to publish papers in mega-journals, such as *PLoS One* and *Nature’s Scientific Reports*. Mega-journals are a new type of scientific journal that publishes peer-reviewed, open-access articles, where the articles have been reviewed for scientific trustworthiness but not for scientific merit. Mega-journals accelerate review and publication times to 3−5 months and usually have high acceptance rates of >50% [3]. In the first quarter of 2017, >11,000 papers were published in *PLoS One* and *Scientific Reports* [33].

Another observable trend is that more and more papers are written by hundreds or even thousands of authors. This phenomenon is known as hyperauthorship [34] or author inflation [35] and is common across research fields, where the majority of papers with >1,000 authors are produced in the physical sciences [36]. For example, the recent Laser Interferometer Gravitational-Wave Observatory paper [37] listed >1,000 authors [38]. Robert Aboukhalil measured this trend [39] and discovered that the mean number of authors of academic papers has increased sharply since the beginning of the 20th century. Recently, Steven Kelly observed an unexpected increase in the mean number of authors of papers in the biological sciences [4].

While papers’ mean number of authors has increased over time, not all the authors have significantly contributed to the paper. In addition, honorary and ghost authors are prevalent. Wislar et al. found such evidence in biomedical journals [40], and similar findings were observed by Kennedy et al. [41] and by Vera-Badillo et al. [42]. *The Economist* recently published an article titled “Why research papers have so many authors” [43].

Lewison and Hartley [44] analyzed how papers’ titles have changed over time. They discovered that titles’ lengths have been increasing, along with the percentage of titles containing colons. Additionally, Gwilym Lockwood observed that “articles with positively-framed titles, interesting phrasing, and no wordplay get more attention online” [45].

In addition to paper title lengths increasing, Ucar et al. have found lengthening reference lists for engineering journal articles, such as those published in *Biomedical Engineering and Information Theory* [46].

Additionally, many studies have focused on how publication trends have changed over time, often focusing on specific geographical areas, demographic characteristics, specific research domains, or specific journals. For example, Gálvez et al. [47] used the Science Citation Index to understand publication patterns in the developing world. Jagsi et al. [48] studied the gender gap in authorship of academic medical literature over 35 years. They discovered that the percentage of first and last authors who were women increased from 5.9% and 3.7% in 1970 to 29.3% and 19.3%, respectively, in 2004. Johnson et al. [49] studied publication trends in top-tier journals of higher education. Peter Aldhous analyzed publications in the National Academy of Sciences (*PNAS* [*Proceedings of the National Academyof Sciences of the United Statesof America*]) journal to consider the influence of an “old boys’ club” mentality [50]. In 2009, Porter and Rafols [51] used bibliometric indicators alongside a new index of interdisciplinarity to measure how the degree of interdisciplinarity has changed between 1975 and 2005 for 6 research domains. Porter and Rafols’ findings suggest that “science is indeed becoming more interdisciplinary, but in small steps.”

In 2016, Fanelli and Larivière [52] analyzed the publication patterns of >40,000 researchers for more than a century. They observed that for researchers in their early career, both the total number of papers and the mean number of collaborators increased over time. Fanelli and Larivière also observed that when the publication rate was adjusted to account for co-authorship, then “the publication rate of scientists in all disciplines has not increased overall, and has actually mostly declined” [52]. In 2017, Dong et al. [53] used a dataset consisting of 89 million publications to study the evolution of scientific development over the past century. In their study, Dong et al. examined trends in collaborations, citations, and impact. From the collaboration perspective, Dong et al. observed that “the average length of a publication’s author list tripled between 1900 and 2015, suggesting an increasingly collaborative scientific process.” From analyzing citation patterns, they observed a sharp increase in the number of references over time, where in recent years, on average, papers reference 30 other papers. From the perspective of impact and innovations, Dong et al. observed “diversification of scientific development across the planet over the past century” [53]. While both our study and that of Dong et al. use the Microsoft Academic Graph (MAG) dataset, Dong et al. focused on the advancement of science and the globalization of scientific collaborations, citations, and innovations. Our study’s primary goal was to perform an in-depth analysis of how the world of academic publishing has evolved over the decades. Moreover, we used additional large-scale datasets (see the Data Description section) to fully examine how academic publishing has evolved, investigating both micro trends (trends in the structure of papers) and macro trends (trends within research fields).

### Success metrics and citation trends

Over the years, various metrics have been proposed to measure papers, journal importance, and authors’ impact. One of the most straightforward and commonly used measures is to simply count the researcher’s number of publications. Another common metric is the citation number, either of a particular paper or the total citations received by all the author’s papers. However, not all citations are equal [54]. Moreover, different research fields have different citation metrics, and therefore comparing them creates a problem: “The purpose of comparing citation records is to discriminate between scientists” [55].

One of the best-known and most-used measures to evaluate journals’ importance is the impact factor, devised >60 years ago by Eugene Garfield [7]. The impact factor measures the frequency in which an average article in a journal has been cited in a specific year. Over time, the measure has been used to “evaluate institutions, scientific research, entire journals, and individual articles” [56]. Another common metric to measure a researcher’s output or a journal’s impact is the h-index, which measures an author’s or a journal’s number of papers that have at least *h* citations each [6]. It has been shown that the h-index can predict academic achievements [57].

The above measures have been the standard for measuring academic publishing success. According to recent studies, and following Goodhart’s Law, these metrics have now become targets, ripe for manipulation [9, 10, 58]. All types of manipulative methods are used, such as increasing the number of self-citations [20], increasing the number of publications by slicing studies into the smallest quantum acceptable for publication [59], indexing false papers [60], and merging papers on Google Scholar [61]. Indeed, a recent study by Fong and Wilhite [58], which used data from >12,000 responses to a series of surveys sent to >110,000 scholars from 18 different disciplines, discovered “widespread misattribution in publications and in research proposals.” Fong and Wilhite’s findings revealed that the majority of researchers disapprove of this type of metric manipulation, yet many feel pressured to participate; other researchers blandly state “that it is just the way the game is played” [58].

While many of the aforementioned measures are easy to compute, they fail to consider the added contribution that is generally provided by the first and last authors. This issue becomes more cardinal with a sharply increasing number of papers with hundreds of co-authors. For example, “the h-index does not work well in the field of life sciences, where an author’s position on a paper typically depends on the author’s contribution” [25]. To tackle this issue, various measures such as the c-index [24] and revised h-index [25] have been suggested. These measures give higher weights to authors according to the co-author order.

To overcome other shortcomings of commonly used measures, other alternative measures have been suggested. For example, the q-index [20] and w-index [26] are alternatives to the h-index. Likewise, the SCImago Journal Rank (SJR indicator) [62] and simple citation distributions [63] are offered as alternatives to the impact factor. Additional measures that normalize citation-based indicators using a paper’s field of study and year of publication have also been suggested, and these are being used by several institutions [27].

Senior employees at several leading science publishers called upon journals to refrain from using the impact factor and suggested replacing it with simple citation distributions [63, 64]. Similarly, the altmetric [65] was proposed as an alternative metric to the impact factor and h-index. The altmetric [66] is a generalization of article-level metrics and considers other aspects of the impact of the work, such as the number of downloads, article views, mentions in social media, and more. The altmetric measure has gained in popularity in recent years, and several large publishers have started providing this metric to their readers. Additionally, Semantic Scholar [67] offers various measures to judge papers and researchers’ influence. A thorough report regarding potential uses and limitations of metrics was written by Wilsdon et al. [8]. Additionally, an overview of the changing scholarly landscape can be found in the study by Roemer and Borchardt [5].

Even with their many known shortcomings [8, 24, 55, 68–70], measures such as the impact factor, citation number, and h-index are still widely used. For example, the Journal Citation Reports publishes annual rankings based on journals’ impact factors, and it continues to be widely followed [29]. As another example, the widely used Google Scholar web search engine [71] calculates the h-index and total number of citations of researchers, as well as journals’ h-index, to rank journals and conferences [28].

## Data Description

### The Microsoft Academic Graph dataset

In this study we primarily used the MAG [72], which was released as part of the 2016 KDD Cup [73]. The large-scale MAG dataset contains scientific publication records of >120 million papers, along with citation relationships among those publications as well as relationships among authors, institutions, journals, conferences, and fields of study. In addition, the MAG dataset contains every author’s sequence number for each paper’s author list. Furthermore, the dataset contains links between a publication and the field or fields of study to which it belongs. The fields of study are organized in hierarchical rankings with 4 levels, L0−L3, where L0 is the highest level, such as a research field of computer science, and L3 is the lowest level, such as a research field of decision tree [2, 73]. Since its publication, the MAG dataset has gained increasing popularity among scholars who utilize the dataset for scientometric studies [74]. An in-depth overview of the MAG dataset properties was presented by Herrmannova and Knoth [2]. According to their analysis of the MAG dataset, the 5 top fields of study—based on the number of papers—are physics, computer science, engineering, chemistry, and biology, with the number of papers ranging from slightly <15 million in biology to >20 million in physics [2].

Even though the MAG dataset contains papers that were published through 2016, we wanted to use years in which the data were the most comprehensive, so we focused our analysis on 120.7 million papers that were published through the end of 2014. Furthermore, we noted that the dataset contains many papers that are news items, response letters, comments, and so forth. Even though these items are important, they can affect a correct understanding of the underlying trends in scientific publications. Therefore, we focused our research on a dataset subset, which consists of >22 million papers. This subset contains only papers that have a Digital Object Identifier (DOI) and ≥5 references. Additionally, while calculating various authors’ properties, we primarily considered only the 22.4 million authors with unique author ID values in the selected papers’ subset. (Identifying all the papers by the same author (also known as author disambiguation [75]) is a challenging task. The MAG dataset provides a unique author ID for names that were matched to be the same individual. Recently, Microsoft Academic published a post titled “How Microsoft Academic uses knowledge to address the problem of conflation/disambiguation,” which explains how Microsoft Academic performs author disambiguation [76].)

### The AMiner dataset

The AMiner open academic graph dataset [77] contains data from >154 million papers. The dataset contains various papers’ attributes, such as titles, keywords, abstracts, venues, languages, and ISSNs. In our study, we primarily used the AMiner dataset to analyze papers’ abstracts, to estimate papers’ lengths, and to compare results with those obtained using the MAG dataset in order to validate the existence of observed patterns in both datasets. The AMiner is a relatively new dataset, and we are among the first to use it for a scientometric study.

### The SCImago Journal Ranking dataset

To better understand trends in journal publications, we used the SCImago Journal Ranking (SJR) open dataset [78, 79]. This dataset contains details of >23,000 journals with unique names between 1999 and 2016. For each journal, the SJR dataset contains the journal’s SJR value, the number of published papers, the h-index, and the number of citations in each year. Additionally, the SJR dataset contains the best quartile, ranked from Q1 to Q4, of each journal. Journal quartiles are determined by the value of the boundary at the 25th, 50th, and 75th percentiles of an ordered distribution of the SJR indicator. Then, journals ranked Q1, Q2, Q3, and Q4 reflect the top 25%, 25−50%, 50−75%, and the bottom 25% of the distribution of the SJR indicator, respectively. The quartile rank is typically used to compare and rank journals within a given subject category.

### The Join dataset

To match the MAG journal IDs with their correlated various ranking measures, such as h-index and SJR, we joined all 3 datasets in the following manner: first, we joined the MAG and AMiner datasets by matching unique DOI values. Then, we matched ISSN values between the MAG-AMiner joined dataset with the SJR dataset.

## Analyses

### Analysis of publication trends

We used our developed code framework (see the Methods section) to explore how papers, authors, journals, and research fields have evolved over time. In the following subsections, we describe the specific calculations that were performed. Moreover, our Supplementary Materials section includes the precise code implementations that were used to obtain most of our results and to create the figures presented throughout the present study.

#### Paper trends

To explore how the quantity and structure of academic papers have changed over time, we performed the following: first, we calculated how many papers were published in the MAG dataset every year. Then, we utilized the pycld2 package [80] to detect the language of each paper’s title and calculated the number of papers in each language. Next, we calculated the following paper features over time:

- Mean number of words in titles and mean number of characters per word (for papers with English titles)
- Percentage of titles that used question or exclamation marks (for papers with English titles)
- Mean number of authors
- Percentage of papers in which authors appear in alphabetical order
- Mean number of words in abstracts
- Mean number of keywords
- Mean number of references
- Length of papers

In addition, we utilized the papers with existing field-of-research values, matching the papers to their corresponding fields in order to identify each paper’s top-level (L0) research field. Using the top-level data, we were able to estimate the number of multidisciplinary papers that had >1 L0 research field. Afterwards, we calculated the percentage and total number of papers with no citations after 5 years, as well as the overall percentage of papers with self-citations over time.

(we selected only papers having English titles and abstracts, existing author lists, references, and valid lengths; in addition, we checked whether the paper’s title contained question or exclamation marks) and were published between 1990 and 2009. Using the selected papers, we calculated the Spearman correlations among the title lengths, author numbers, reference numbers, overall lengths, and number of citations after 5 years. The results of the above-described calculations are presented in the Results of Paper Trends section. Moreover, the code implementation is provided in the “Part III - A: Analyzing Changing Trends in Academia - Paper Trends” Jupyter Notebook (see the Availability of Source Code and Requirements section).

#### Author trends

To study how authors’ behaviors and characteristics have changed, we performed the following: first, we calculated how the number of new authors has changed over time. Second, for all authors who published their first paper after 1950, we divided the authors into groups according to each author’s academic birth decade, i.e., the decade in which an author published his or her first paper. Next, for each group of authors with the same academic birth decade, we analyzed the following features:

- Mean number of papers the authors in each group published *n* years after they began their careers, for ∀*n* ∈ [0, 30]. We performed these group calculations taking into account all papers, as well as only papers with ≥5 references
- Mean number of conference and journal papers each group published *n* years after they began their careers, for ∀*n* ∈ [0, 30]
- Mean number of co-authors each group had *n* years after they began their careers, for ∀*n* ∈ [0, 30]
- Authors’ median sequence number each group had *n* years after they began their careers, for ∀*n* ∈ [0, 60]. Additionally, we calculated the mean percentage of times the authors in each group were first authors

The results of the above-described calculations are presented in the Results of Author Trends section. Moreover, the code implementation is provided in the “Part III - B: Analyzing Changing Trends in Academia - Author Trends” Jupyter Notebook (see the Availability of Source Code and Requirements section).

#### Journal trends

To investigate how journal publication trends have changed over time, we used the SJR dataset to calculate the following features between 1999 and 2016:

- Number of journals with unique journal IDs that were active in each year
- Number of new journals that were published each year
- Mean and maximal number of papers in each journal

Additionally, we utilized the SJR dataset to calculate how the journals’ best quartile, mean h-index, mean SJR, and mean citation number [Citation Number/Documents Number (2 years)] metrics changed between 1999 and 2016.

Furthermore, we selected the 40 journals with the highest SJR values in 2016 and matched them to their corresponding journal IDs in the MAG dataset by matching each journal’s ISSN and exact name in the MAG-AMiner joined dataset. (The top journal name was compared to the journal’s name in the MAG dataset.) Using this method, we identified 30 unique journal IDs in the MAG dataset that published 110,825 papers with ≥5 references. Then, for the matching journal IDs, we calculated the following features over time, for all papers that were published in the selected top journals:

- First and last authors’ mean career age
- Percentage of papers in which the first author had previously published in one of the top journals
- Percentage of papers in which the last author had previously published in one of the top journals

The results of the above-described calculations are presented in the Results of Journal Trends section. Moreover, the code implementation is provided in the “Part III - C: Analyzing Changing Trends in Academia - Journal Trends” Jupyter Notebook (see the Availability of Source Code and Requirements section).

Additionally, for >8,400 journals with ≥100 published papers with ≥5 references, we calculated the following features over time:

- Number of papers
- Number of authors
- Top keywords in a specific year
- First/last/all authors' mean or median academic age
- Mean length of papers
- Percentage of returning first/last/all authors, i.e., those who had published ≥1 prior paper in the journal

We developed a website with an interactive interface, which visualizes how the above features changed for each journal (see the Availability of Supporting Data and Materials section).

#### Field-of-research trends

We utilized the MAG dataset field-of-study values and the hierarchical relationship between various fields to match papers to their research fields in various levels (L0−L3). Then, for each field of study in its highest hierarchical level (L0), we calculated the following features over time: number of papers, number of authors, number of references, and mean number of citations after 5 years. Next, we focused on the field of biology, which is in the L0 level. For all the L1 subfields of biology, we repeated the same feature calculations as in the previous step. Afterwards, we focused on genetics. For all the L2 subfields of genetics, we repeated the same feature calculations as in the previous step.

Additionally, to better understand the differences in citation patterns of various fields of research, we performed the following: for each field of study with ≥100 papers published in 2009, we calculated the following features using only papers that were published in 2009 and had ≥5 references:

- Number of papers
- Number of authors
- Median and mean number of citations after 5 years
- Maximal number of citations after 5 years

The full features of >2,600 L3 fields of study are presented in Table 1.

**Table 1:** L3 Fields-of-Study Features in 2009

		Citations after 5 years			
Parent field of study	Field of study name	Median	Maximum	No. of papers	Mean No. of authors
Engineering	Structural material	61.0	1,250	174	6.14
Biology	Genetic recombination	50.5	451	196	6.07
Biology	Nature	48.0	5,660	4,162	6.28
Biology	microRNA	47.0	3,076	1,691	6.24
Biology	Induced pluripotent stem cell...	39.0	987	213	6.53
Economics	Signalling	39.0	695	1,030	5.87
Biology	Genome evolution	35.5	392	140	5.04
Biology	Non-coding RNA	35.0	1,414	375	5.39
Biology	Post-transcriptional modification...	34.0	1,414	315	5.49
Biology	Autophagy	34.0	789	381	5.71
Mathematics	Finite impulse response	2.0	167	337	3.00
Computer science	Pixel	2.0	380	2,484	3.27
Computer science	Ontology	2.0	616	733	3.35
Computer science	Mesh networking	2.0	62	274	3.43
Computer science	Camera resectioning	2.0	43	114	3.13
Computer science	Session initiation protocol...	2.0	116	100	3.60
Chemistry	Gallium	2.0	73	484	3.43
Mathematics	Presentation of a group	2.0	91	706	3.22
Mathematics	Spiral	2.0	80	122	3.65
Mathematics	Block code	2.0	54	281	2.83

The results of the above-described calculations are presented in the Results of Fields-of-Research Trends section. Moreover, the code implementation is provided in the “Part III - D: Analyzing Changing Trends in Academia - Research Fields” Jupyter Notebook (see the Availability of Source Code and Requirements section).

## Results

In the following subsections, we present all the results for the experiments that were described in the Analysis of Publication Trends section. Additional results are presented in the Supplementary Materials.

### Results of paper trends

**Figure 1:**
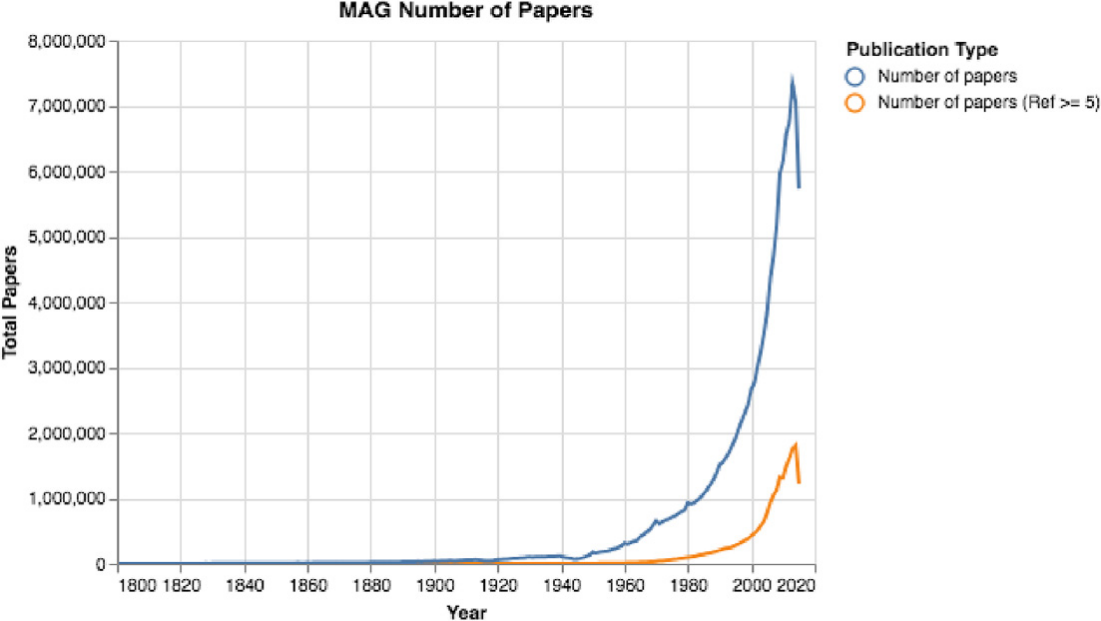
The number of papers over time. The total number of papers has surged exponentially over the years.

**Figure 2:**
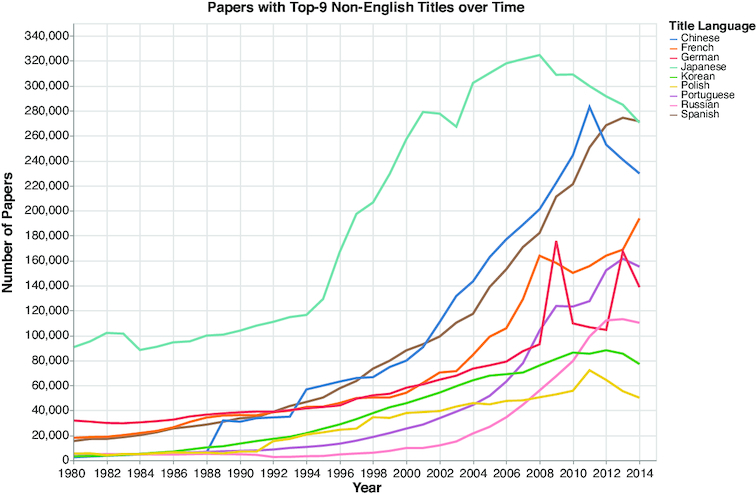
Papers with titles in the top 9 non-English languages. An increasing number of papers have non-English titles.

In recent years there has been a surge in the number of published academic papers, with >7 million new papers each year and >1.8 million papers with ≥5 references (see Fig. 1).(There is a decline in the number of papers after 2014, probably due to missing papers in the MAG dataset, which was released in 2016.) Additionally, by analyzing the language of the papers’ titles, we observed an increase in papers with non-English titles (see Fig. 2).

**Figure 3:**
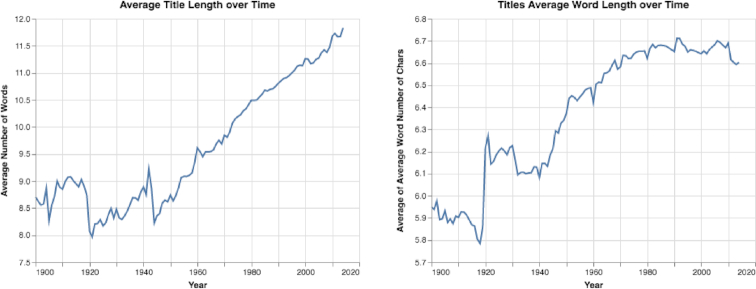
Mean title length over time. A paper’s mean title length increased from 8.71 words to 11.83 words. Moreover, the mean word length increased from 5.95 characters to 6.6 characters per title word.

As described in the Paper Trends section, we analyzed how various properties of academic papers have changed over time to better understand how papers’ structures have evolved. In this analysis, we discovered that papers’ titles became longer, from a mean of 8.71 words in 1900 to a mean of 11.83 words in 2014 (see Fig. 3). Moreover, the mean number of characters per title word increased from 5.95 in 1900 to 6.6 in 2014 (see Fig. 3). Additionally, we observed that in recent years the percentage of papers with question or exclamation marks in their titles increased sharply, from <1% of all papers in 1950 to >3% of all papers in 2013 (see Fig. S2). Furthermore, the use of interrobangs (represented by ?! or !?) in titles also increased sharply, from 0.0005% in 1950 to 0.0037% in 2013 (see Fig. S2).

**Figure 4:**
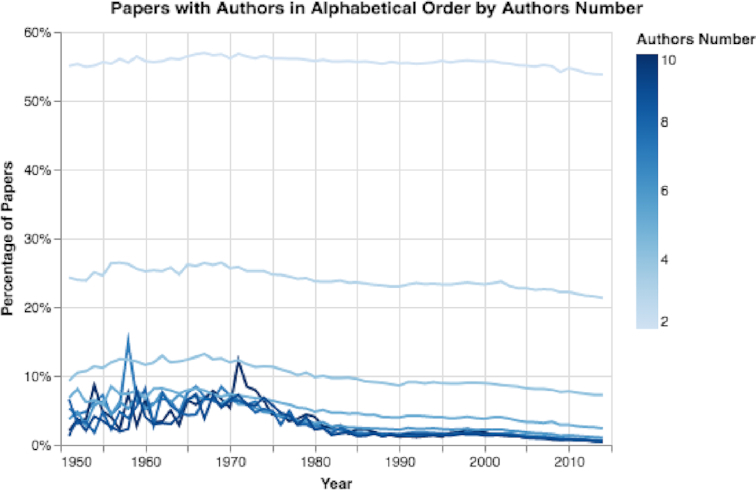
Percentage of papers with author lists in alphabetical order, grouped by the number of authors. The more authors, the less likely the authors will be listed alphabetically in the byline.

We explored how the number and order of the author list has changed over time. The number of authors for papers with ≥5 references more than tripled over the years, from a mean of 1.41 authors to a mean of 4.51 authors per paper between 1900 and 2014 (see Fig. S3). Also, the maximal number of authors for a single paper in each year increased sharply over time, especially in recent years (see Fig. S4). In fact, some recent papers actually listed >3,000 authors. Moreover, we observed that the percentage of author lists ordered alphabetically decreased in recent years, from 43.5% of all papers published in 1950 to 21.0% of all papers published in 2014 (see Fig. S5). Furthermore, we discovered that with a higher number of authors, it is less likely that the author list will be ordered alphabetically (see Fig. 4). For example, in 2014 only ∼1% of papers with 6 authors were ordered alphabetically.

**Figure 5:**
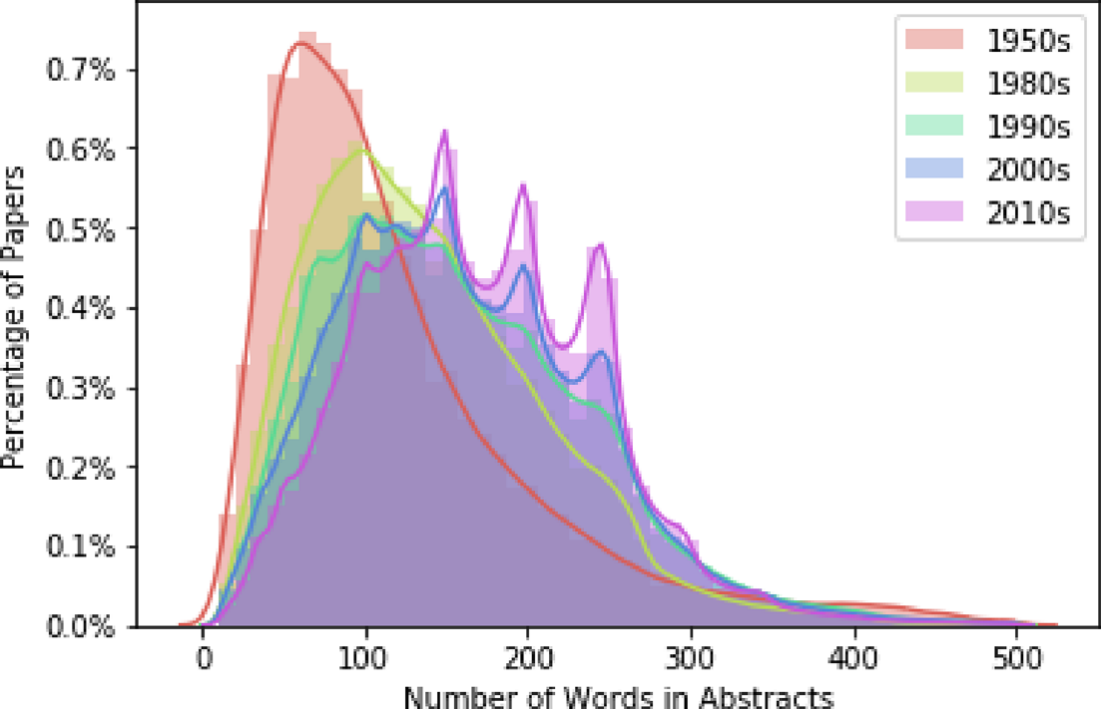
Distribution over time of the number of words in abstracts. Over time, papers’ abstracts have tended to become longer.

When calculating how the abstracts of papers have changed over time, we discovered that the abstract length increased from a mean of 116.3 words in 1970 to a mean of 179.8 words in 2014 (see Fig. S6). Moreover, with each decade since 1950, the distributions shifted to the right, showing that papers with longer abstracts of 400 and even 500 words have become more common over time (see Fig. 5). Additionally, we analyzed how the number of keywords in papers has changed. We discovered that both the number of papers containing keywords increased, as well as the mean number of keywords per paper (see Fig. S7).

**Figure 6:**
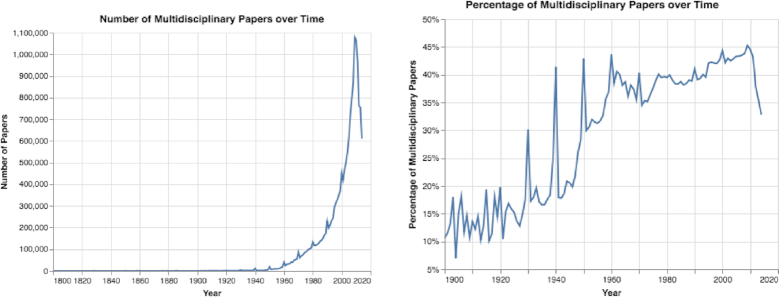
The number and percentage of multidisciplinary papers over time. Between 1900 and 2010, both the number and percentage of multidisciplinary papers increased.

By estimating the percentage and number of multidisciplinary papers over time, we discovered an increase in the number of multidisciplinary papers until 2010, followed by a sharp decrease (see Figs 6 and S8). After performing further analysis, we believe the decline in the number of multidisciplinary papers is a result of papers with missing keywords in the MAG dataset, such as papers that were published in *PLoS One*. These papers have dynamically changing keywords in the online version but not in the offline version.

By examining how the number of references has changed over time, we observed a sharp increase in the mean number of references per paper (see Fig. S9). In addition, by analyzing the reference number distributions grouped by publishing decade, we can observe that higher numbers of references have become increasingly common. For example, in 1960 few papers had >20 references, but by 2010 many papers had >20 references, and some >40 references (see Fig. S10).

**Figure 7:**
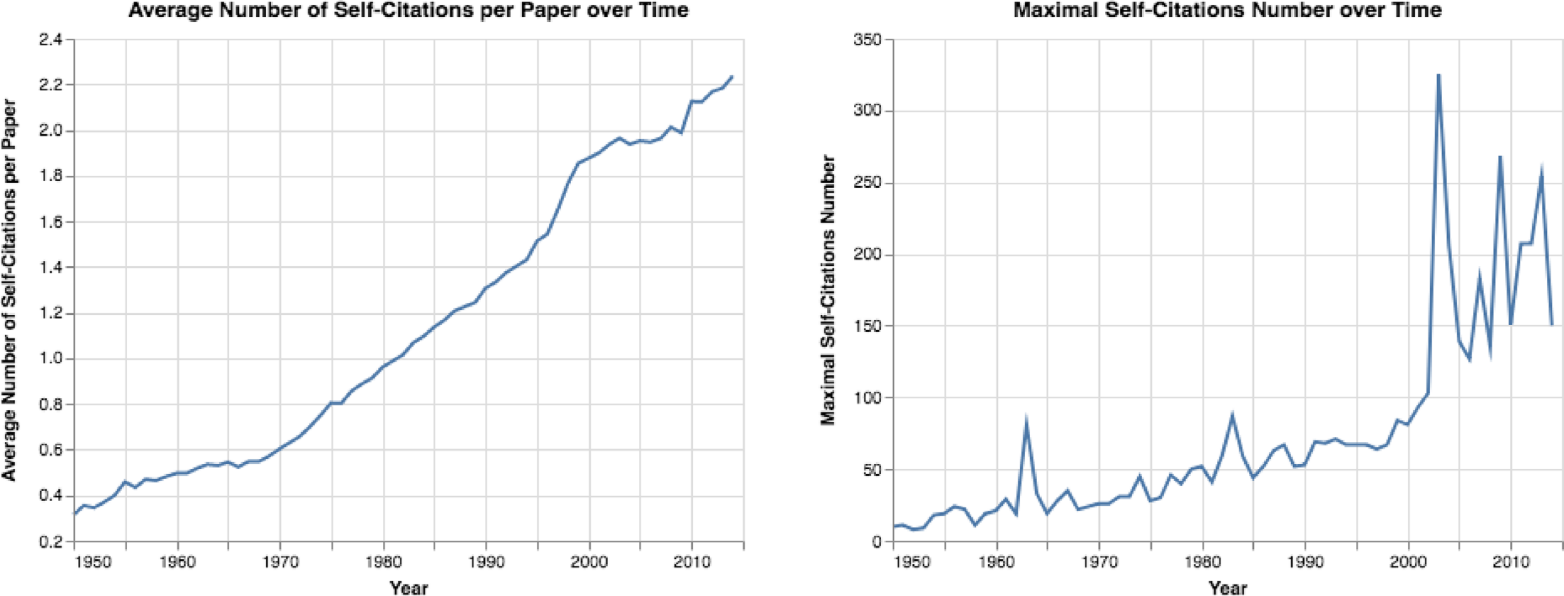
The mean and maximal number of self-citations. Both the mean and maximal number of self-citations increased over time.

We also examined how self-citation trends have changed, and we observed that both the total number of self-citations and the percentage of papers with self-citations increased substantially (see Fig. S12). Also, the mean number of self-citations per paper, as well as the maximal number of self-citations in each year, increased sharply (see Fig. 7). For example, ∼3.67% of all papers in 1950 contained ≥1 self-citation, while 8.29% contained self-citations in 2014 (see Fig. S12). Moreover, the maximal number of self-citations in a single paper increased sharply from 10 self-citations in a paper published in 1950 to >250 self-citations in a paper published in 2013 (see Fig. 7).

**Figure 8:**
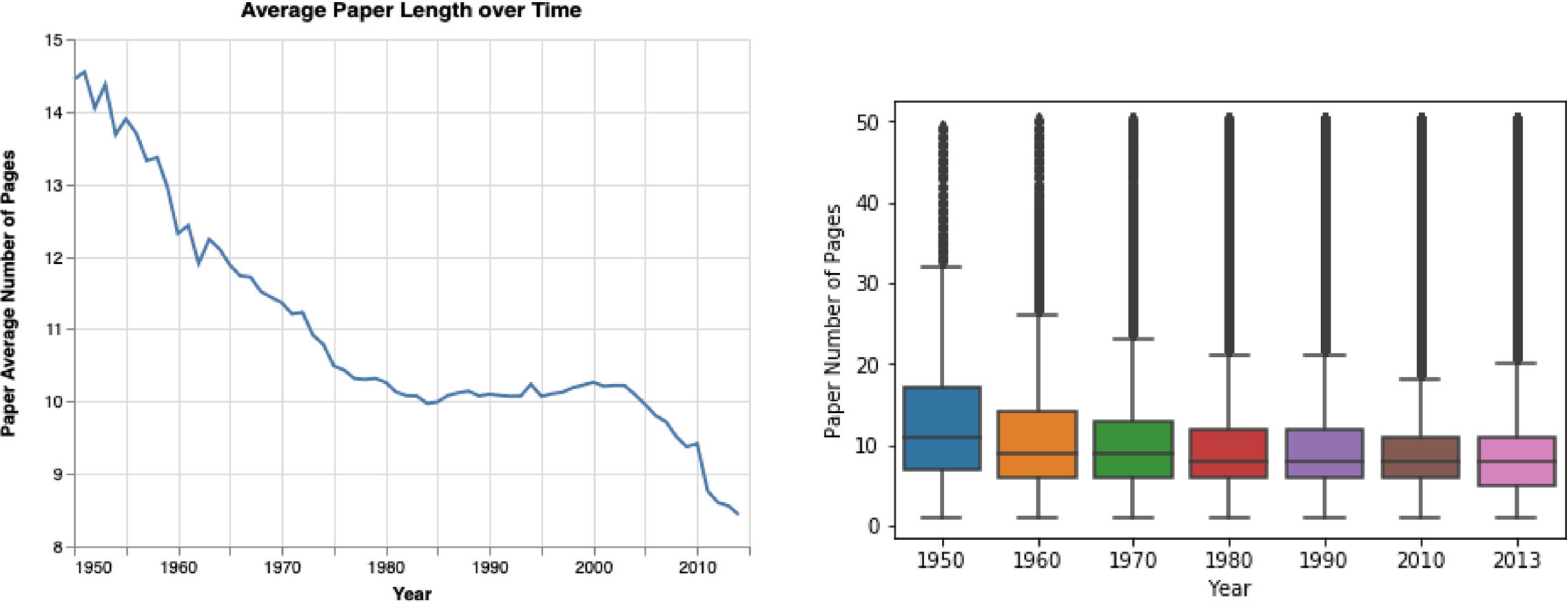
Paper's lengths. Both the papers’ mean and median lengths decreased over time. In the right panel, the horizontal line indicates the median, and the box encloses the interquartile range.

By using the AMiner dataset to analyze how papers’ lengths have changed, we discovered that the mean and median length of papers decreased over time (see Fig. 8). The mean length of a paper was 14.4, 10.1, and 8.4 pages in 1950, 1990, and 2014, respectively.

**Figure 9:**
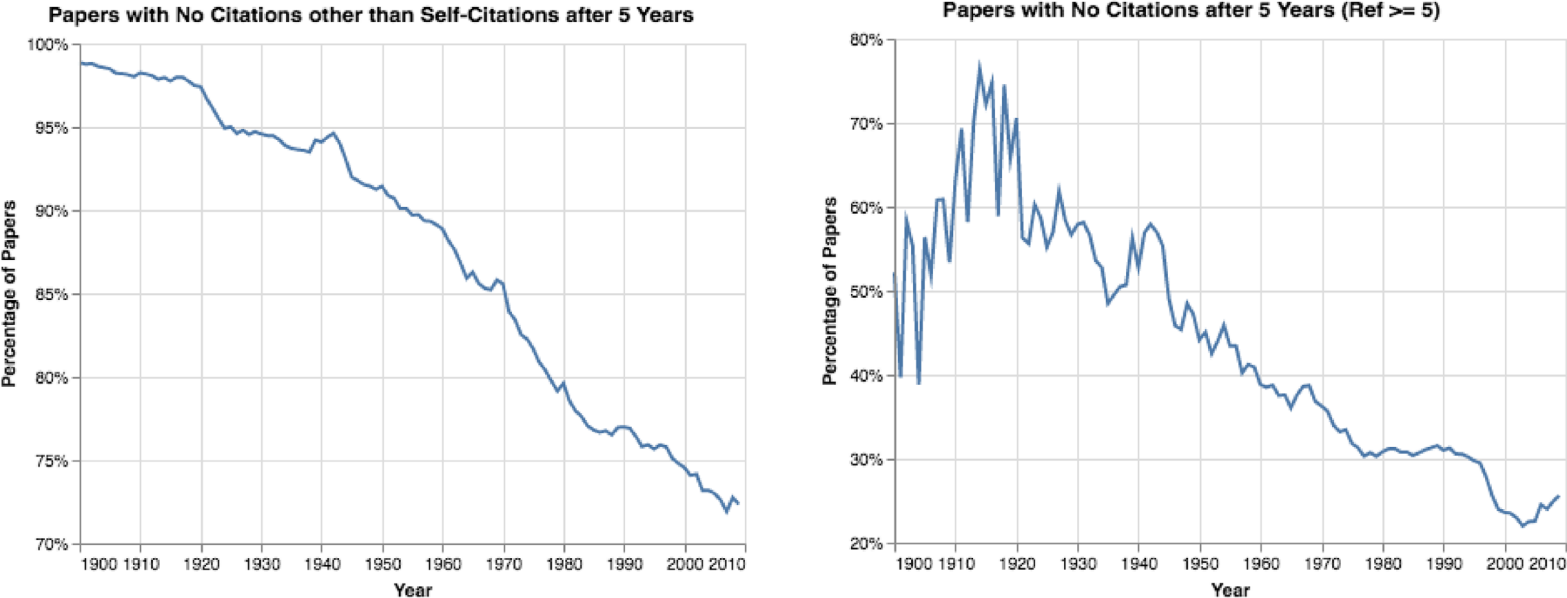
Papers with no citations other than self-citations after 5 years. The percentage of papers with no citations after 5 years decreased; nevertheless, 72.1% of all papers published in 2009 had no citations after 5 years.

By analyzing citation patterns over time, we discovered that the percentage of papers with no citations other than self-citations after 5 years decreased (see Fig. 9). Nevertheless, 72.1% of all papers published in 2009, and 25.6% of those with ≥5 references, were still without any citations after 5 years (see Fig. 9). Moreover, the total number of papers without any citations increased sharply (see Fig. S11).

**Figure 10:**
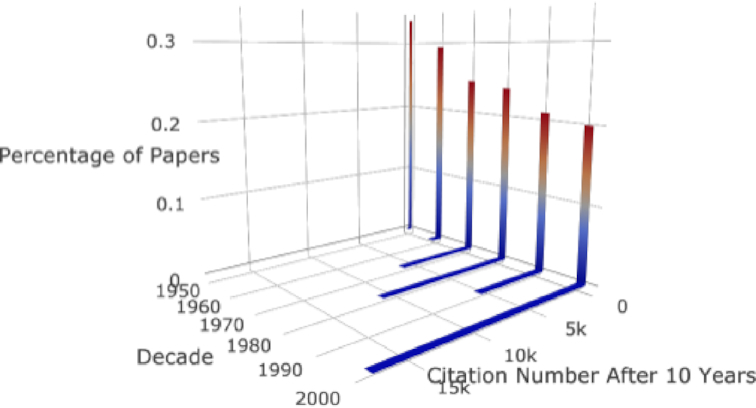
Citation distributions over time. The citation distributions of different decades show notable changes.

Additionally, by analyzing the citation distributions of papers published in different decades, we discovered that citation distributions changed notably over time (see Fig. 10).

Last, using the properties of >3.29 million papers published between 1950 and 2009, we discovered positive correlations among the papers’ citation numbers after 5 years and the following features: (i) title lengths (τ_*s*_ = 0.1), (ii) number of authors (τ_*s*_ = 0.22), (iii) abstract lengths (τ_*s*_ = 0.26), (iv) number of keywords (τ_*s*_ = 0.15), (v) number of references (τ_*s*_ = 0.48), (vi) paper lengths (τ_*s*_ = 0.13), and (vii) use of question or exclamation marks in the title (τ_*s*_ = 0.022) (see Fig. S13). (Similar correlation values were obtained by calculating the correlations for papers published in a specific year.)

### Results of author trends

**Figure 11:**
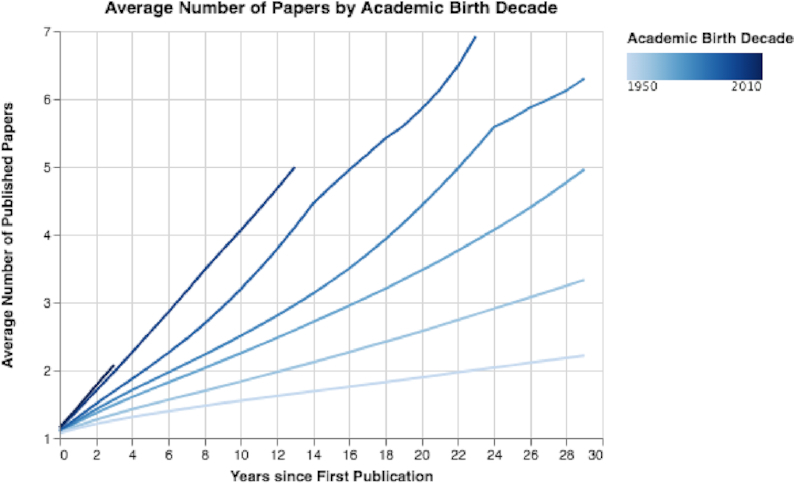
Mean number of papers by authors’ academic birth decades. With each decade, the rate of paper publication has increased.

By analyzing the number of new authors each year, we discovered a sharp increase over time, with several million new authors publishing each year in recent years (see Fig. S14). (However, it is possible that the same author has several MAG author IDs.) Additionally, when analyzing the trends grouped by the authors’ academic birth decades, we discovered a substantial increase in the mean number of published papers for the later birth decades (see Fig. 11). For example, researchers who started their careers in 1950 published on average 1.55 papers in a period of 10 years, while researchers who started their careers in 2000 published on average 4.05 papers in the same time frame. Furthermore, we observed that authors who started their careers after 1990 tended to publish more in conferences in the first years of their career than their more senior peers who started their careers in the 1950s or 1970s (see Fig. S15). For example, researchers who started their careers in the 1970s published on average ∼2 conference papers and 1.65 journal papers after 10 years; researchers who started their careers in the 2000s published ∼4 conference papers and 2.59 journal papers in the same time frame.

**Figure 12:**
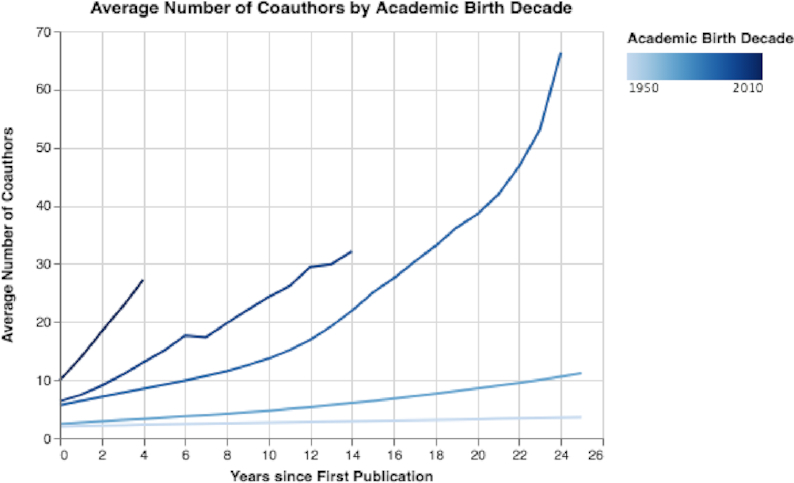
Mean number of co-authors by academic birth decade. The mean number of co-authors has considerably increased over the decades.

We can also observe that the mean number of co-authors has considerably increased over the decades (Fig. 12). Moreover, we note that researchers who started their careers in the 1950s and 1970s had on average only a few co-authors over a period of 25 years, while researchers who started their careers in the 1990s had >60 co-authors in the same career length of 25 years (see Fig. 12).

**Figure 13:**
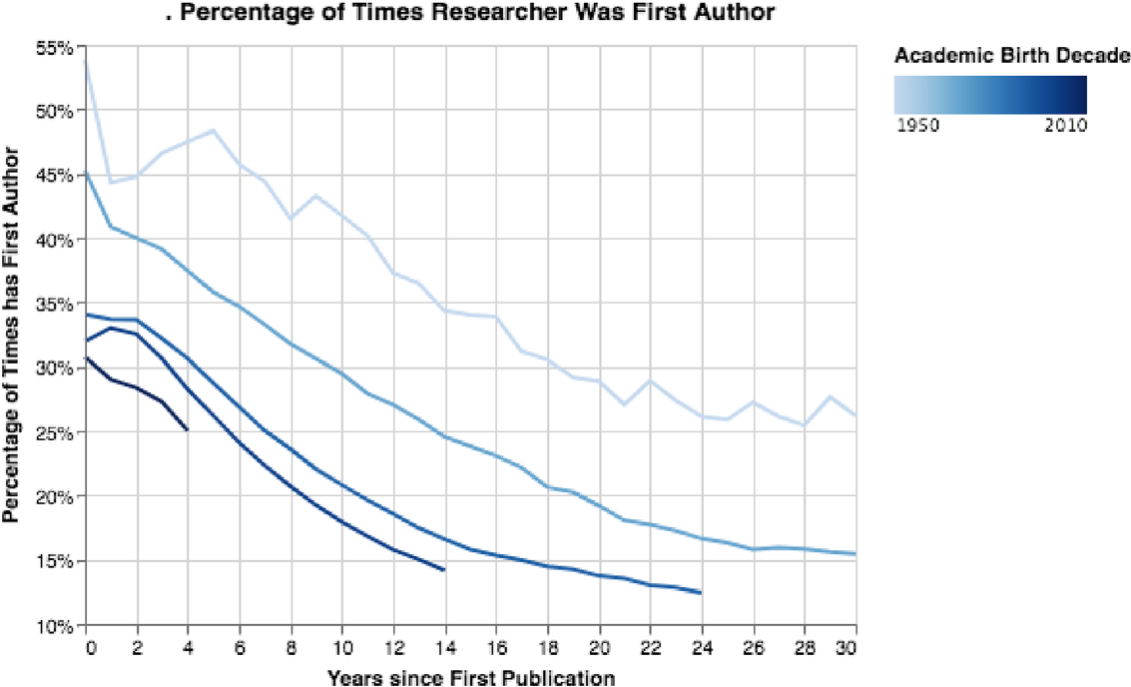
Percentage of times researcher was first author. We can observe that over time on average the percentage of senior researchers as first authors declined. Moreover, in the same time intervals, the percentage of times recent generations of researchers were first authors declined compared to older generations.

Last, by exploring how author sequence numbers evolved, we discovered that with seniority, the researchers’ median sequence number increased (see Fig. S16). Additionally, with seniority, the percentage of published papers with the researcher listed as the first author decreased (Fig. 13). Moreover, by looking at the decade researchers started their careers, we can see a sharp decline in the percentages of first authors (Fig. 13). Overall, early-career researchers are publishing more in their careers but appear as first authors much less than in previous generations.

### Results of journal trends

**Figure 14:**
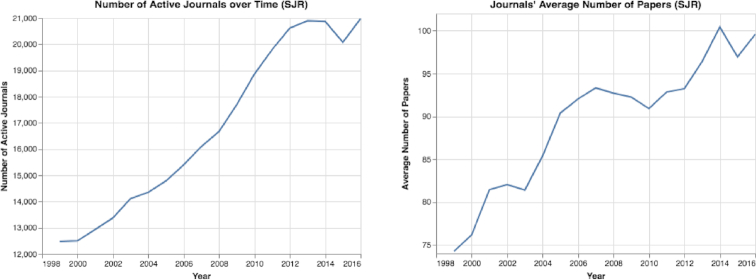
Number of active journals over time. Over a period of 18 years, from 1999 to 2016, both the number of active journals and the papers per journal increased greatly.

By analyzing journal trends using the SJR and MAG datasets, we discovered that the number of journals increased greatly over the years, with 20,975 active ranked journals in 2016 (Fig. 14). Furthermore, we observed that hundreds of new ranked journals were published each year (see Figs S17 and S18). In addition, we discovered that the number of published papers per journal increased sharply, from a mean of 74.2 papers in 1999 to a mean of 99.6 papers in 2016 (Fig. 14). We also observed that in recent years, journals that publish thousands of papers have become more common. For example, in 2016, according to the SJR dataset, 197 journals published >1,000 papers each.

**Figure 15:**
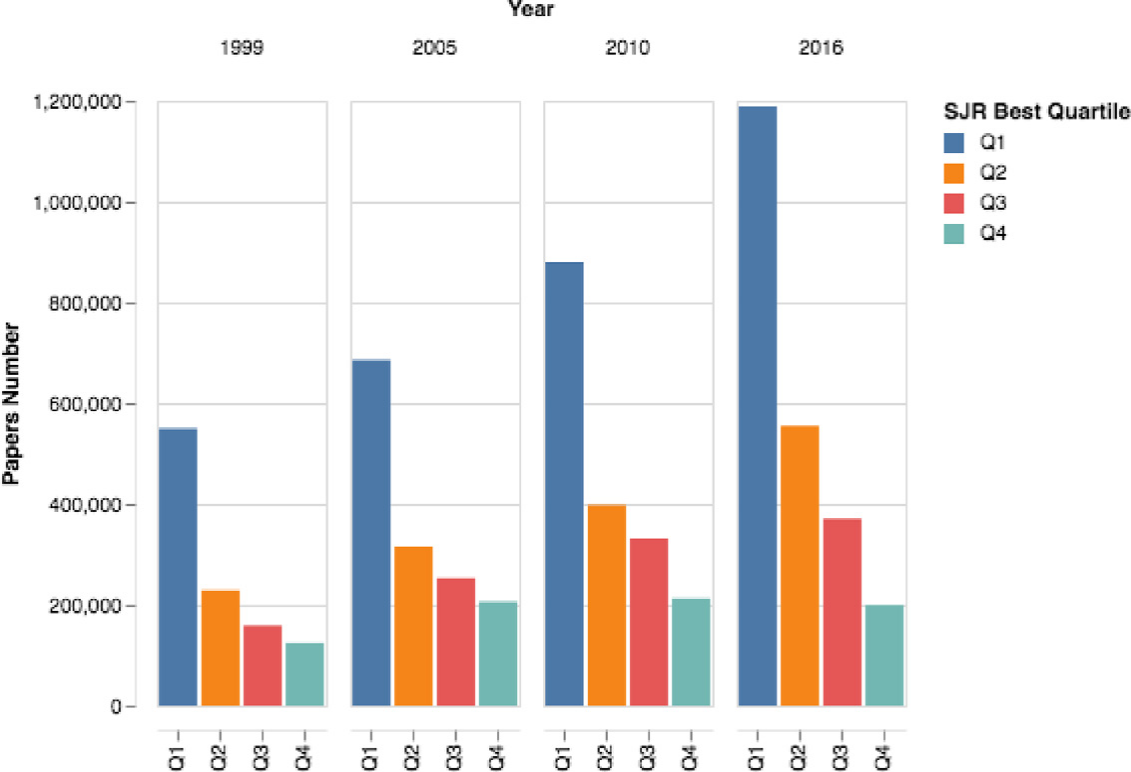
Journals’ quartile number of papers over time. The number of papers published in Q1 journals has vastly increased.

**Figure 16:**
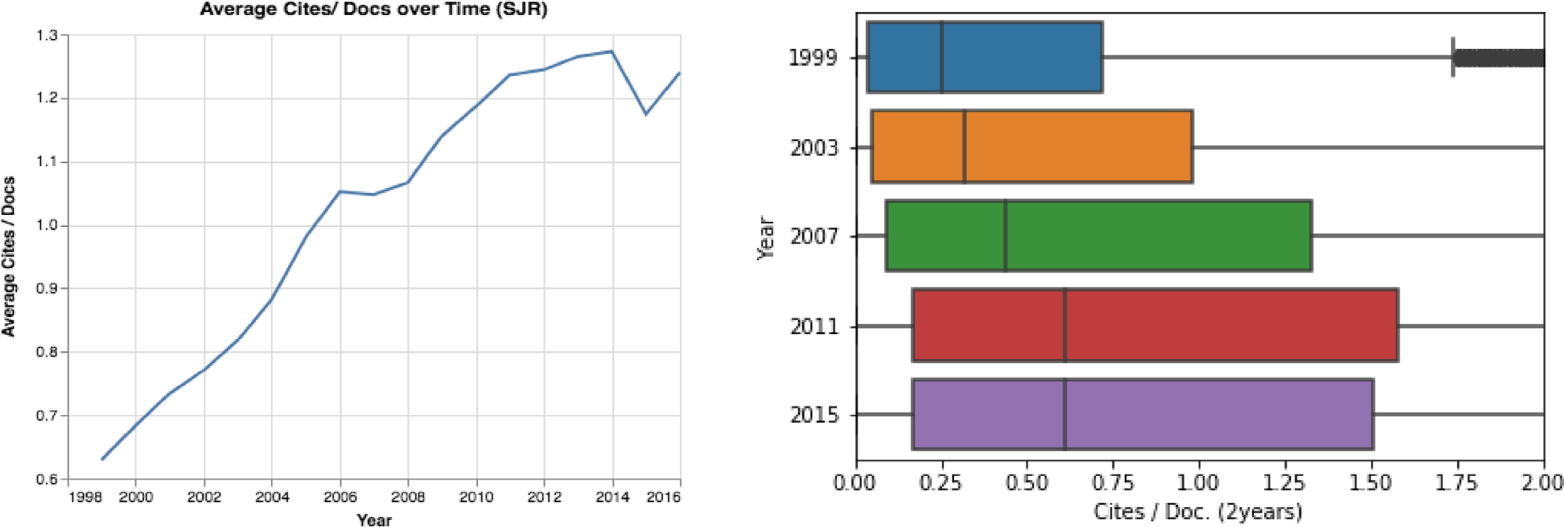
The mean number of citations [Citation Number/Documents Number (2 years)] over time. The mean number of citations values have almost doubled in the past 18 years; additionally, their distributions have changed considerably.

By exploring how various metrics have changed over time, we discovered the following: First, over the past 18 years, the number of papers published in Q1 and Q2 journals more than doubled, from 550,109 Q1 papers and 229,373 Q2 papers in 1999 to 1,187,514 Q1 papers and 554,782 Q2 papers in 2016 (Fig. 15). According to the SJR dataset, in 2016, 51.3% of journal papers were published in Q1 journals and only 8.66% were published in Q4 journals. Second, the h-index decreased over recent years from a mean value of 37.4 and median of 23.0 in 1999 to a mean value of 31.3 and median of 16 in 2016 (see Fig. S19). Third, we noted that the SJR and the mean number of citations measures both increased considerably during the past 18 years (see Figs 16 and S20).

**Figure 17:**
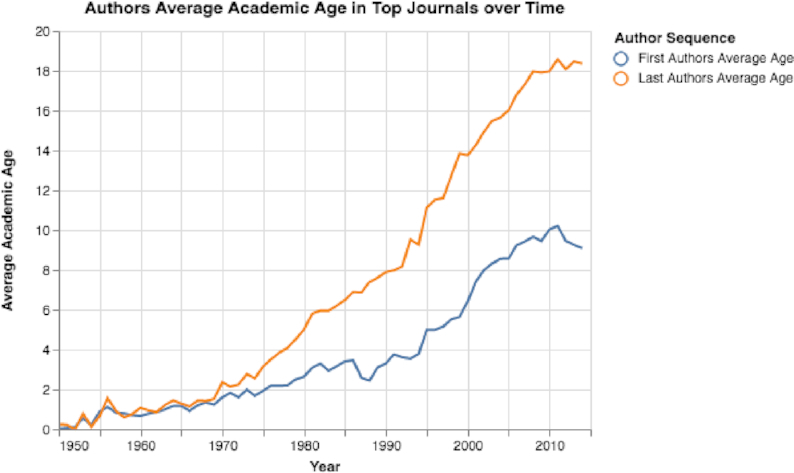
Top-selected journals’ mean first and last authors ages. Both the first and last authors’ mean ages have increased sharply.

**Figure 18:**
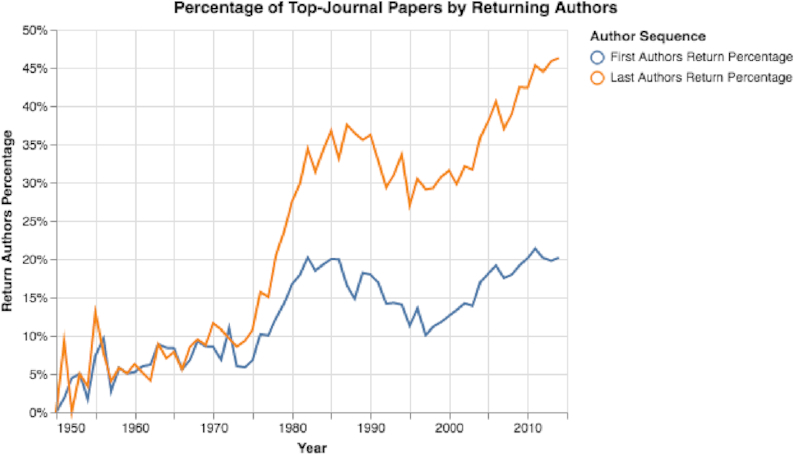
Percentage of papers with returning first or last authors. The percentage of returning first or last top-journal authors increased considerably.

Besides the number of papers in top journals doubling between 2000 and 2014, the number of authors increased substantially (see Fig. S21). (The total number of authors each year was determined by summing the number of authors in each published paper.) Additionally, by calculating the mean academic career ages of first and last authors, we discovered that in recent years the mean academic age has increased notably (Fig. 17). Moreover, when looking at first and last authors who previously published in one of the selected top-30 journals, we discovered that over time the percentage of returning authors increased substantially (see Fig. 18). By 2014, 46.2% of all published papers in top-30 selected journals were published by last authors who had published ≥1 paper in a top-30 selected journal before (Fig. 18).

**Figure 19:**
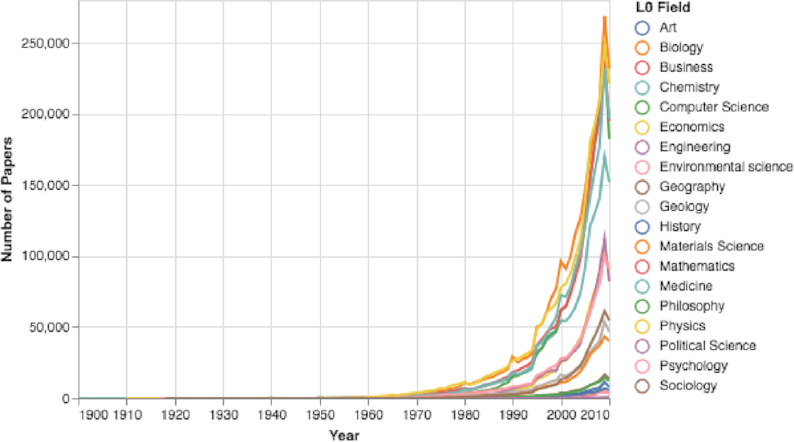
Mean percentage of return authors in top-selected journals over time. In most journals the number of papers with ≥1 author who previously published in the journal increased sharply. In many of the selected journals the percentage of papers with returning authors was >60%, and in some cases >80%.

By calculating the number of papers, number of authors, authors’ mean age, and percentage of returning authors in each selected top-30 journal, we observed the following: (i) the number of published papers per year increased considerably in the vast majority of the journals (see Fig. S22); (ii) the mean career ages of last authors in the vast majority of the selected journals considerably increased (see Fig. S23); e.g., in *Cell*, the last authors’ career ages increased from ∼4.5 years in 1980 to ∼20 years in 2014 (see Fig. S23); and (iii) the percentage of returning authors in the vast majority of the selected journals increased drastically; e.g., in *Nature Genetics*, in 86.6% of 2014 papers, ≥1 of the authors had published in the journal before (see Fig. 19).

### Results of fields-of-research trends

**Figure 20:**
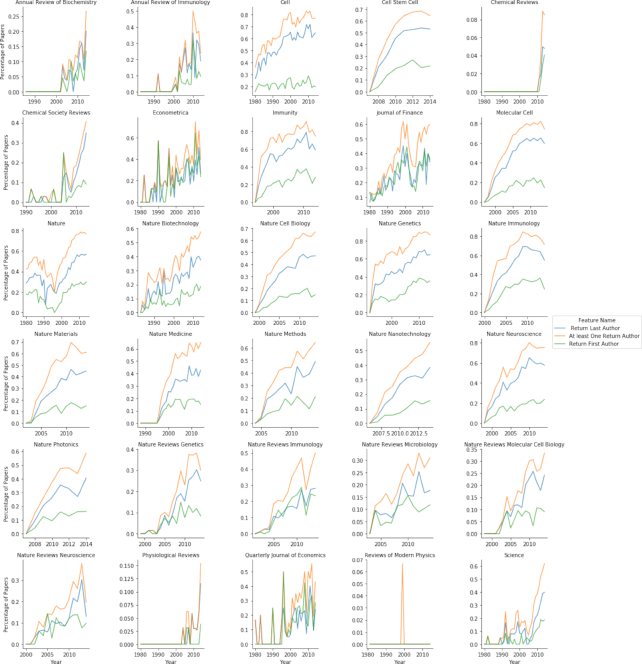
L0 Fields-of-study number of papers over time. The numbers of papers in each field of study have increased drastically.

**Figure 21:**
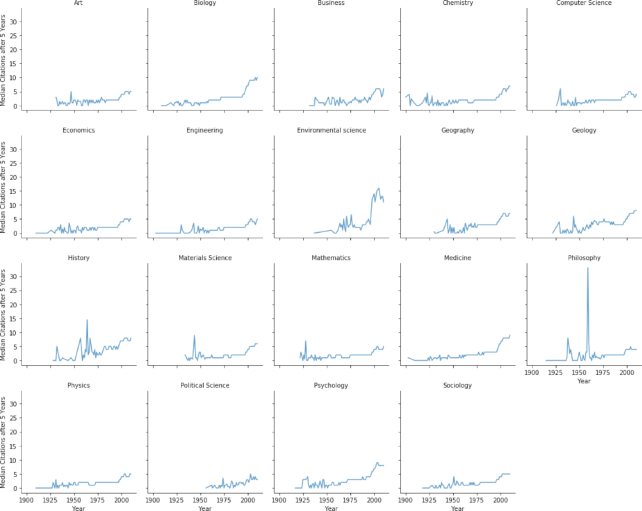
L0 Field-of-study median citation number after 5 years. There is notable variance among the L0 fields-of-study median citation numbers.

By matching each paper to its L0 field of study and analyzing each field’s properties, we discovered substantial differences in these properties. Namely, we observed the following:

- A large variance in the number of published papers in each field. For example, 231,756 papers were published in the field of biology in 2010, but only 5,684 were published that year in the field of history (see Figs 20: and S24).
- A considerable variance in the mean number of paper authors among the various research fields. For example, the number of authors in 2010 ranged from a mean of 2.28 authors in the field of political science to a mean of 5.39 authors in medicine (see Fig. S25).
- A variance in the papers’ mean number of references in different fields. For example, in 2010, the mean reference number in the fields of material science and engineering was <24, while in the fields of biology and history it was >33 (see Fig. S26).
- A big variance in each L0 field’s mean and median number of citations after 5 years. For example, for 2009 papers in the fields of computer science and political science, the median number of citations 5 years after publication was 4, while in biology and environmental science, the median was 9 and 13 citations, respectively (Fig. 21).

By repeating the above analysis for the L1 subfields of biology and for the L2 subfields of genetics, we uncovered similar differences among fields of study. Namely, we observed the following for subfields in the same hierarchal level: (i) significant variance in the mean number of papers (see Figs S27 and S28), (ii) notable variance in the mean number of authors (see Figs S29 and S30), (iii) noteworthy variance in the mean number of references (see Figs S31 and S32), and (iv) vast variance in median citation numbers (see Figs S33 and S34).

Last, by analyzing various features of 2,673 L3 fields of study, we observed a huge variance in the different properties (see Table 1 and Fig. S35). For example, several fields of study, such as gallium (chemistry), ontology (computer science), and presentation of a group (mathematics), had median citation numbers of 2, while other fields of study, such as microRNA and genetic recombination (biology), had median citation numbers of >47 and 50.5, respectively (see Table 1 and Fig. S35).

By analyzing the results presented in the Results section, the following can be noted: first, we observed that the structure of academic papers has changed in distinct ways in recent decades. While the mean overall length of papers has become shorter (see Fig. 8), the title, abstract, and references have become longer (see the Results of Paper Trends section and Figs 3, 5, S3, S6, S9, and S10). Also, the number of papers that include keywords has increased considerably, as has the mean number of keywords in each paper (see Fig. S7). Furthermore, the mean and median number of authors per paper has increased sharply (see Figs S3 and S4).

## Discussion

Below we discuss 9 aspects of our study that provide insights into current academic publishing trends, and we explore the potential impact of our results.

First, these results support Goodhart’s Law as it relates to academic publishing: the measures (e.g., number of papers, number of citations, h-index, and impact factor) have become targets, and now they are no longer good measures. By making papers shorter and collaborating with more authors, researchers are able to produce more papers in the same amount of time. Moreover, we observed that the majority of changes in papers’ properties are correlated with papers that receive higher numbers of citations (see Fig. S13). Authors can use longer titles and abstracts, or use question or exclamation marks in titles, to make their papers more appealing. Thus, more readers are attracted to the paper, and ideally they will cite it, i.e., academic clickbait [45]. These results support our hypothesis that the citation number has become a target. Consequently, the properties of academic papers have evolved in order to win—to score a bullseye on the academic target.

It is worth noting that while the study’s results provide evidence that many citation-based measures have become targets, there also may be other factors that influence academic publication trends. For example, the academic hypercompetitive environment itself may prompt an increase in productivity [81], hence increasing the number of papers. However, this claim contradicts the findings of Fanelli and Larivière that researchers’ individual productivity did not increase in the past century [52]. Nevertheless, it is important to keep in mind that there may be other underlying factors that contributed to the observed results.

Second, we observed that over time fewer papers list authors alphabetically, especially papers with a relatively high number of authors (see Results of Paper Trends section and Figs 4 and S5). These results may indicate the increased importance of an author’s sequence number in the author list, which may reflect the author’s contribution to the study. This result is another signal of the increasing importance of measures that rate an individual’s research contribution.

Third, from matching papers to their L0 fields of study, we observed that the number of multidisciplinary papers has increased sharply over time (see Fig. 6). It is important to keep in mind that these results were obtained by matching keywords to their corresponding fields of study. Therefore, these results have several limitations: first, not all papers contain keywords. Second, the dataset may not extract keywords from papers in the correct manner. For example, we found some papers contained keywords in their online version but not in their offline version (see Results of Paper Trends section). It is also possible that in some fields it is less common to use keywords. Therefore, the papers’ keywords may be missing in the datasets, and the presented results may be an underestimate of the actual number of multidisciplinary studies. Nevertheless, we observed a strong trend in increasing numbers of multidisciplinary papers.

Fourth, from seeing sharp increases in both the maximal and mean number of self-citations (see Results of Paper Trends section and Figs 7, 9, 10, and S12), it is clear that citation numbers have become a target for some researchers, who cite their own papers dozens, or even hundreds, of times. Furthermore, we observed a general increasing trend for researchers to cite their previous work in their new studies. Moreover, from analyzing the percentage of papers without citations after 5 years, we observed that a huge quantity of papers (>72% of all papers and 25% of all papers with ≥5 references) have no citations at all (see Fig. 9). Obviously, many resources are spent on papers with limited impact. The lack of citations may indicate that researchers are publishing more papers of poorer quality to boost their total number of publications. Additionally, by exploring papers’ citation distributions (see Fig. 10), we can observe that different decades have very different citation distributions. This result indicates that comparing citation records of researchers who published papers during different periods can be challenging.

Fifth, by exploring trends in authors (see Results of Author Trends section and Figs 11, 12, 13, S14, S15, and S16), we observed an exponential growth in the number of new researchers who publish papers. We also observed that young career researchers tend to publish considerably more than researchers in previous generations, using the same time frames for comparison (see Fig. 11). Moreover, young career researchers tend to publish their work much more in conferences in the beginning of their careers than older researchers did in previous decades (see Fig. S15). We also observed that young career researchers tend to collaborate considerably more in the beginning of their careers than those who are older (see Fig. 12). Furthermore, we see that the mean percentage of researchers as first authors early in their career is considerably less than those in previous generations (see Fig. 13). In addition, authors’ median sequence numbers typically increase over time, and the rate is typically faster for young career researchers (see Fig. S16). These results emphasize the changes in academia in recent years. In a culture of “publish or perish,” researchers publish more by increasing collaboration (and being added to more author lists) and by publishing more conference papers than in the past. However, as can be observed by the overall decline of researchers as first authors, young career researchers may be publishing more in their careers but contributing less to each paper. The numbers can be misleading: a researcher who has 5 “first author” claims but has published 20 papers may be less of a true contributor than one with 4 “first author” claims and 10 published papers.

Sixth, by analyzing journal trends (see Results of Journal Trends section), we see a rapid increase in the number of ranked active journals in recent years (see Fig. 14). Moreover, on average, journals publish more papers than in the past, and dozens of journals publish >1,000 papers each year (see Figs 14 and S17). With the increase in the number of active journals, we observed rapid changes in impact measures: (i) the number of papers published in the first and second quartiles (Q1 and Q2) has increased sharply, and today the vast majority of papers are published in these quartiles (see Fig. 15); (ii) the journals’ mean and median h-index have decreased sharply (see Fig. S18); and (iii) both the SJR and the mean number of citations have increased considerably (see Figs 16 and S20). With these substantial changes, it is clear that some measures, such as the use of quartiles and the h-index, are rapidly losing meaning and value. Moreover, with the abundance of journals, researchers can “shop around” for a high-impact journal and submit a rejected paper from one Q1 journal to another Q1 journal, time after time, and then start the review process again. These repeated reviews for the same paper waste time, and in the long run the burden of reviewing papers several times may affect the quality of the reviews.

There are compelling reasons to change the current system. We need to think about making all reviews open and online. We should consider the function of published journals; for that matter, is it even necessary to have journals in a world with >20,000 journals that publish hundreds or even thousands of papers each year? We need to seriously evaluate the measures we use to judge research work. If all these measures have been devalued to being merely targets, they are no longer effective measures. Instead, they should be adapted to meet our current needs and priorities. Moreover, today there are alternative measures to evaluate researchers’ contributions and journals’ impacts (see Background section). It would be beneficial to the academic community to promote the use of these measures, while concurrently raising awareness of the many limitations of the traditional measures that are still commonly used.

Seventh, by focusing on trends in selected top journals, we can observe that these journals have changed considerably in recent years (see Figs 17, 18, 20, S21, and S22). The number of papers in the selected journals has increased sharply, along with the career age of the authors and the percentage of returning authors. The number of submissions to top journals, such as *Nature*, have increased greatly in recent years [82]; however, many of these journals mainly publish papers in which ≥1 of the authors has previously published in the journal (see Figs 18 and 20). We believe that this situation is also a result of Goodhart’s Law. The target is the impact factor, and so researchers are vigorously seeking journals with high impact factors. Therefore, the yearly volume of papers sent to these top journals has considerably increased, and, overwhelmed by the volume of submissions, editors at these journals may choose safety over risk and select papers written by only well-known, experienced researchers.

Eighth, by analyzing how features evolve in the various L0 fields of study using the MAG dataset, we can observe that different fields have completely different sets of features (see Figs 20, 21, 19, S25, S26, and Table 1). While some fields have hundreds of thousands of papers published yearly, others have only thousands published yearly (see Figs 20 and S22). Moreover, similar large differences are reflected in other examined fields’ features, such as the mean number of references and the mean and median citation numbers (see Figs 21 and S35).

Last, by examining >2,600 research fields of various scales (see Table 1 and Fig. S35), we observed vast diversity in the properties of papers in different domains: some research domains grew phenomenally while others did not. Even research domains in the same subfields presented a wide range of properties, including papers’ number of references and median number of citations per research field (see Table 1 and Figs S31, S32, S33, and S34). These results indicate that measures such as citation number, h-index, and impact factor are useless when comparing researchers in different fields, and even for comparing researchers in the same subfield, such as genetics. These results emphasize that using citation-based measures for comparing various academic entities is like comparing apples to oranges, and is to “discriminate between scientists.” [55]. Moreover, using these measures as gauges to compare academic entities can drastically affect the allocation of resources and consequently damage research. For example, to improve their world ranking, universities might choose to invest in faculty for computer science and biology, rather than faculty for less-cited research fields, such as economics and psychology. Moreover, even within a department, the selection of new faculty members can be biased due to using targeted measures, such as citation number and impact factor. A biology department might hire genetic researchers in the field of epigenetics, instead of researchers in the field of medical genetics, due to the higher mean number of citations in the epigenetics field. Over time, this can unfairly favor high-citation research fields at the expense of other equally important fields.

## Conclusions

**Figure 22:**
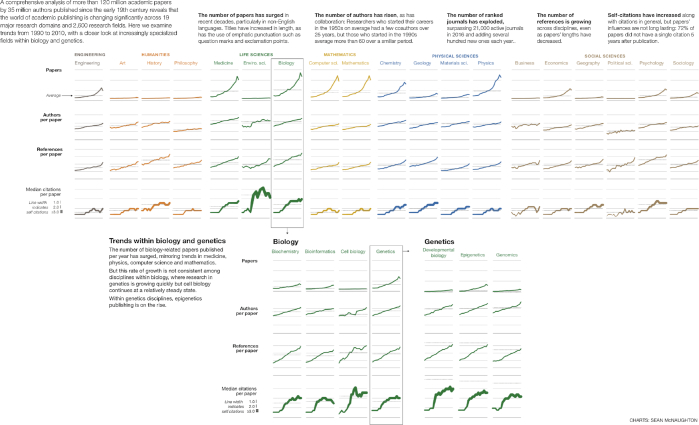
Measuring success in academic publishing.

In this study, we performed a large-scale analysis of academic publishing trends, examining data on >120 million papers and >20,000 journals. By analyzing this huge dataset, we can observe that over the past century, especially the past few decades, published research has changed considerably, including the numbers of papers, authors, and journals; the lengths of papers; and the mean number of references in specific fields of study (Fig. 22).

While the research environment has changed, many of the measures to determine the impact of papers, authors, and journals have not changed. Even with the development of some new and better measures, the academic publishing world too often defaults to the traditional measures based on citations, such as impact factor and citation number, that were used 60 years ago, in a time before preprint repositories and mega-journals existed and before academia became such a hypercompetitive environment. Most important, however, is that these measures have degenerated into becoming purely targets. Goodhart’s Law is clearly being illustrated: when a citation-based measure becomes the target, the measure itself ceases to be meaningful, useful, or accurate.

Our study’s extensive analysis of academic publications reveals why using citation-based metrics as measures of impact is wrong at the core: First, not all citations are equal; there is a big difference between a study that cites a paper that greatly influenced it and a study that cites multiple papers with only minor connections. Many of the impact measures widely used today do not take into consideration distinctions among the various types of citations. Second, it is not logical to measure a paper’s impact based on the citation numbers of other papers that are published in the same journal. In the academic world, there are >20,000 journals that publish hundreds or even thousands of papers each year, with papers written by hundreds or even thousands of authors. It is even less logical to measure a researcher’s impact based on a paper co-authored with many other researchers according to the journal in which it is published. Third, as we demonstrated in the section, it is wrong to compare studies from different fields, and even to compare papers and researchers within the same parent field of study, due to the many differences in the median and mean number of citations in each field (see Table 1).

As we have revealed in this study, to measure impact with citation-based measures—that have now become targets—clearly has many undesirable effects. The number of papers with limited impact has increased sharply (see Fig. S11), papers may contain hundreds of self-citations (see Fig. 7), and some top journals have become “old boys’ clubs” that mainly publish papers from the same researchers (see Figs 17 and 18). Moreover, using citation-based measures to compare researchers in different fields may have the dangerous effect of allocating more resources to high-citation domains, shortchanging other domains that are equally important.

We believe the solution to the aforementioned issues is to use data-science tools and release new and open datasets in order to promote using existing unbiased measures or to develop new measures that will more accurately determine a paper’s impact in a specific research field. Moreover, it is vital to raise awareness of the shortcomings of commonly used measures, such as the number of citations, h-index, and impact factor. Certain metrics have been proposed, but the key is to wisely and carefully evaluate new measures to ensure that they will not follow Goodhart’s Law and end up merely as targets. Researchers do valuable work. Communicating the work to others is vital, and correctly assessing the impact of that work is essential.

## Methods

To analyze the above MAG and AMiner large-scale datasets, we developed an open source framework written in Python, which provided an easy way to query the datasets. The framework uses TuriCreate’s SFrame dataframe objects [83] to perform big-data analysis on tens of millions of records to calculate how various properties have changed over time. For example, we used SFrame objects to analyze how the mean number of authors and title lengths evolved. However, while SFrame is exceptionally useful for calculating various statistics using all-papers features, it is less convenient and less computationally cost-effective for performing more complicated queries, such as calculating the mean age of the last authors in a certain journal in a specific year.

To perform more complex calculations, we loaded the datasets into the MongoDB database [84]. Next, we developed a code framework that easily let us obtain information on papers, authors, paper collections, venues, and research fields. The framework supports calculating complex features of the specified object in a straightforward manner. For example, with only a few and relatively simple lines of Python code, we were able to calculate the mean number of co-authors per author in a specific year for authors who started their career in a specific decade. An overview of our code framework is presented in Fig. S1.

To make our framework accessible to other researchers and to make this study completely reproducible, we have written Jupyter Notebook tutorials that demonstrate how the SFrame and MongoDB collections were constructed from the MAG, AMiner, and SJR datasets (see Availability of Source Code and Requirements section and RRID SCR_016958).

## Availability of supporting data and materials

An interactive web interface to explore the study’s data is available at the project’s website. The web interface provides researchers the ability to interactively explore and better understand how various journals’ properties have changed over time (see Fig. S36 and RRID: SCR_016958). Additionally, the website contains the Fields-of-Research Features data.

Supporting data and a copy of the code are available from the *GigaScience* GigaDB repository [85].

## Availability of source code and requirements

One of the main goals of this study was to create an open source framework, which provided an easy way to query the datasets. Our code framework, including tutorials, is available at the project’s website.

- Project name: Science Dynamics
- Project home page: http://sciencedynamics.data4good.io/
- Operating system(s): Platform independent
- Programming language: Python
- Other requirements: Python 2.7, MongoDB, TuriCreate Python Package
- License: MIT License
- RRID: SCR_016958

## Additional files

**Additional Figure S1:** Overview of the code framework. The datasets are loaded into SFrame objects and MongoDB collections. The SFrame objects are used mainly to obtain general insights by analyzing tens of millions of papers and author records. The MongoDB collections are used to construct Paper and Author objects that can be used to analyze more complicated statistics for specific venues and research fields with usually hundreds of thousands of records.

**Additional Figure S2:** Percentage of titles with question or exclamation marks. The percentage of papers with question or exclamation marks in their titles increased over time, as well as the percentage of titles with interrobangs (represented by ?! or !?).

**Additional Figure S3:** Mean number of authors over time. There has been an increase in the mean number of authors, especially in recent decades.

**Additional Figure S4:** Maximal number of authors over time. In recent years the maximal number of authors per paper increased sharply from 520 authors in 2000 to >3,100 authors in 2010.

**Additional Figure S5:** Percentage of author lists in alphabetical order. There has been a decline in the number of author lists organized in alphabetical order.

**Additional Figure S6:** Mean length of abstracts. Since 1970 there has been an increase in abstracts’ mean number of words.

**Additional Figure S7:** Keyword trends. Both the number of papers with keywords has increased, as well as the mean number of keywords per paper.

**Additional Figure S8:** Mean number of fields of study over time. Over time both the mean number of L0 and L1 fields of study per paper considerably increased. We believe the decrease in the mean number of L0 and L1 fields is a direct result of the decrease in the number of papers with keywords in the same years (see the Results of Paper Trends section).

**Additional Figure S9:** Mean number of references over time. Over time, the mean number of references sharply increased.

**Additional Figure S10:** Distributions over time of references in papers. Over time, papers with a relatively high number of references have become more common.

**Additional Figure S11:** Total number of papers with no citations after 5 years. The number of papers in this category increased sharply over time.

**Additional Figure S12:** Total number of self-citations and percentage of papers with self-citations. We can observe that over time both the total number of self-citations as well as the percentage of papers with self-citations increased significantly.

**Additional Figure S13:** Spearman correlation heat map for papers’ properties. We can observe positive correlations among papers’ various structural properties and the papers’ total number of citations after 5 years.

**Additional Figure S14:** New authors over time. The number of authors, with unique MAG author IDs, who published their first paper each year.

**Additional Figure S15:** Authors' mean number of conference and journal papers over time. The mean publication rate of both journal and conference papers increased with every decade.

**Additional Figure S16:** Authors’ median sequence number over time. We can see that over time the median sequence numbers increased; i.e., senior researchers tend to have higher sequence numbers.

**Additional Figure S17:** Number of journals over time according to the MAG dataset. There has been a drastic increase in the number of journals since the 1960s.

**Additional Figure S18:** Number of new journals by year. Hundreds of new ranked journals are being published each year.

**Additional Figure S19:** Journals’ h-index mean and median values. Over time both the mean and median values of the journals’ h-index measures decreased.

**Additional Figure S20:** SJR values over time. We can observe that over time both the mean and median SJR values increased.

**Additional Figure S21:** Top journals’ number of papers and authors over time. We can observe that both the number of papers and authors increased sharply in recent years.

**Additional Figure S22:** Top selected journals’ number of papers over time. In the vast majority of the selected journals the number of published papers with ≥5 references increased considerably over time.

**Additional Figure S23:** Top selected journals' mean author career age over time. In the vast majority of the selected journals, the mean age of authors, especially last authors, increased greatly over time.

**Additional Figure S24:** L0 Fields-of-study number of papers over time. We can observe the large diversity in the number of papers published in each L0 research field.

**Additional Figure S25:** L0 Fields-of-study mean authors number. We can observe a variation in the mean number of authors across the various research fields.

**Additional Figure S26:** L0 Fields-of-study mean references numbers. We can observe variance among the reference numbers in different fields.

**Additional Figure S27:** Biology L1-subfields number of papers over time. We can observe a big variance in the number of papers over time in the various biology subfields.

**Additional Figure S28:** Genetics L2-subfields number of papers over time. We can observe a big variance in the number of papers over time in the various genetics subfields.

**Additional Figure S29:** Biology L1-subfields mean number of authors over time. We can observe a variance in the mean number of authors over time in the various biology subfields.

**Additional Figure S30:** Genetics L3-subfields mean number of authors over time. We can observe a significant variance in the mean number of authors over time in the various genetics subfields.

**Additional Figure S31:** Biology L1-subfields mean number of references over time. We can observe a variance in the mean number of references over time in the various biology subfields.

**Additional Figure S32:** Genetics L2-subfields mean number of references over time. We can observe a significant variance in the mean number of references over time in the various genetics subfields.

**Additional Figure S33:** Biology L1-subfields median number of 5-year citations over time. We can observe a variance in the median number of citations over time in the various biology subfields.

**Additional Figure S34:** Genetics L2-subfields median number of 5-year citations over time. We can observe a significant variance in the median number of citations over time in the various genetics subfields.

**Additional Figure S35:** L3 Fields-of-study median 5-year citation distributions by parent fields. We can observe the high variance among the L3 fields-of-study median citation numbers.

**Additional Figure S36:** Interactive website. We have developed an interactive website at http://sciencedynamics.data4good.io/ that makes it possible to view and interact directly with the study’s data.

## Abbreviations

AWS: Amazon Web Services; DOI: Digital Object Identifier; ISSN: International Standard Serial Number; MAG: Microsoft Academic Graph; PNAS: Proceedings of the National Academy of Sciences of the United States of America; RePEc: Research Papers in Economics; SJR: SCImago Journal Rank.

## Competing interests

The authors declare that they have no competing interests.

## Authors' contributions

Both M.F. and C.G. conceived the concept of this study and developed the methodology. M.F. developed the study’s code and visualization and performed the data computational analysis. C.G. supervised the research.

## Supplementary Material

giz053_GIGA-D-18-00461_Original_Submission

giz053_GIGA-D-18-00461_Revision_1

giz053_GIGA-D-18-00461_Revision_2

giz053_GIGA-D-18-00461_Revision_3

giz053_Response_to_Reviewer_Comments_Original_Submission

giz053_Response_to_Reviewer_Comments_Revision_1

giz053_Response_to_Reviewer_Comments_Revision_2

giz053_Reviewer_1_Report_Original_Submission -- Jean-Noel Vergnes 12/7/2018 Reviewed

giz053_Reviewer_2_Report_Original_Submission -- Sven Hug 12/22/2018 Reviewed

giz053_Supplement_Figures

## References

[1] Ware M, Mabe M The STM report: An overview of scientific and scholarly journal publishing. 2015 https://www.stm-assoc.org/2015_02_20_STM_Report_2015.pdf. Accessed 12 May 2019.

[2] Herrmannova D, Knoth P An analysis of the Microsoft Academic Graph. D-Lib Mag. 2016;22(9/10), doi:10.1045/september2016-herrmannova.

[3] Björk BC Have the “mega-journals” reached the limits to growth?. PeerJ. 2015;3:e981. 26038735 10.7717/peerj.981 PMC4451030

[4] Kelly S The continuing evolution of publishing in the biological sciences. Biol Open. 2018;7(8), doi:10.1242/bio.037325. PMC6124566 30154108

[5] Roemer RC, Borchardt R From bibliometrics to altmetrics: A changing scholarly landscape. Coll Res Lib News. 2012;73(10):596–600.

[6] Hirsch JE An index to quantify an individual’s scientific research output. Proc Natl Acad Sci U S A. 2005;102(46):16569–72. 16275915 10.1073/pnas.0507655102 PMC1283832

[7] Garfield E The agony and the ecstasy—the history and meaning of the journal impact factor. 2005 http://garfield.library.upenn.edu/papers/jifchicago2005.pdf. 10.1001/jama.295.1.90 16391221

[8] Wilsdon J The Metric Tide: Independent Review of the Role of Metrics in Research Assessment and Management. Sage; 2015.

[9] Edwards MA, Roy S Academic research in the 21st century: Maintaining scientific integrity in a climate of perverse incentives and hypercompetition. Environ Eng Sci. 2017;34(1):51–61. 28115824 10.1089/ees.2016.0223 PMC5206685

[10] Biagioli M Watch out for cheats in citation game. Nat News. 2016;535(7611):201. 10.1038/535201a 27411599

[11] Campbell DT Assessing the impact of planned social change. Eval program plann. 1979;2(1):67–90.

[12] Newton AC Implications of Goodhart’s Law for monitoring global biodiversity loss. Conserv Lett. 2011;4(4):264–68.

[13] Mizen P Central Banking, Monetary Theory and Practice: Essays in honour of Charles Goodhart, vol. 1 Edward Elgar; 2003.

[14] Chrystal KA, Mizen PD, Mizen P Goodhart’s Law: Its origins, meaning and implications for monetary policy. .

[15] Francescani C NYPD report confirms manipulation of crime stats. Reuters. 2012 https://www.reuters.com/article/us-crime-newyork-statistics/nypd-report-confirms-manipulation-of-crime-stats-idUSBRE82818620120309 Accessed 12 May 2019.

[16] Kliger AS Quality measures for dialysis: Time for a balanced scorecard. Clini J Am Soc Nephrol. 2016;11(2):363–68. 10.2215/CJN.06010615 PMC4741039 26316622

[17] Šupak Smolčić V Salami publication: Definitions and examples. Biochem Med (Zagreb). 2013;23(3):237–41. 24266293 10.11613/BM.2013.030 PMC3900084

[18] Schofferman J, Wetzel FT, Bono C Ghost and guest authors: You can’t always trust who you read. Pain Med. 2015;16(3):416–20. 25338945 10.1111/pme.12579

[19] Head ML, Holman L, Lanfear R et al. The extent and consequences of p-hacking in science. PLoS Biol. 2015;13(3):e1002106. 25768323 10.1371/journal.pbio.1002106 PMC4359000

[20] Bartneck C, Kokkelmans S Detecting h-index manipulation through self-citation analysis. Scientometrics. 2010;87(1):85–98. 21472020 10.1007/s11192-010-0306-5 PMC3043246

[21] Kupferschmidt K Tide of lies. Science. 2018;361(6403):636–41. 30115791 10.1126/science.361.6403.636

[22] Haug CJ Peer-review fraud–hacking the scientific publication process. N Engl J Med. 2015;373(25):2393–95. 26488392 10.1056/NEJMp1512330

[23] Dansinger M Dear plagiarist: A letter to a peer reviewer who stole and published our manuscript as his own. Ann Intern Med. 2017;166(2):143–143. 27951597 10.7326/M16-2551

[24] Post A, Li AY, Dai JB, et al. c-index and subindices of the h-index: New variants of the h-index to account for variations in author contribution. Cureus. 2018;10(5):e2629. 30027021 10.7759/cureus.2629 PMC6044490

[25] Romanovsky AA Revised h index for biomedical research. Cell Cycle. 2012;11(22):4118–21. 22983124 10.4161/cc.22179 PMC3524206

[26] Wu Q The w-index: A measure to assess scientific impact by focusing on widely cited papers. J Am Soc Inf Sci Technol. 2010;61(3):609–14.

[27] Waltman L, van Eck NJ, van Leeuwen TN, et al. Towards a new crown indicator: An empirical analysis. Scientometrics. 2011;87(3):467–81. 21654898 10.1007/s11192-011-0354-5 PMC3081055

[28] Top publications. Google Scholar. https://scholar.google.com/citations?view_op=top_venues. Accessed 13 February 2019.

[29] Journal Citation Reports (JCR) https://jcr.clarivate.com/. Accessed 12 May 2019.

[30] Fortunato S, Bergstrom CT, Börner K, et al. Science of science. Science. 2018;359(6379):eaao0185. 29496846 10.1126/science.aao0185 PMC5949209

[31] arXiv Monthly Submission Rates https://arxiv.org/stats/monthly_submissions. Accessed 20 January 2019.

[32] Learn JR What bioRxiv’s first 30,000 preprints reveal about biologists. Nat News. 2019, doi:10.1038/d41586-019-00199-6.

[33] Davis P Scientific Reports overtakes PLoS One as largest megajournal. The Scholarly Kitchen https://scholarlykitchen.sspnet.org/2017/04/06/scientific-reports-overtakes-plos-one-as-largest-megajournal/. Accessed 9 July 2018.

[34] Cronin B Hyperauthorship: A postmodern perversion or evidence of a structural shift in scholarly communication practices?. J Am Soc Inf Sci Technol. 2001;52(7):58–69.

[35] Von Bergen C, Bressler MS Academe’s unspoken ethical dilemma: Author inflation in higher education. Res High Educ. 2017;32.

[36] Mallapaty S Paper authorship goes hyper author. 2018 Nature Index https://www.natureindex.com/news-blog/paper-authorship-goes-hyper.

[37] Abbott BP, Abbott R, Abbott T, et al. Observation of gravitational waves from a binary black hole merger. Phys Rev Lett. 2016;116(6):061102. 26918975 10.1103/PhysRevLett.116.061102

[38] Castelvecchi D LIGO’s unsung heroes. Nature News. 2017 https://www.nature.com/news/ligo-s-unsung-heroes-1.22786.

[39] Aboukhalil R The rising trend in authorship. The Winnower. 2014;2:e141832, doi:10.15200/winn.141832.26907.

[40] Wislar JS, Flanagin A, Fontanarosa PB, et al. Honorary and ghost authorship in high impact biomedical journals: A cross sectional survey. BMJ. 2011;343:d6128. 22028479 10.1136/bmj.d6128 PMC3202014

[41] Kennedy MS, Barnsteiner J, Daly J Honorary and ghost authorship in nursing publications. J Nurs Scholarsh. 2014;46(6):416–22. 24930670 10.1111/jnu.12093

[42] Vera-Badillo FE, Napoleone M, Krzyzanowska MK, et al. Honorary and ghost authorship in reports of randomised clinical trials in oncology. Eur J Cancer. 2016;66:1–8. 27500368 10.1016/j.ejca.2016.06.023

[43] Economist T Why research papers have so many authors. The Economist. 2016 http://www.economist.com/news/science-and-technology/21710792-scientific-publications-are-getting-more-and-more-names-attached-them-why. Accessed 12 May 2019.

[44] Lewison G, Hartley J What’s in a title? Numbers of words and the presence of colons. Scientometrics. 2005;63(2):341–56.

[45] Lockwood G Academic clickbait: Articles with positively-framed titles, interesting phrasing, and no wordplay get more attention online. The Winnower. 2016;3, doi:10.15200/winn.146723.36330.

[46] Ucar I, López-Fernandino F, Rodriguez-Ulibarri P, et al. Growth in the number of references in engineering journal papers during the 1972–2013 period. Scientometrics. 2014;98(3):1855–64.

[47] Gálvez A, Maqueda M, Martinez-Bueno M, et al. Scientific publication trends and the developing world: What can the volume and authorship of scientific articles tell us about scientific progress in various regions?. American Sci. 2000;88(6):526–33.

[48] Jagsi R, Guancial EA, Worobey CC, et al. The “gender gap” in authorship of academic medical literature–a 35-year perspective. N Engl J Med. 2006;355(3):281–87. 16855268 10.1056/NEJMsa053910

[49] Johnson MR, Wagner NJ, Reusch J Publication trends in top-tier journals in higher education. J Appl Res Higher Educ. 2016;8(4):439–54.

[50] Aldhous P Scientific publishing: the inside track. Nat News. 2014;510(7505):330. 10.1038/510330a 24943942

[51] Porter A, Rafols I Is science becoming more interdisciplinary? Measuring and mapping six research fields over time. Scientometrics. 2009;81(3):719–45.

[52] Fanelli D, Larivière V Researchers’ individual publication rate has not increased in a century. PLoS One. 2016;11(3):e0149504. 26960191 10.1371/journal.pone.0149504 PMC4784736

[53] Dong Y, Ma H, Shen Z, et al. A century of science: Globalization of scientific collaborations, citations, and innovations. In: Proceedings of the 23rd ACM SIGKDD International Conference on Knowledge Discovery and Data Mining ACM; 2017:1437–46.

[54] Yan E, Ding Y Weighted citation: An indicator of an article’s prestige. J Am Soc Inf Sci Technol. 2010;61(8):1635–43.

[55] Lehmann S, Jackson AD, Lautrup BE Measures for measures. Nature. 2006;444(7122):1003. 17183295 10.1038/4441003a

[56] Garfield E The meaning of the impact factor. Rev Int Psicol Clin Salud. 2003;3(2):363–9.

[57] Hirsch JE Does the h index have predictive power?. Proc Natl Acad Sci U S A. 2007;104(49):19193–8. 18040045 10.1073/pnas.0707962104 PMC2148266

[58] Fong EA, Wilhite AW Authorship and citation manipulation in academic research. PloS One. 2017;12(12):e0187394. 29211744 10.1371/journal.pone.0187394 PMC5718422

[59] The cost of salami slicing. Nat Mater. 2005;4, doi:10.1038/nmat1305.

[60] Delgado López-Cózar E, Robinson-García N, Torres-Salinas D The Google scholar experiment: How to index false papers and manipulate bibliometric indicators. J Assoc Inf Sci Technol. 2014;65(3):446–54.

[61] Van Bevern R, Komusiewicz C, Niedermeier R, et al. H-index manipulation by merging articles: Models, theory, and experiments. Artif Intell. 2016;240:19–35.

[62] Falagas ME, Kouranos VD, Arencibia-Jorge R, et al. Comparison of SCImago journal rank indicator with journal impact factor. FASEB J. 2008;22(8):2623–28. 18408168 10.1096/fj.08-107938

[63] Lariviere V, Kiermer V, MacCallum CJ, et al. A simple proposal for the publication of journal citation distributions. BioRxiv. 2016, doi:10.1101/062109.

[64] Callaway E Beat it, impact factor! Publishing elite turns against controversial metric. Nat News. 2016;535(7611):210. 10.1038/nature.2016.20224 27411614

[65] Altmetric https://www.altmetric.com/. Accessed 14 February 2019.

[66] Griffin SA, Oliver CW, Murray A Altmetrics! Can you afford to ignore it?. Br J Sports Med. 2017;52(18):1160–1. 28831013 10.1136/bjsports-2017-098258

[67] Semantic Scholar https://www.semanticscholar.org. Accessed 14 February 2019.

[68] Seglen PO Why the impact factor of journals should not be used for evaluating research. BMJ. 1997;314(7079):498. 9056804 10.1136/bmj.314.7079.497 PMC2126010

[69] Byrne A Comment: Measure for measure. Nature. 2017;546(7666):S22. 10.1038/548S22a 28792926

[70] Hecht F, Hecht BK, Sandberg AA The journal ”impact factor”: a misnamed, misleading, misused measure. Cancer Genetics Cytogenet. 1998;104(2):77–81. 10.1016/s0165-4608(97)00459-7 9666797

[71] Google Scholar https://scholar.google.com. Accessed 13 February 2019.

[72] Sinha A, Shen Z, Song Y, et al. An overview of Microsoft Academic Service (MAS) and applications. In: Proceedings of the 24th International Conference on World Wide Web ACM; 2015:243–46., doi:10.1145/2740908.2742839.

[73] KDD Cup 2016: Whose papers are accepted the most: towards measuring the impact of research institutions. 2016 http://www.kdd.org/kdd-cup/view/kdd-cup-2016/Data.

[74] Semantic Scholar - An Overview of Microsoft Academic Service (MAS) and Applications https://www.semanticscholar.org/paper/An-Overview-of-Microsoft-Academic-Service-(MAS)-and-Sinha-Shen/b6b6d2504fd57d27a0467654fa62169cc7dedbdd?navId=citing-papers. Accessed 14 February 2019.

[75] Pitts M, Savvana S, Roy SB, et al. ALIAS: Author Disambiguation in Microsoft Academic Search Engine Dataset. In: Proceedings of the 17th International Conference on Extending Database Technology (EDBT 2014); 2014, doi:10.5441/002/edbt.2014.65.

[76] How Microsoft Academic uses knowledge to address the problem of conflation/disambiguation 2018 https://www.microsoft.com/en-us/research/project/academic/articles/microsoft-academic-uses-knowledge-address-problem-conflation-disambiguation/. Accessed 21 January 2019.

[77] Tang J, Zhang J, Yao L, et al. ArnetMiner: extraction and mining of academic social networks. In: Proceedings of the 14th ACM SIGKDD International Conference on Knowledge Discovery and Data Mining, Las Vegas, NV, 2008. 2008, doi:10.1145/1401890.1402008.

[78] Butler D Free journal-ranking tool enters citation market. Nature. 2008;451:6. 18172465 10.1038/451006a

[79] Scimago Journal & Country Rank https://www.scimagojr.com/journalrank.php. Accessed 14 February 2019.

[80] Pycld2 - Python Package https://pypi.org/project/pycld2/. Accessed 14 February 2019.

[81] Colavizza G, Franssen T, van Leeuwen T An empirical investigation of the tribes and their territories: are research specialisms rural and urban?. J Informetr. 2019;13(1):105–17.

[82] *Nature*: Editorial Criteria and Processes https://www.nature.com/nature/for-authors/editorial-criteria-and-processes. Accessed 15 July 2018.

[83] Low Y, Gonzalez JE, Kyrola A, et al. Graphlab: A new framework for parallel machine learning. arXiv. 2014:1006.4990.

[84] MongoDB http://www.mongodb.com. Accessed 14 February 2019.

[85] Fire M, Guestrin C Supporting data for “Over-optimization of academic publishing metrics: observing Goodhart’s Law in action”. GigaScience Database. 2019 10.5524/100587. PMC6541803 31144712

