# Supplementary material for: Over-optimization of academic publishing metrics: observing Goodhart’s Law in action
Source: Gigascience. 2019 May 30;8(6):giz053. doi: 10.1093/gigascience/giz053 (PMC6541803; doi:10.1093/gigascience/giz053)
Supplement: giz053_GIGA-D-18-00461_Revision_2 [file giz053_giga-d-18-00461_revision_2.pdf]

GigaScience

Over-Optimization of Academic Publishing Metrics: Observing Goodhart's Law in Action

--Manuscript Draft--

**Manuscript Number:**	GIGA-D-18-00461R2	
**Full Title:**	Over-Optimization of Academic Publishing Metrics: Observing Goodhart's Law in Action	
**Article Type:**	Research	
**Funding Information:**	Washington Research Foundation Fund for Innovation in Data-Intensive Discovery (1)	Dr. Michael Fire
	Amazon Web Services (0)	Dr. Michael Fire
	Gordon and Betty Moore Foundation (0)	Dr. Michael Fire
	Alfred P. Sloan Foundation (0)	Dr. Michael Fire
	Microsoft Research (US) (0)	Dr. Michael Fire
**Abstract:**	The academic publishing world is changing significantly, with ever-growing numbers of publications each year and shifting publishing patterns. However, the metrics used to measure academic success, such as the number of publications, citation number, and impact factor, have not changed for decades. Moreover, recent studies indicate that these metrics have become targets and follow Goodhart's Law, according to which ``when a measure becomes a target, it ceases to be a good measure.'' In this study, we analyzed over 120 million papers to examine how the academic publishing world has evolved over the last century, with a deeper look into the specific field of biology. Our study shows that the validity of citation-based measures is being compromised and their usefulness is lessening. In particular, the number of publications has ceased to be a good metric as a result of longer author lists, shorter papers, and surging publication numbers. Citation-based metrics, such citation number and h-index, are likewise affected by the flood of papers, self-citations, and lengthy reference lists. Measures such as a journal's impact factor have also ceased to be good metrics due to the soaring numbers of papers that are published in top journals, particularly from the same pool of authors. Moreover, by analyzing properties of over 2600 research fields, we observed that citation-based metrics are not beneficial for comparing researchers in different fields, or even in the same department. Academic publishing has changed considerably; now we need to reconsider how we measure success.	
**Corresponding Author:**	Michael Fire, Ph.D<br>BGU<br>Beer-Sheva, Israel ISRAEL	
**Corresponding Author Secondary Information:**		
**Corresponding Author's Institution:**	BGU	
**Corresponding Author's Secondary Institution:**		
**First Author:**	Michael Fire, Ph.D	
**First Author Secondary Information:**		
**Order of Authors:**	Michael Fire, Ph.D	
	Carlos Guestrin	
**Order of Authors Secondary Information:**		
**Response to Reviewers:**	Please find the response letter attached	

Powered by Editorial Manager® and ProduXion Manager® from Aries Systems Corporation

Additional Information:	
**Question**	**Response**
Are you submitting this manuscript to a special series or article collection?	No
**Experimental design and statistics**<br><br>Full details of the experimental design and statistical methods used should be given in the Methods section, as detailed in our Minimum Standards Reporting Checklist. Information essential to interpreting the data presented should be made available in the figure legends.<br><br>Have you included all the information requested in your manuscript?	Yes
**Resources**<br><br>A description of all resources used, including antibodies, cell lines, animals and software tools, with enough information to allow them to be uniquely identified, should be included in the Methods section. Authors are strongly encouraged to cite Research Resource Identifiers (RRIDs) for antibodies, model organisms and tools, where possible.<br><br>Have you included the information requested as detailed in our Minimum Standards Reporting Checklist?	Yes
**Availability of data and materials**<br><br>All datasets and code on which the conclusions of the paper rely must be either included in your submission or deposited in publicly available repositories (where available and ethically appropriate), referencing such data using a unique identifier in the references and in the "Availability of Data and Materials" section of your manuscript.	Yes

Have you have met the above requirement as detailed in our Minimum Standards Reporting Checklist?	

Powered by Editorial Manager® and ProduXion Manager® from Aries Systems Corporation

Manuscript
Click here to view linked References
Click here to access/download;Manuscript;fire_guestrion- final-minor.pdf

OXFORD
(GIGA)n SCIENCE

GigaScience, 2017, 1–45

doi: xx.xxxx/xxxx
Manuscript in Preparation
Paper

PAPER

Over-Optimization of Academic Publishing Metrics: Observing Goodhart's Law in Action

Michael Fire[1,*] and Carlos Guestrin[2,†]

[1]Software and Information Systems Engineering Department, Ben-Gurion University and [2]Paul G. Allen School of Computer Science & Engineering, University of Washington

*mickyfi@bgu.ac.il
†guestrin@cs.washington.edu

Abstract

The academic publishing world is changing significantly, with ever-growing numbers of publications each year and shifting publishing patterns. However, the metrics used to measure academic success, such as the number of publications, citation number, and impact factor, have not changed for decades. Moreover, recent studies indicate that these metrics have become targets and follow Goodhart's Law, according to which "when a measure becomes a target, it ceases to be a good measure." In this study, we analyzed over 120 million papers to examine how the academic publishing world has evolved over the last century, with a deeper look into the specific field of biology. Our study shows that the validity of citation-based measures is being compromised and their usefulness is lessening. In particular, the number of publications has ceased to be a good metric as a result of longer author lists, shorter papers, and surging publication numbers. Citation-based metrics, such citation number and h-index, are likewise affected by the flood of papers, self-citations, and lengthy reference lists. Measures such as a journal's impact factor have also ceased to be good metrics due to the soaring numbers of papers that are published in top journals, particularly from the same pool of authors. Moreover, by analyzing properties of over 2600 research fields, we observed that citation-based metrics are not beneficial for comparing researchers in different fields, or even in the same department. Academic publishing has changed considerably; now we need to reconsider how we measure success.

Key words: Science of Science; Scientometrics; Goodhart's Law; Data Science; Big Data; Academic Publishing Metrics

Introduction

In the last century, the academic publishing world has changed drastically in volume and velocity [1]. The volume of papers has increased sharply from less than a million papers published in 1980 to over 7 million papers published in 2014 [2]. Furthermore, the speed in which researchers can share and publish their studies has increased significantly. Today's researchers can publish not only in an ever-growing number of traditional venues, such as conferences and journals, but also in electronic preprint repositories and in mega-journals that provide rapid publication times [1, 3].

Along with the exponential increase in the quantity of published papers, the number of ranked scientific journals has increased to over 34,000 active peer-reviewed journals in 2014 [1], and the number of published researchers has soared [4]. As part of this escalation, metrics such as the number of papers, number of citations, impact factor, h-index, and altmetrics are being used to compare the impact of papers, researchers, journals, and universities [5, 6, 7, 8]. Using quantitative metrics to rank researchers contributes to a hypercompetitive research environment, which is changing academic culture - and not in a positive direction [9].

Studies suggest that publication patterns have changed as a result of Goodhart's Law, according to which, "When a measure becomes a target, it ceases to be a good measure" [9, 10]. Goodhart's Law, and its closely related Campbell's Law [11], influence many systems in our everyday life, including edu-

Compiled on: February 25, 2019.
Draft manuscript prepared by the author.

cational [11], biological [12], and other decision-making systems [13, 14]. As an example, Goodhart's Law can be found in the NYPD's manipulation of crime reports (the "measure") in order to improve crime statistics (the "target") [15]. Another example is found in the educational system, revealing that when "test scores become the goal of the teaching process, they both lose their value as indicators of educational status and distort the educational process in undesirable ways" [11]. One more example can be found in the field of medicine, where the National Health Service in England sets incentives (pay for performance) for primary care doctors to improve the quality of care. Indeed, "they found the measures improved for diabetes and asthma care in the first years of the program. These improvements were on the basis of care reported in the medical records but not necessarily on care provided. The main effect of this pay-for-performance program may be to promote better recording of care rather than better care." [16]

Recent studies indicate that when measures become targets in academic publishing, the effectiveness of the measures can be compromised, and unwelcome and unethical behaviors may develop, such as salami publications [17], ghost authorships [18], p-hacking [19], metrics manipulation [20], faking research data [21], faking of peer reviews [22], and even plagiarizing by a peer reviewer [23].

If the influence of Goodhart's Law on academia is indeed significant, then it should be possible to observe that academic entities, such as researchers and journals, will over-optimize their own measures to achieve a desired target. Similar to the consequences of making test scores a target, chasing after certain measures in the academic publishing world to desperately win the battle of "*impact or perish*" [10] can have undesirable effects.

Certainly, newer academic publishing metrics have emerged that are more unbiased [20, 24, 25, 26, 27], and these may thwart the trend of measures becoming targets. Yet, the traditional metrics retain a strong hold on the overall academic system, and they are still vastly used for ranking purposes [28, 29].

In this study, our main goal was to utilize new advances in data science tools to perform an in-depth and precise bottom-up analysis of academic publishing over the decades. Our comprehensive analysis ranged from micro to macro levels as we studied individual researchers' behaviors as well as behavioral changes within large research domains. Additionally, we wanted to uncover how and if Goodhart's Law has changed academic publishing, with an in-depth look at trends within biology and genetics.

Our study was greatly influenced by a recent study by Edwards and Roy [9], who observed that academia has become a hypercompetitive environment that can lead to unethical behaviors. The driving force behind such behaviors is to manipulate the metrics that measure the research's impact solely to increase the quantitative measures (and hence the status) of the research.

To achieve our research goals, we developed an open-source code framework to analyze data from several large-scale datasets containing over 120 million publications, with 528 million references and 35 million authors,[1] since the beginning of the 19th century. This provided a precise and full picture of how the academic publishing world has evolved.

The objective of our study was to use this huge quantity of data to examine the validity of commonly used citation-based metrics for academic publishing. Specifically, we wanted to see if Goodhart's Law was applicable: *Are researchers focusing too much on simply attaining certain target metrics at the expense of high-quality, relevant research?*

The remainder of the paper is organized as follows: In the Background section, we provide an overview of related studies. In the Data Description section, we present the datasets used in this study, and in the Analyses section, we describe the algorithms and experiments used to analyze the study's data. In the Results, Discussion , and Conclusions sections, we present and discuss our results and offer our conclusions from this study.

Background

This research is a large-scale scientometrics study (also referred to as the "science of science" [30]). Scientometrics is the study of quantitative features and characteristics of scientific research. In this section, we present studies that analyze changes in academic publications in recent years (see the Changes in Publication Trends section), and we provide an overview of common metrics that measure the impact of published papers (see the Success Metrics and Citation Trends section).

Changes in Publication Trends

One prevalent and increasing trend is to publish papers in preprint repositories, such as arXiv, bioRxiv, Research Papers in Economics (RePEc), and PubMed Central (PMC) [1]. For example, the use of arXiv surged from 4,275 submitted papers in September 2006 to 11,973 papers in November 2018 [31]. Additionally, over a million papers are now downloaded from bioRxiv every month [32]. Another common trend is to publish papers in mega-journals, such as PLOS ONE and Nature's Scientific Reports. Mega-journals are a new type of scientific journal that publishes peer-reviewed, open-access articles, where the articles have been reviewed for scientific trustworthiness, but not for scientific merit. Mega-journals accelerate review and publication times to 3-5 months and usually have high acceptance rates of over 50% [3]. In the first quarter of 2017, over 11,000 papers were published in PLOS ONE and Scientific Reports [33].

Another observable trend is that more and more papers are written by hundreds or even thousands of authors. This phenomena is known as hyperauthorship [34] or author inflation [35] and is common across research fields, where the majority of papers with over a thousand authors are produced in the physical sciences [36]. For example, the recent Laser Interferometer Gravitational-Wave Observatory (LIGO) paper [37] listed over 1000 authors [38]. Robert Aboukhalil measured this trend [39] and discovered that the average number of authors of academic papers has increased sharply since the beginning of the 20th century. Recently, Steven Kelly observed an unexpected increase in the average number of authors of papers in the biological sciences [4].

While papers' average number of authors has gone up over time, not all the authors have significantly contributed to the paper. In addition, honorary and ghost authors are prevalent. Wislar et al. found such evidence in biomedical journals [40], and similar findings were observed by Kennedy et al. [41] and by Vera-Badillo et al. [42]. The Economist recently published an article titled "Why research papers have so many authors" [43].

Lewison and Hartley [44] analyzed how papers' titles have changed over time. They discovered that titles' lengths have been increasing, along with the percentage of titles contain-

1 The number of authors was estimated according to the unique full names in the Microsoft Academic Graph dataset (see Results of Author Trends section)

ing colons. Additionally, Gwilym Lockwood observed that "articles with positively-framed titles, interesting phrasing, and no wordplay get more attention online" [45].

In addition to paper title lengths increasing, Ucar et al. have found lengthening reference lists for engineering journal papers, such as papers published in Biomedical Engineering and Information Theory [46].

Additionally, many studies have focused on how publication trends have changed over time, often focusing on specific geographical areas, various demographic characteristics, specific research domains, or specific journals. For example, Gálvez et al. [47] utilized the Science Citation Index to understand publication patterns in the developing world. Jagsi et al. [48] studied the gender gap in authorship of academic medical literature over 35 years. They discovered that the percentage of first and last authors who were women increased from 5.9% and 3.7% in 1970 to 29.3% and 19.3%, respectively, in 2004. Johnson et al. [49] studied publication trends in top-tier journals of higher education. Peter Aldhous analyzed publications in the National Academy of Sciences (PNAS) journal, to consider the influence of an "old boys' club" mentality [50]. In 2009, Porter and Rafols [51] used bibliometric indicators alongside a new index of interdisciplinarity to measure how the degree of interdisciplinarity has changed between 1975 and 2005 for six research domains. Porter and Rafols' findings suggest that "science is indeed becoming more interdisciplinary, but in small steps."

In 2016, Fanelli and Lariviè [52] analyzed the publication patterns of over 40,000 researchers for over a century. They observed that for researchers in their early career, both the total number of papers and the average number of collaborators increased over time. Fanelli and Larivière also observed that when adjusting the publication rate to account for co-authorship, then "the publication rate of scientists in all disciplines has not increased overall, and has actually mostly declined" [52]. In 2017, Dong et al. [53] utilized a dataset consisting of 89 million publications to study the evolution of scientific development over the past century. In their study, Dong et al. examined trends in *collaborations*, *citations*, and *impact*. From the collaboration perspective, Dong et al. observed that "the average length of a publication's author list tripled between 1900 and 2015, suggesting an increasingly collaborative scientific process." From analyzing citations patterns, they observed a sharp increase in the number of references over time, where in recent years, on average, papers reference 30 other papers. From the perspective of impact and innovations, Dong et al. observed "diversification of scientific development across the planet over the past century" [53]. While both our study and Dong et al.'s study utilize the Microsoft Academic Graph dataset, Dong et al. focused on the advancement of science and the globalization of scientific collaborations, citations, and innovations. Our study's primary goal was to perform an in-depth analysis of how the world of academic publishing has evolved over the decades. Moreover, we used additional large-scale datasets (see the Data Description section) to fully examine how academic publishing has evolved, investigating both micro trends (trends in the structure of papers) and macro trends (trends within research fields).

Success Metrics and Citation Trends

Over the years, various metrics have been proposed to measure papers, journal importance, and authors' impact. One of the most straightforward and commonly utilized measure is to simply count the researcher's number of publications. Another common metric is the citation number, either of a particular paper or the total citations received by all the author's papers. However, not all citations are equal [54]. Moreover, different research fields have different citation metrics, and therefore comparing them creates a problem: "The purpose of comparing citation records is to discriminate between scientists" [55].

One of the best-known and most-used measures to evaluate journals' importance is the impact factor, devised over 60 years ago by Eugene Garfield [7]. The impact factor measures the frequency in which an average article in a journal has been cited in a specific year. Over time, the measure has been used to "evaluate institutions, scientific research, entire journals, and individual articles" [56]. Another common metric to measure a researcher's output or a journal's impact is the h-index, which measures an author's or a journal's number of papers that have at least h citations each [6]. It has been shown that the h-index can predict academic achievements [57].

The above measures have been the standard for measuring academic publishing success. According to recent studies, and following Goodhart's Law, these metrics have now become targets, ripe for manipulation [9, 10, 58]. All types of manipulative methods are used, such as increasing the number of self-citations [20], increasing the number of publications by slicing studies into the smallest measurable quantum acceptable for publication [59], indexing false papers [60], and merging papers on Google Scholar [61]. Indeed, a recent study by Fong and Wilhite [58], which utilized data from over 12,000 responses to a series of surveys sent to more than 110,000 scholars from eighteen different disciplines, discovered "widespread misattribution in publications and in research proposals." Fong and Wilhite's findings revealed that the majority of researchers disapprove of this type of metric manipulation, yet many feel pressured to participate; other researchers blandly state "that it is just the way the game is played" [58].

While many of the above measures are easy to compute, they fail to consider the added contribution that is generally provided by the first and last authors. This issue becomes more cardinal with a sharply increasing number of papers with hundreds of coauthors. For example, "the h-index does not work well in the field of life sciences, where an author's position on a paper typically depends on the author's contribution" [25]. To tackle this issue, various measures such as the c-index [24] and revised h-index [25], have been suggested. These measures give higher weights to authors according to the coauthor order.

To overcome other shortcomings of commonly used measures, other alternative measures have been suggested. For example, the q-index [20] and w-index [26] are alternatives to the h-index. Likewise, the SJR indicator [62] and simple citation distributions [63] are offered as alternatives to the impact factor. Additional measures that normalize citation-based indicators using a paper's field of study and year of publication have also been suggested, and these are being used by several institutions [27].

Senior employees at several leading science publishers called upon journals to restrain from using the impact factor and suggested replacing it with simple citation distributions [63, 64]. Similarly, the altmetric [65] was proposed as an alternative metric to the impact factor and h-index. The altmetric [66] is a generalization of article-level metrics and considers other aspects of the impact of the work, such as the number of downloads, article views, mentions in social media, and more. The altmetric measure has gained in popularity in recent years, and several large publishers have started providing this metric to their readers. Additionally, Semantic Scholar [67] offers various measures to judge papers and researchers' influence. A thorough report regarding potential uses and limitations of metrics was written by Wilsdon et al. [8]. Additionally, an overview of the changing scholarly landscape can be found in Roemer and Borchardt's study [5].

Even with their many known shortcomings [8, 55, 24, 68,

69, 70], measures such as the impact factor, citation number, and h-index are still widely used. For example, the Journal Citation Reports publishes annual rankings based on journals' impact factors, and it continues to be widely followed [29]. As another example, the widely used Google Scholar web search engine [71] calculates the h-index and total number of citations of researchers, as well as journals' h-index, to rank journals and conferences [28].

Data Description

The Microsoft Academic Graph (MAG) Dataset

In this study we primarily utilized the Microsoft Academic Graph (MAG) [72], which was released as part of the 2016 KDD Cup [73]. The large-scale MAG dataset contains scientific publication records of over 120 million papers, along with citation relationships among those publications as well as relationships among authors, institutions, journals, conferences, and fields of study. In addition, the MAG dataset contains every author's sequence number for each paper's authors list. Furthermore, the dataset contains links between a publication and the one or more fields of study it belongs to. The fields of study are organized in hierarchical rankings with four levels, L0 to L3, where L0 is the highest level, such as a research field of computer science, and L3 is the lowest level, such as a research field of decision tree [2, 73]. Since its publication, the MAG dataset has gained an increasing popularity among scholars who utilize the dataset for scientometric studies [74]. An in-depth overview of the MAG dataset properties was presented by Herrmannova and Knoth [2]. According to their analysis of the MAG dataset, the five top fields of study – based on the number of papers – are physics, computer science, engineering, chemistry, and biology, with the number of papers ranging from slightly below 15 million in biology to over 20 million in physics [2].

Even though the MAG dataset contains papers that were published through 2016, we wanted to use years in which the data was the most comprehensive, so we focused our analysis on 120.7 million papers which were published through the end of 2014. Furthermore, we noted that the dataset contains many papers that are news items, response letters, comments, etc. Even though these items are important, they can affect a correct understanding of the underlying trends in scientific publications. Therefore, we focused our research on a dataset subset, which consists of over 22 million papers. This subset contains only papers which have a Digital Object Identifier (DOI) and at least 5 references. Additionally, while calculating various authors' properties, we primarily considered only the 22.4 million authors with unique author ID values in the selected papers' subset.[2]

The AMiner Dataset

The AMiner open academic graph dataset [77] contains data from over 154 million papers. The dataset contains various papers' attributes, such as titles, keywords, abstracts, venues, languages, and ISSNs. In our study, we primarily utilized the AMiner dataset to analyze papers' abstracts, to estimate papers' lengths, and to compare results with those obtained using the MAG dataset in order to validate the existence of observed patterns in both datasets. The AMiner is a relatively new dataset, and we are among the first to use it for a scientometric study.

2 Identifying all the papers by the same author (also known as author disambiguation [75]) is a challenging task. The MAG dataset provides a unique author ID for names that were matched to be the same individual. Recently, Microsoft Academic published a post titled "How Microsoft Academic uses knowledge to address the problem of conflation/disambiguation," which explains how Microsoft Academic performs author disambiguation [76].

The SCImago Journal Rank Dataset

To better understand trends in journal publications, we used the SCImago Journal Ranking (SJR) open dataset [78, 79]. This dataset contains details of over 23,000 journals with unique names between 1999 and 2016. For each journal, the SJR dataset contains the journal's SJR value, the number of published papers, the h-index, and the number of citations in each year. Additionally, the SJR dataset contains the best quartile, ranked from Q1 to Q4, of each journal. Journal quartiles are determined by the value of the boundary at the 25th, 50th, and 75th percentiles of an ordered distribution of the SJR indicator. Then, journals ranked Q1, Q2, Q3, and Q4 reflect the top 25%, between 25%-50%, between 50%-75%, and the bottom 25% of the distribution of the SJR indicator, respectively. The quartile rank is typically used to compare and rank journals within a given subject category.

The Join Dataset

To match the MAG journal IDs with their correlated various ranking measures, such as h-index and SJR, we joined all three datasets in the following manner: First, we joined the MAG and AMiner datasets by matching unique DOI values. Then, we matched ISSN values between the MAG-AMiner joined dataset with the SJR dataset.

Analyses

Analysis of Publication Trends

We used our developed code framework (see the Methods section) to explore how papers, authors, journals, and research fields have evolved over time. In the following subsections, we describe the specific calculations that were performed. Moreover, our Supplementary Materials section includes the precise code implementations which were used to obtain most of our results and to create the figures presented throughout this study.

Paper Trends

To explore how the quantity and structure of academic papers have changed over time, we performed the following: First, we calculated how many papers were published in the MAG dataset every year. Then, we utilized the pycld2 package [80] to detect the language of each paper's title and calculated the number of papers in each language. Next, we calculated the following paper features over time:

- Average number of words in titles and average number of characters per word (for papers with English titles)
- Percentage of titles that used question or exclamation marks (for papers with English titles)
- Average number of authors
- Percentage of papers in which authors appear in alphabetical order
- Average number of words in abstracts
- Average number of keywords
- Average number of references
- Length of papers

In addition, we utilized the papers with existing field-of-research values, matching the papers to their corresponding fields in order to identify each paper's top level (L0) research field. Using the top-level data, we were able to estimate the number of multidisciplinary papers that had more than one L0 research field. Afterwards, we calculated the percentage and total number of papers with no citations after 5 years, as well as the overall percentage of papers with self-citations over time.[3] Lastly, to better understand how citation patterns have changed across generations, we calculated the citation distributions after 10 years for each decade between 1950 and 2000.

Additionally, we selected all the papers in the Join dataset that had valid features[4] and were published between 1990 and 2009. Using the selected papers, we calculated the Spearman correlations among the title lengths, author numbers, reference numbers, overall lengths, and number of citations after 5 years. The results of the above described calculations are presented in the Results of Paper Trends section. Moreover, the code implementation is provided in the "Part III - A: Analyzing Changing Trends in Academia - Paper Trends" Jupyter Notebook (see the Availability of Source Code and Requirements section).

Author Trends

To study how authors' behaviors and characteristics have changed, we performed the following: First, we calculated how the number of new authors has changed over time. Second, for all authors who published their first paper after 1950, we divided the authors into groups according to each author's academic birth decade, i.e., the decade in which an author published his or her first paper. Next, for each group of authors with the same academic birth decade, we analyzed the following features:

- Average number of papers the authors in each group published n years after they began their careers, for $\forall n \in [0, 30]$. We performed these group calculations taking into account all papers, as well as only papers with at least 5 references
- Average number of conference and journal papers each group published n years after they began their careers, for $\forall n \in [0, 30]$
- Average number of coauthors each group had n years after they began their careers, for $\forall n \in [0, 30]$
- Authors' median sequence number each group had n years after they began their careers, for $\forall n \in [0, 60]$. Additionally, we calculated the average percentage of times the authors in each group were first authors

The results of the above described calculations are presented in the Results of Author Trends section. Moreover, the code implementation is provided in the "Part III - B: Analyzing Changing Trends in Academia - Author Trends" Jupyter Notebook (see the Availability of Source Code and Requirements section).

Journal Trends

To investigate how journal publication trends have changed over time, we used the SJR dataset to calculate the following features between 1999 and 2016:

- Number of journals with unique journal IDs that were active in each year
- Number of new journals that were published each year
- Average and maximal number of papers in each journal

Additionally, we utilized the SJR dataset to calculate how the journals' best quartile, average h-index, average SJR, and average citation number ($\frac{CitationNumber}{DocumentsNumber}$ (2 years)) metrics changed between 1999 and 2016.

Furthermore, we selected the 40 journals with the highest SJR values in 2016 and matched them to their corresponding journal IDs in the MAG dataset by matching each journal's ISSN and exact name in the MAG-AMiner joined dataset.[5] Using this method, we identified 30 unique journal IDs in the MAG dataset that published 110,825 papers with over 5 references. Then, for the matching journal IDs, we calculated the following features over time, for all papers that were published in the selected top journals:

- First and last authors' average career age
- Percentage of papers in which the first author had previously published in the one of the top journals
- Percentage of papers in which the last author had previously published in the one of the top journals

The results of the above described calculations are presented in Results of Journal Trends section. Moreover, the code implementation is provided in the "Part III - C: Analyzing Changing Trends in Academia - Journal Trends" Jupyter Notebook (see the Availability of Source Code and Requirements section).

Additionally, for over 8,400 journals with at least 100 published papers with 5 references, we calculated the following features over time:

- Number of papers
- Number of authors
- Top keywords in a specific year
- First/last/all authors average or median academic age
- Average length of papers
- Percentage of returning first/last/all authors, i.e., those who had published at least one prior paper in the journal

We developed a website with an interactive interface, which visualizes how the above features changed for each journal (see the Availability of Supporting Data section).

Field-of-Research Trends

We utilized the MAG dataset field-of-study values and the hierarchical relationship between various fields to match papers to their research fields in various levels (L0-L3). Then, for each field of study in its highest hierarchical level (L0), we calculated the following features over time: number of papers, number of authors, number of references, and average number of citations after five years. Next, we focused on the field of biology, which is in the L0 level. For all the L1 subfields of biology, we repeated the same feature calculations as in the previous step. Afterwards, we focused on genetics. For all the L2 subfields of genetics, we repeated the same feature calculations as in the previous step.

Additionally, to better understand the differences in citation patterns of various fields of research, we performed the following: For each field of study with at least 100 papers published in 2009, we calculated the following features using only papers that were published in 2009 and had at least 5 references:

- Number of papers
- Number of authors
- Median and average number of citations after 5 years

3 We define paper A as self-citing paper B if at least one of the authors of A is also an author of B.

4 We selected only papers having English titles and abstracts, existing author lists, references, and valid lengths. Additionally, we checked if the paper's title contained question or exclamation marks.

5 The top journal name was compared to the journal's name in the MAG dataset.

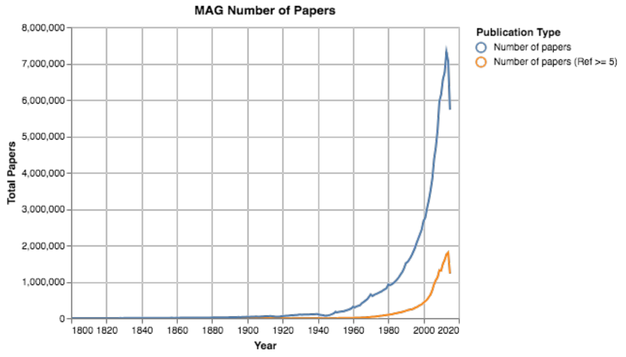

Figure 1. The Number of Papers over Time. The total number of papers has surged exponentially over the years.

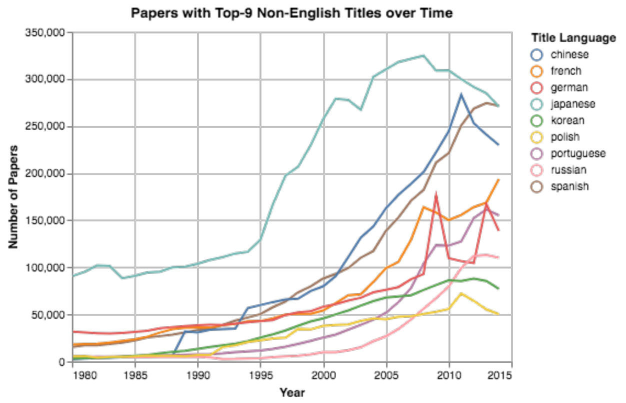

Figure 2. Papers with Top-9 Non-English Titles. Increasingly, more papers have non-English titles.

- Maximal number of citations after 5 years

The full features of over 2600 L3 fields of study are presented in Table 1.

The results of the above described calculations are presented in the Results of Fields-of-Research Trends section. Moreover, the code implementation is provided in the "Part III - D: Analyzing Changing Trends in Academia - Research Fields" Jupyter Notebook (see the Availability of Source Code and Requirements section).

Results

In the following subsections, we present all the results for the experiments which were described in the Analysis of Publication Trends section. Additional results are presented in the Supplementary Materials.

Results of Paper Trends

In recent years there has been a surge in the number of published academic papers, with over 7 million new papers each year and over 1.8 million papers with at least 5 references (see Figure 1).[6] Additionally, by analyzing the language of the papers' titles, we observed a growth in papers with non-English titles (see Figure 2).

As described in the Paper Trends section, we analyzed how various properties of academic papers have changed over time to better understand how papers' structures have evolved. In this analysis, we discovered that papers' titles became longer, from an average of 8.71 words in 1900 to an average of 11.83 words in 2014 (see Figure 3). Moreover, the average number of characters per word increased from 5.95 characters per average title word in 1900 to 6.6 characters per average title word in 2014 (see Figure 3). Additionally, we observed that in recent years the percentage of papers with question or exclamation marks increased sharply, from less than 1% of all papers in 1950 to over 3% of all papers in 2013 (see Figure S2). Furthermore, the usage of interrobangs (represented by ?! or !?) also increased sharply, from 0.0005% in 1950 to 0.0037% in 2013 (see Figure S2).

We explored how the number and order of the authors list has changed over time. The number of authors for papers with at least 5 references more than tripled over the years, from an average of 1.41 authors to an average of 4.51 authors per paper between 1900 and 2014, respectively (see Figure S3). Also, the maximal number of authors for a single paper in each year increased sharply over time, especially in recent years (see Figure S4). In fact, some recent papers actually listed over 3000 authors. Moreover, we observed that the percentage of author lists ordered alphabetically decreased in recent years, from 43.5% of all papers published in 1950 to 21% of all papers published in 2014 (see Figure S5). Furthermore, we discovered that with a higher number of authors, it is less likely that the authors list will be ordered alphabetically (see Figure 4). For example, in 2014 only about 1% of papers with six authors were ordered alphabetically.

When calculating how the abstracts of papers have changed over time, we discovered that the abstract length increased from an average of 116.3 words in 1970 to an average of 179.8 words in 2014 (see Figure S6). Moreover, with each decade since 1950, the distributions shifted to the right, showing that papers with longer abstracts of 400 and even 500 words have become more common over time (see Figure 5). Additionally, we analyzed how the number of keywords in papers has changed. We discovered that both the number of papers containing keywords increased, as well as the average number of keywords per paper (see Figure S7).

By estimating the percentage and number of multidisciplinary papers over time, we discovered an increase in the number of multidisciplinary papers until 2010, followed by a sharp decrease (see Figures 6 and S8). After performing further analysis, we believe the decline in the number of multidisciplinary papers is a result of papers with missing keywords in the MAG dataset, such as papers that were published in the PLOS ONE journal. These papers have dynamically changing keywords in the online version, but not in the offline version.

By examining how the number of references has changed over time, we observed a sharp increase in the average number of references per paper (see Figure S9). In addition, by analyzing the reference number distributions grouped by publishing decade, we can observe that higher numbers of references have become increasingly common. For example, in 1960 few papers had over 20 references, but by 2010 many papers had over 20 references, and some over 40 references (see Figure S10).

We also examined how self-citation trends have changed, and we observed that both the total number of self-citations and the percentage of papers with self-citations increased significantly (see Figure S12). Also, the average number of self-citations per paper, as well as the maximal number of self-citations in each year, increased sharply (see Figure 7). For example, about 3.67% of all papers in 1950 contained at least one self-citation, while 8.29% contained self-citations in 2014 (see Figure S12). Moreover, the maximal number of self-citations in a single paper increased sharply from 10 self-citations in a

6 There is a decline in the number of papers after 2014, probably due to missing papers in the MAG dataset, which was released in 2016.

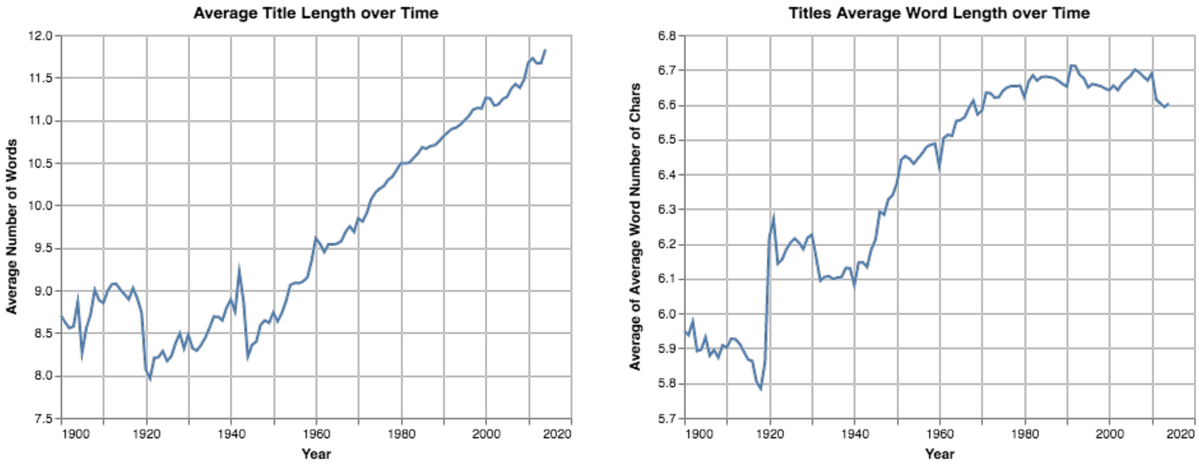

Figure 3. Average Title Length over Time. A paper's average title length increased from 8.71 words to over 11.83 words. Moreover, the average word length increased from 5.95 characters to 6.6 characters per title word.

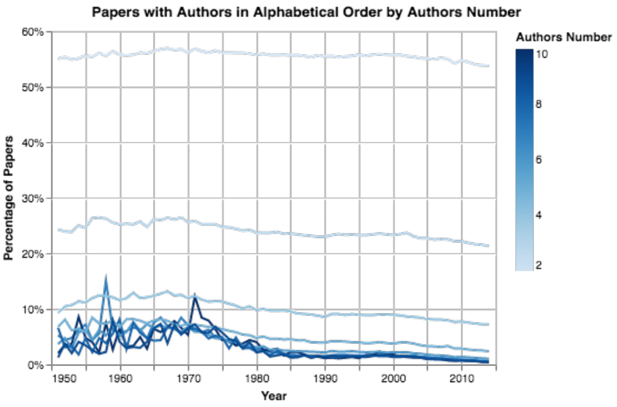

Figure 4. Percentage of Papers with Author Lists in Alphabetical Order, Grouped by the Number of Authors. The higher the number of authors, the less likely the authors will be organized alphabetically.

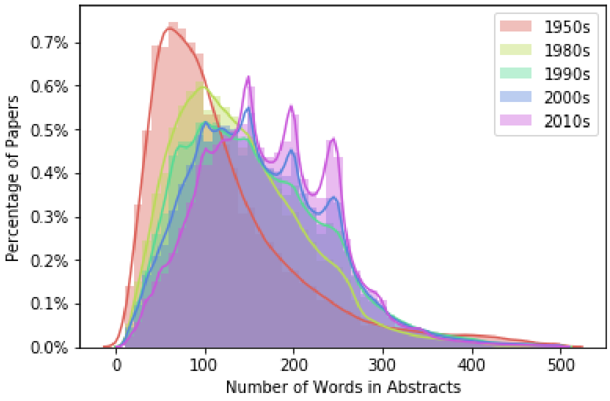

Figure 5. Distribution over Time of the Number of Words in Abstracts. Over time, papers' abstracts have tended to become longer.

paper published in 1950 to over 250 self-citations in a paper published in 2013 (see Figure 7).

By using the AMiner dataset to analyze how papers' lengths have changed, we discovered that the average and median length of papers decreased over time (see Figure 8). The average length of a paper was 14.4, 10.1, and 8.4 pages in 1950, 1990, and 2014, respectively.

By analyzing citation patterns over time, we discovered that the percentage of papers with no citations after 5 years decreased (see Figure 9). Nevertheless, still about 72.1% of all papers published in 2009, and 25.6% of those with at least 5 references, were without any citations after 5 years (see Figure 9). Moreover, the total number of papers without any citations increased sharply (see Figure S11).

Additionally, by analyzing the citation distributions of papers published in different decades, we discovered citation distributions changed notably over time (see Figure 10).

Lastly, using the properties of over 3.29 million papers published between 1950 and 2009, we discovered positive correlations among the papers' citation numbers after 5 years and the following features: (a) title lengths (τ_S = 0.1); (b) author numbers (τ_S = 0.22); (c) abstract lengths (τ_S = 0.26); (d) keyword numbers (τ_S = 0.15); (e) reference numbers (τ_S = 0.48); (e) paper lengths (τ_S = 0.13); and (f) use of question or exclamation marks (τ_S = 0.022) (see Figure S13).[7]

Results of Author Trends

By analyzing the number of new authors each year, we discovered a sharp increase over time, with several million new authors publishing each year in recent years (see Figure S14).[8] Additionally, when analyzing the trends grouped by the authors' academic birth decades, we discovered a significant increase in the average number of published papers for the younger birth decades (see Figure 11). For example, researchers who started their careers in 1950 published on average 1.55 papers in a time period of 10 years, while researchers who started their careers in 2000 published on average 4.05 papers in the same time frame. Furthermore, we observed that authors who started their careers after 1990 tended to publish more in conferences in the first years of their career than their more senior peers who started their careers in the 1950s or 1970s (see Figure S15). For example, researchers who started their careers in the 1970s published on average about 2 conference papers and 1.65 journal papers after 10 years; researchers who started

7 Similar correlation values were obtained by calculating the correlations for papers published in a specific year.
8 It is possible that the same author has several MAG author IDs.

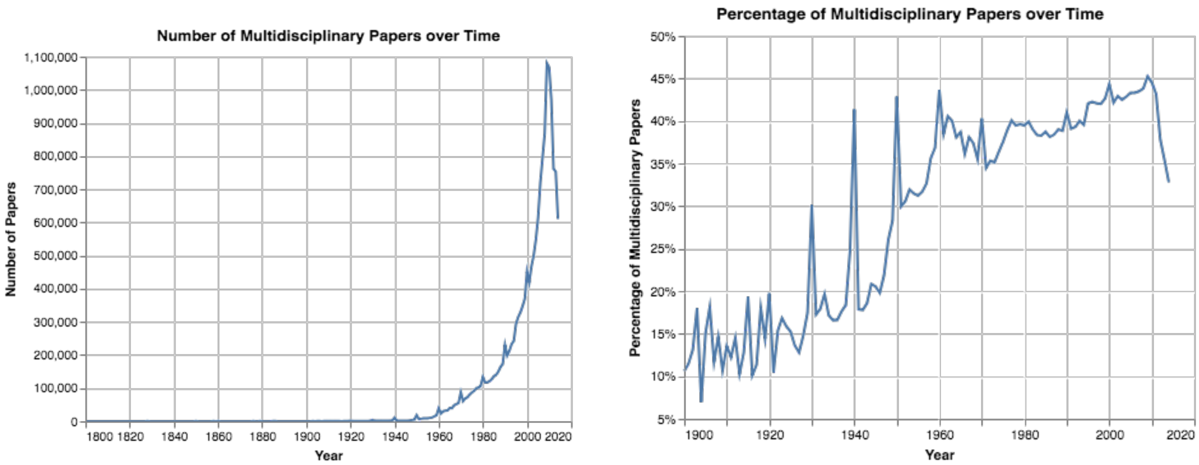

Figure 6. The Number and Percentage of Multidisciplinary Papers over Time. Between 1900 and 2010, both the number and percentage of multidisciplinary papers increased over time.

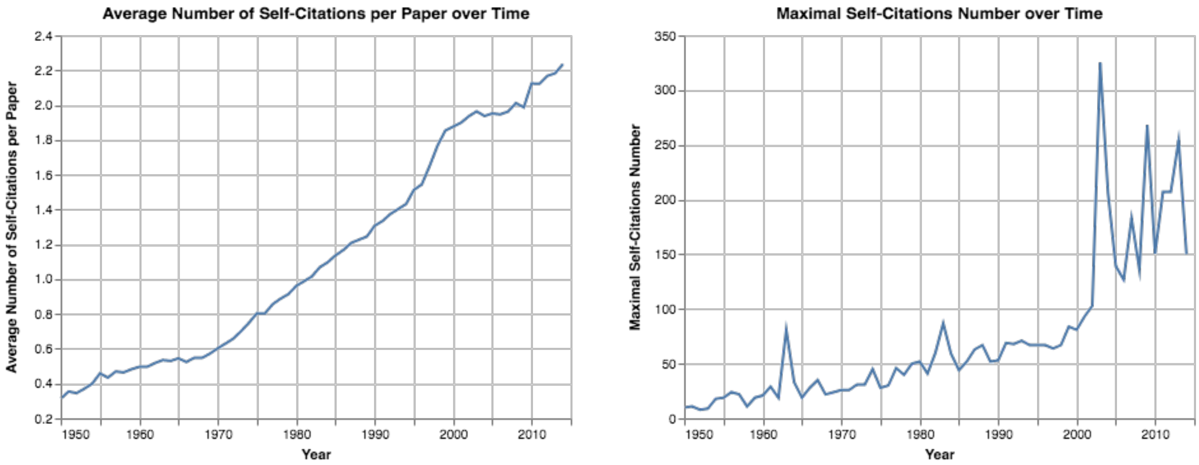

Figure 7. The Average and Maximal Number of Self-Citations. Both the average and maximal number of self-citations increased over time.

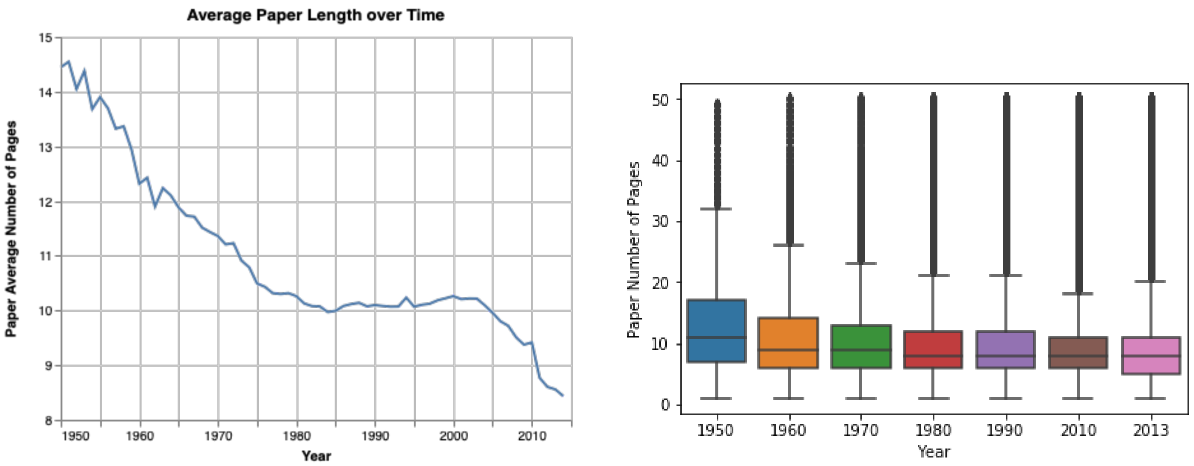

Figure 8. Papers' Lengths. Both the papers' average and median lengths decreased over time.

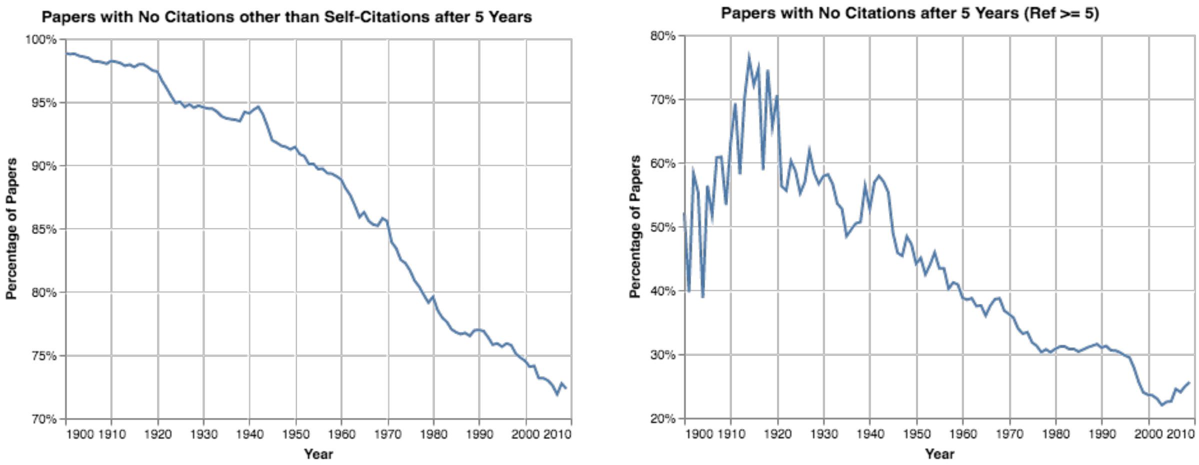

Figure 9. Papers with No Citations after 5 Years. Papers with no citations after 5 years decreased; nevertheless, in 2009 over 72.1% of all published papers had no citations after 5 years.

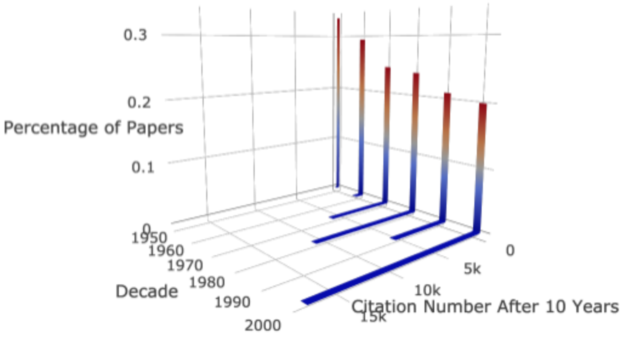

Figure 10. Citation Distributions over Time. The citation distributions of different decades show notable changes.

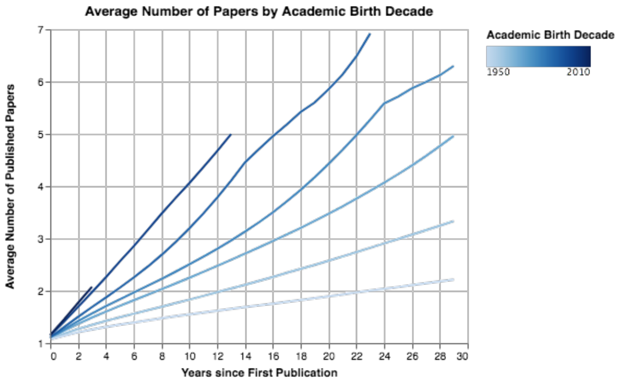

Figure 11. Average Number of Papers by Authors' Academic Birth Decades. With each decade, the rate of paper publication has increased.

their careers in the 2000s published about 4 conference papers and 2.59 journal papers in the same time frame.

We can also observe that the average number of coauthors has considerably increased over the decades (see Figure 12). Moreover, we can notice that researchers who started their careers in the 1950s and 1970s had on average only few coauthors over a period of 25 years, while researchers who started their careers in the 1990s had over 60 coauthors in the same career length of 25 years (see Figure 12).

Lastly, by exploring how author sequence numbers evolved, we discovered that with seniority, the researchers' median sequence number increased (see Figure S16). Additionally, with seniority, the percentage of published papers with the researcher listed as the first author decreased (see Figure 13). Moreover, by looking at the decade researchers started their careers, we can see a sharp decline in the percentages of first authors (see Figure 13). Overall, early career researchers are publishing more in their careers but appear as first authors much less than in previous generations.

Results of Journal Trends

By analyzing journal trends using the SJR and MAG datasets, we discovered that the number of journals increased significantly over the years, with 20,975 active ranked journals in 2016 (see Figure 14). Furthermore, we observed that hundreds of new ranked journals were published each year (see Figures S17 and S18). In addition, we discovered that the num-

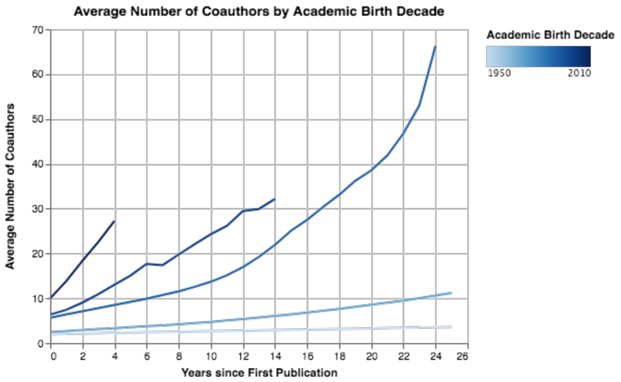

Figure 12. Average Number of Coauthors by Academic Birth Decade. The average number of coauthors has considerably increased over the decades.

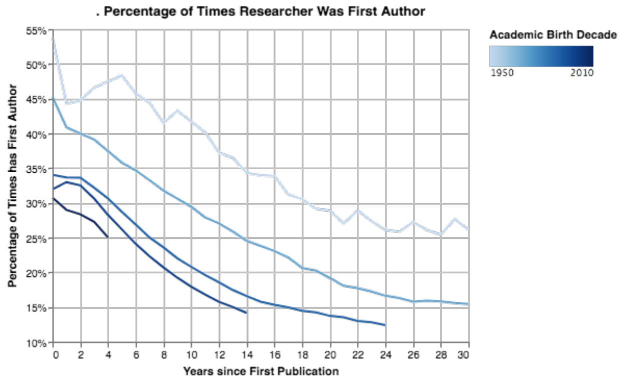

Figure 13. Percentage of Times Researcher was First Author. We can observe that over time on average the percentage of senior researchers as first authors declined. Moreover, in the same time intervals, the percentage of times recent generations of researchers were first authors declined compared to older generations.

ber of published papers per journal increased sharply, from an average of 74.2 papers in 1999 to an average of 99.6 papers in 2016 (see Figure 14). We also observed that in recent years, journals that publish thousands of papers have become more common. For example, in 2016, according to the SJR dataset, 197 journals published over 1000 papers each.

By exploring how various metrics have changed over time, we discovered the following: First, over the last 18 years, the number of papers published in Q1 and Q2 journals more than doubled, from 550,109 Q1 papers and 229,373 Q2 papers in 1999, to 1,187,514 Q1 papers and 554,782 Q2 papers in 2016 (see Figure 15). According to the SJR dataset, in 2016, 51.3% of journal papers were published in Q1 journals and only 8.66% were published in Q4 journals. Second, the average h-index decreased over recent years from an average value of 37.36 and median value of 23 in 1999 to an average value of 31.3 and median value of 16 in 2016 (see Figure S19). Third, we noted that the SJR and the average number of citations measures both increased considerably during the last 18 years (see Figures 16 and S20).

Besides the number of papers in top journals doubling between 2000 and 2014, the number of authors increased significantly (see Figure S21).[9] Additionally, by calculating the average academic career ages of first and last authors, we discovered that in recent years the average academic age has increased notably (see Figure 17). Moreover, when looking at first and last authors who previously published in one of the selected top-30 journals, we discovered that over time the percentage of returning authors increased substantially (see Figure 18). By 2014, 46.2% of all published papers in top-30 selected journals were published by last authors who had published at least one paper in a top-30 selected journal before (see Figure 18).

By calculating the number of papers, number of authors, authors' average age, and percentage of returning authors in each selected top-30 journal, we observed the following: (a) the number of published papers per year increased considerably in the vast majority of the journals (see Figure S22);[10] (b) the average career ages of last authors in the vast majority of the selected journals considerably increased (see Figure S23), like in Cell journal where the last authors' career ages increased from about 4.5 years in 1980 to about 20 years in 2014 (see Figure S23); and (c) the percentage of returning authors in the vast majority of the selected journals increased drastically, like in Nature Genetics where in 86.6% of 2014 papers, at least one of the authors had published in the journal before (see Figure 20).

Results of Fields-of-Research Trends

By matching each paper to its L0 field of study and analyzing each field's properties, we discovered substantial differences in these properties. Namely, we observed the following:

- A large variance in the number of published papers in each field. For example, 231,756 papers were published in the field of biology in 2010, but only 5,684 were published that year in the field of history (see Figures 19 and S24).
- A considerable variance in the average number of paper authors among the various research fields. For example, the number of authors in 2010 ranged from an average of 2.28 authors in the field of political science to an average of 5.39 authors in medicine (see Figure S25).
- A variance in the papers' average number of references in different fields. For example, in 2010, the average reference number in the fields of material science and engineering was less than 24, while in the fields of biology and history it was over 33 (see Figure S26).
- A big variance in each L0 field's average and median number of citations after 5 years. For example, for 2009 papers in the fields of computer science and political science, the median citation number after 5 years was 4 citations. In biology and environmental science, the median citation number after 5 years was 9 and 13 citations, respectively (see Figure 21).

By repeating the above analysis for the L1 subfields of biology and for the L2 subfields of genetics, we uncovered similar differences among fields of study. Namely, we observed the following for subfields in the same hierarchal level: (a) significant variance in the average number of papers (see Figures S27 and S28); (b) notable variance in the average number of authors (see Figures S29 and S30); (c) noteworthy variance in the average number of references (see Figures S31 and S32); and (d) vast variance in median citation numbers (see Figures S33 and S34).

Lastly, by analyzing various features of 2,673 L3 fields of study, we observed a huge variance in the different properties (see Table 1 and Figure S35). For example, several fields of study, such as gallium (chemistry), ontology (computer science), and presentation of a group (mathematics), had median citation numbers of 2, while other fields of study, such as microRNA and genetic recombination (biology), had median citation numbers of over 47 and 50.5, respectively (see Table 1 and Figure S35).

By analyzing the results presented in the Results section, the following can be noted: First, we can observe that the structure of academic papers has changed in distinct ways in recent decades. While the average overall length of papers has become shorter (see Figure 8), the title, abstract, and references have become longer (see the Results of Paper Trends section and Figures 3, 5, S3, S6, S9, and S10). Also, the number of papers that include keywords has increased considerably, as has the average number of keywords in each paper (see Figure S7). Furthermore, the average and median number of authors per paper has increased sharply (see Figures S3 and S4).

9 The total number of authors each year was determined by summing the number of authors in each published paper.

10 Due to missing references in the MAG dataset, there are decline in the number of papers in Nature (1990s), and in Science (before 2008).

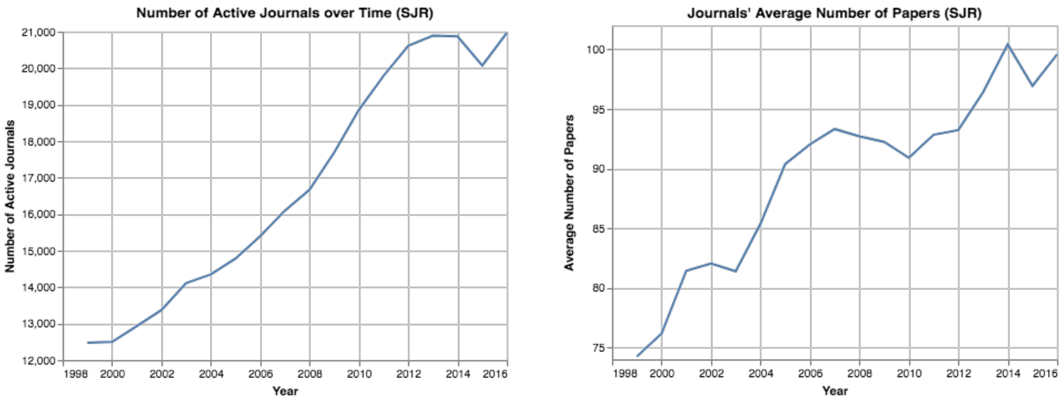

Figure 14. Number of Active Journals over Time. Over a period of 18 years, from 1999 to 2016, both the number of active journals and the papers per journal increased greatly.

]

Table 1. L3 Fields-of-Study Features in 2009

Parent Field of Study	**Field of Study Name**	**Median Citations After 5 Years**	**MAX Citations After 5 Years**	**Number of Papers**	**Average Author Number**
Engineering	**Structural material**	61.0	1250	174	6.14
Biology	**Genetic recombination**	50.5	451	196	6.07
Biology	**Nature**	48.0	5660	4162	6.28
Biology	**microRNA**	47.0	3076	1691	6.24
Biology	**Induced pluripotent stem cell ...**	39.0	987	213	6.53
Economics	**Signalling**	39.0	695	1030	5.87
Biology	**Genome evolution**	35.5	392	140	5.04
Biology	**Non-coding RNA**	35.0	1414	375	5.39
Biology	**Post-transcriptional modification ...**	34.0	1414	315	5.49
Biology	**Autophagy**	34.0	789	381	5.71
•	•	•	•	•	•
•	•	•	•	•	•
•	•	•	•	•	•
Mathematics	**Finite impulse response**	2.0	167	337	3.0
Computer Science	**Pixel**	2.0	380	2484	3.27
Computer Science	**Ontology**	2.0	616	733	3.35
Computer Science	**Mesh networking**	2.0	62	274	3.43
Computer Science	**Camera resectioning**	2.0	43	114	3.13
Computer Science	**Session Initiation Protocol ...**	2.0	116	100	3.6
Chemistry	**Gallium**	2.0	73	484	3.43
Mathematics	**Presentation of a group**	2.0	91	706	3.22
Mathematics	**Spiral**	2.0	80	122	3.65
Mathematics	**Block code**	2.0	54	281	2.83

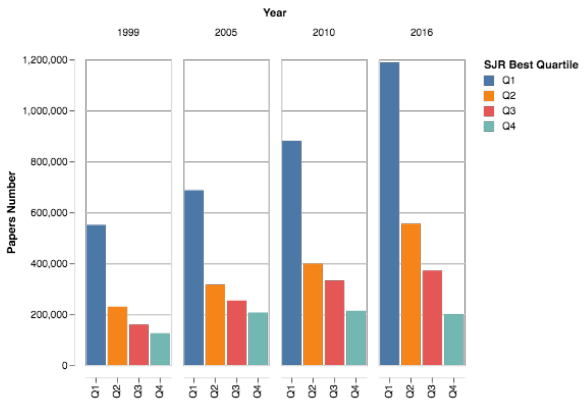

Figure 15. Journals' Quartile Number of Papers over Time. The number of papers published in Q1 journals has vastly increased.

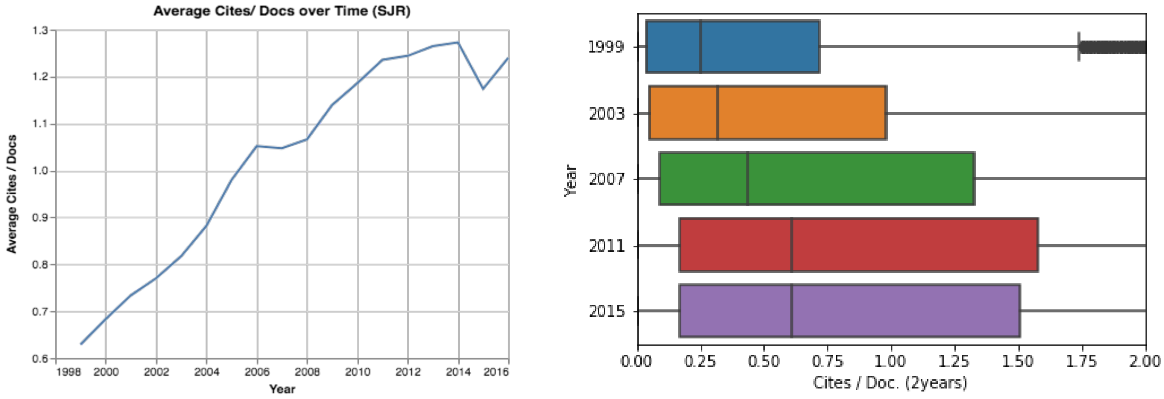

Figure 16. The Average Number of Citations ($\frac{Cites}{Docs}$ (2 years)) over Time. The average number of citations values have almost doubled in the last 18 years; additionally, their distributions have changed considerably.

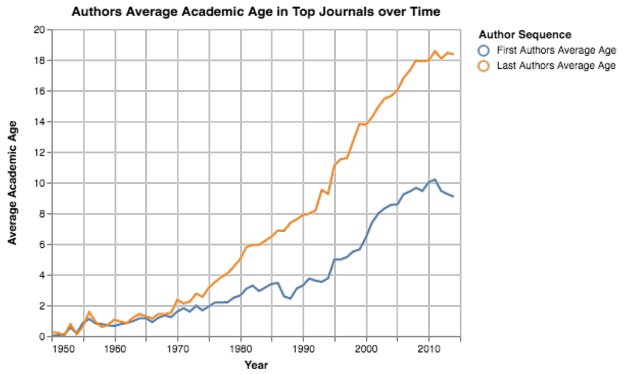

Figure 17. Top-Selected Journals' Average First and Last Authors Ages. Both the first and last authors' average ages have increased sharply.

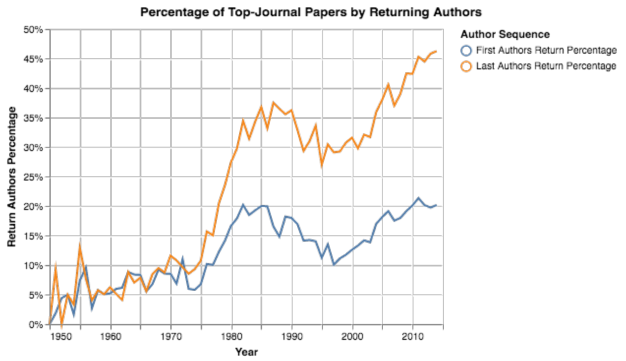

Figure 18. Percentage of Papers with Returning First or Last Authors. The percentage of returning first or last top-journal authors increased considerably.

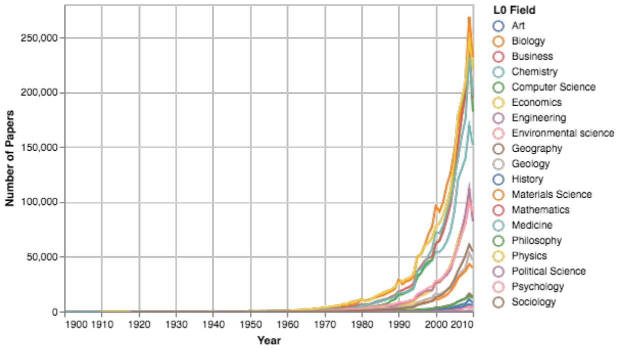

Figure 19. L0 Fields-of-Study Number of Papers over Time. The numbers of papers in each field of study have increased drastically.

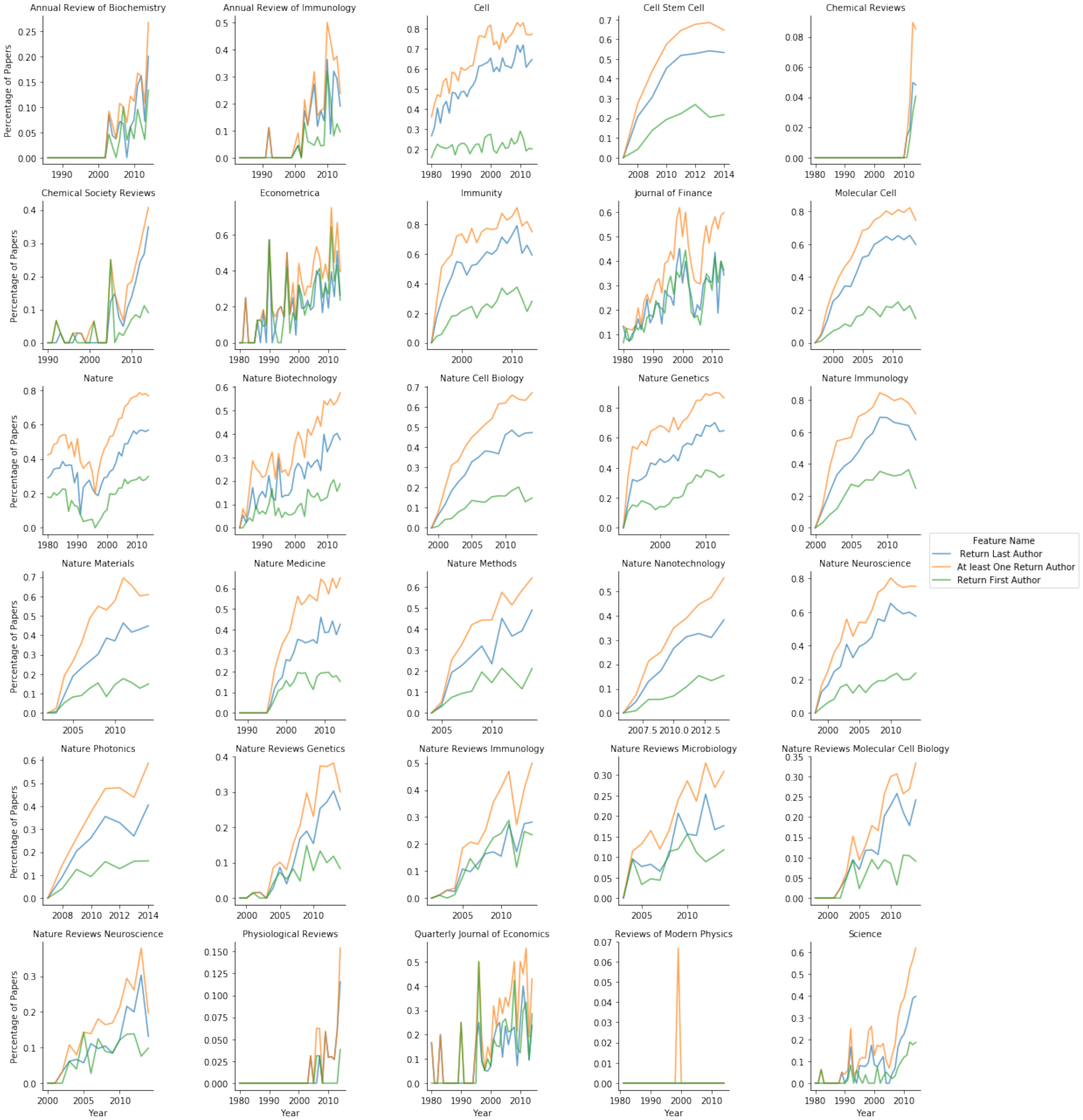

Figure 20. Average Percentage of Return Authors in Top-Selected Journals over Time. In most journals the number of papers with at least one author who previously published in the journal increased sharply. In many of the selected journals the percentage of papers with returning authors was above 60%, and in some cases above 80%.

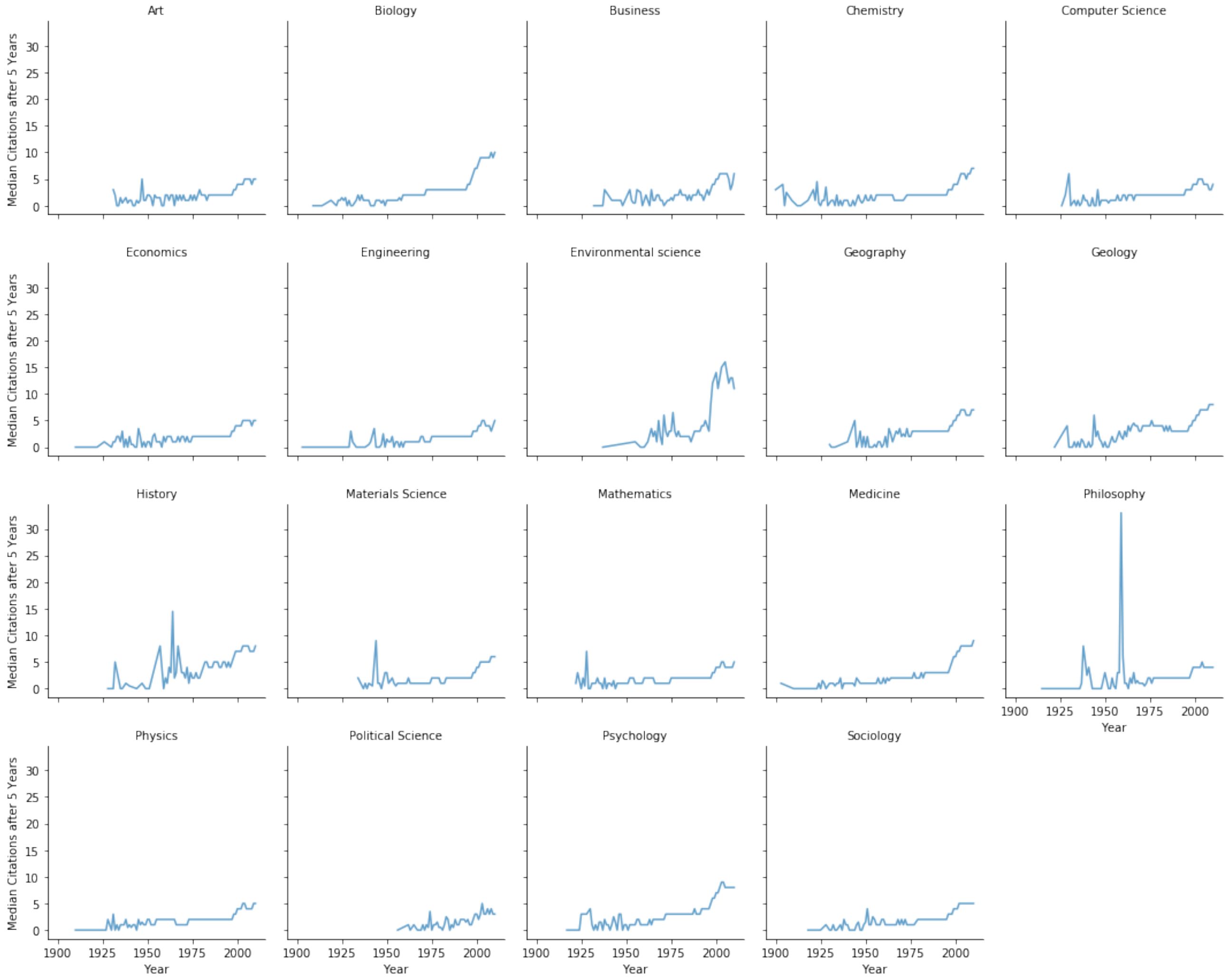

Figure 21. L0 Field-of-Study Median Citation Number after 5 Years. There is notable variance among the L0 fields-of-study median citation numbers.

Figure 22: Measuring success in academic publishing

A comprehensive analysis of more than 120 million academic papers by 35 million authors published since the early 19th century reveals that the world of academic publishing is changing significantly across 19 major research domains and 2,600 research fields. Here we examine trends from 1990 to 2010, with a closer look at increasingly specialized fields within biology and genetics.

The number of papers has surged in recent decades, particularly in non-English languages. Titles have increased in length, as has the use of emphatic punctuation such as question marks and exclamation points.

The number of authors has risen, as has collaboration: Researchers who started their careers in the 1950s on average had a few coauthors over 25 years, but those who started in the 1990s average more than 60 over a similar period.

The number of ranked journals has exploded, surpassing 21,000 active journals in 2016 and adding several hundred new ones each year..

The number of references is growing across disciplines, even as papers' lengths have decreased.

Self-citations have increased along with citations in general, but papers' influences are not long lasting: 72% of papers did not have a single citation 5 years after publication.

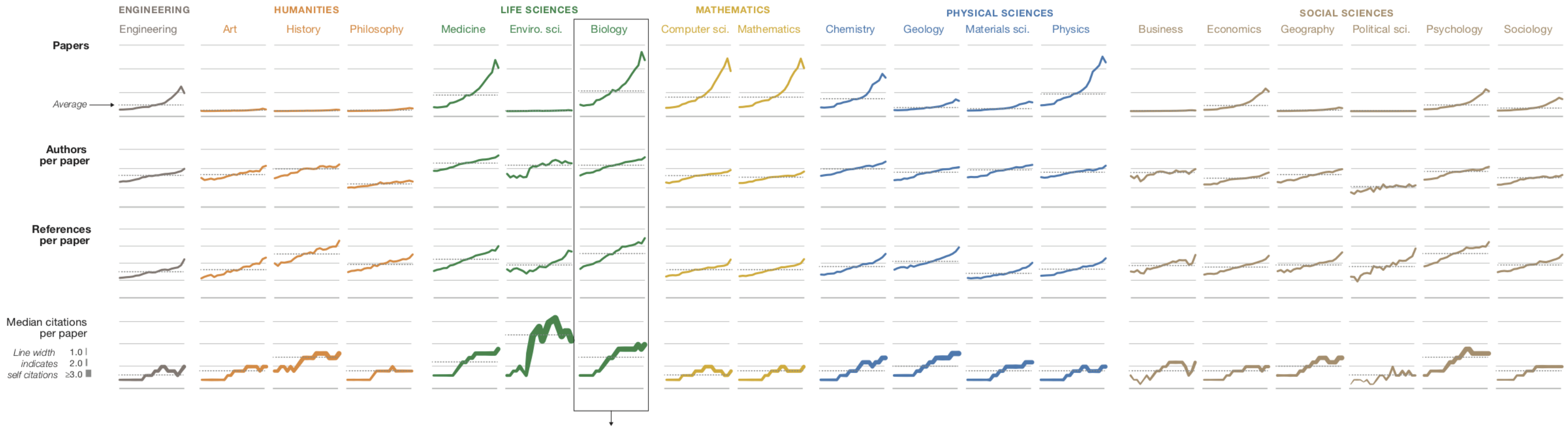

Trends within biology and genetics

The number of biology-related papers published per year has surged, mirroring trends in medicine, physics, computer science and mathematics.

But this rate of growth is not consistent among disciplines within biology, where research in genetics is growing quickly but cell biology continues at a relatively steady state.

Within genetics disciplines, epigenetics publishing is on the rise.

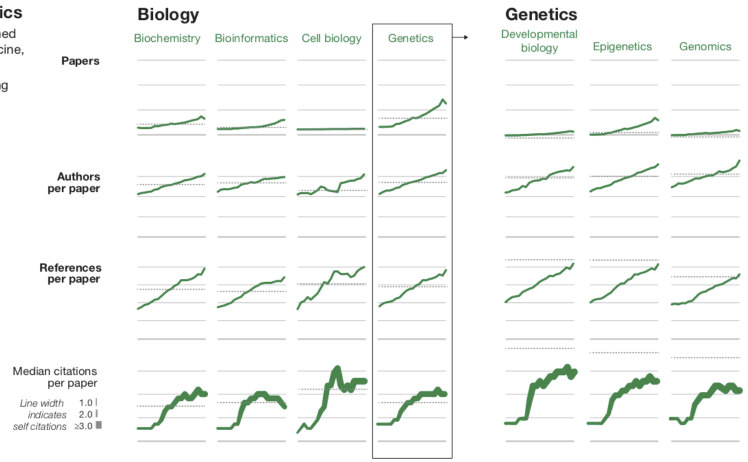

CHARTS: SEAN McNAUGHTON

Discussion

Below we discuss nine aspects of our study that provide insights into current academic publishing trends, and we explore the potential impact of our results.

First, these results support Goodhart's Law as it relates to academic publishing: the measures (e.g., number of papers, number of citations, h-index, and impact factor) have become targets, and now they are no longer good measures. By making papers shorter and collaborating with more authors, researchers are able to produce more papers in the same amount of time. Moreover, we can observe that the majority of changes in papers' properties are correlated with papers that receive higher numbers of citations (see Figure S13). Authors can use longer titles and abstracts, or use question or exclamation marks in titles, to make their papers more appealing. Thus more readers are attracted to the paper, and ideally they will cite it, i.e., academic clickbait [45]. These results support our hypothesis that the citation number has become a target. Consequently, the properties of academic papers have evolved in order to win–to score a bullseye on the academic target.

It is worth noting that while the study's results provide evidence that many citation-based measures have become targets, there also may be other factors that influence academic publication trends. For example, the academic hypercompetitive environment itself may prompt an increase in productivity [81], hence increasing the number of papers. However, this claim contradicts Fanelli and Larivière findings that researchers' individual productivity did not increase in the last century [52]. Nevertheless, it is important to keep in mind that there may be other underlying factors that contributed to the observed results.

Second, we observed that over time fewer papers list authors alphabetically, especially papers with a relatively high number of authors (see Results of Paper Trends section and Figures 4 and S5). These results may indicate the increased importance of an author's sequence number in a paper, which may reflect the author's contribution to the study. This result is another signal of the rising importance of measures that rate an individual's research contribution.

Third, from matching papers to their L0 fields of study, we observed that the number of multidisciplinary papers has increased sharply over time (see Figure 6). It is important to keep in mind that these results were obtained by matching keywords to their corresponding fields of study. Therefore, these results have several limitations: First, not all papers contain keywords. Second, the dataset may not extract keywords from papers in the correct manner. For example, we found some papers contained keywords in their online version but not in their offline version (see Results of Paper Trends section). It is also possible that in some fields it is less common to use keywords. Therefore, the papers' keywords may be missing in the datasets, and the presented results may be an underestimate of the actual number of multidisciplinary studies. Nevertheless, we observed a strong trend in increasing numbers of multidisciplinary papers.

Fourth, from seeing sharp increases in both the maximal and average number of self-citations (see Results of Paper Trends section and Figures 7, 9, 10, and S12), it is clear that citation numbers have become a target for some researchers who cite their own papers dozens, or even hundreds, of times. Furthermore, we can observe a general increasing trend for researchers to cite their previous work in their new studies. Moreover, from analyzing the percentage of papers without citations after 5 years, we can observe that a huge quantity of papers – over 72% of all papers and 25% of all papers with at least 5 references – have no citations at all (see Figure 9). Obviously, many resources are spent on papers with limited impact. The lack of citations may indicate that researchers are publishing more papers of poorer quality to boost their total number of papers. Additionally, by exploring papers' citation distributions (see Figure 10), we can observe that different decades have very different citation distributions. This result indicates that comparing citation records of researchers who published papers in different time periods can be challenging.

Fifth, by exploring trends in authors (see Results of Author Trends section and Figures 11, 12, 13, S14, S15, and S16), we observed an exponential growth in the number of new researchers who publish papers. We also observed that young career researchers tend to publish considerably more than researchers in previous generations, using the same time frames for comparison (see Figure 11). Moreover, young career researchers tend to publish their work much more in conferences in the beginning of their careers than older researchers did in previous decades (see Figure S15). We also observed that young career researchers tend to collaborate considerably more in the beginning of their careers than those who are older (see Figure 12). Furthermore, we see that the average percentage of researchers as first authors early in their career is considerably less than those in previous generations (see Figure 13). In addition, authors' median sequence numbers typically increase over time, and the rate is typically faster for young career researchers (see Figure S16). These results emphasize the changes in academia in recent years. In a culture of "publish or perish," researchers publish more by increasing collaboration (and being added to more author lists) and by publishing more conference papers than in the past. However, as can be observed by the overall decline of researchers as first authors, young career researchers may be publishing more in their careers but contributing less to each paper. The numbers can be misleading: a researcher who has 5 "first author" claims but has published 20 papers may be less of a true contributor than one with 4 "first author" claims and 10 published papers.

Sixth, by analyzing journal trends (see Results of Journal Trends section), we see a rapid increase in the number of ranked active journals in recent years (see Figure 14). Moreover, on average, journals publish more papers than in the past, and dozens of journals publish over 1,000 papers each year (see Figure 14 and S17). With the increase in the number of active journals, we observed rapid changes in impact measures: (a) the number of papers published in the first and second quartiles (Q1 and Q2) has increased sharply, and today the vast majority of papers are published in these quartiles (see Figure 15); (b) the journals' average and median h-index has decreased sharply (see Figure S18); and (c) both the SJR and the average number of citations has increased considerably (see Figures 16 and S20). With these significant changes, it is clear that some measures, such as the use of quartiles and the h-index, are rapidly losing meaning and value. Moreover, with the abundance of journals, researchers can "shop around" for a high impact journal and submit a rejected paper from one Q1 journal to another Q1 journal, time after time, and then start the review process again. These repeated reviews for the same paper wastes time, and in the long run the burden of reviewing papers several times may affect the quality of the reviews.

There are compelling reasons to change the current system. We need to think about making all reviews open and online. We should consider the function of published journals; for that matter, is it even necessary to have journals in a world with over 20,000 journals that publish hundreds or even thousands of papers each year? We need to seriously evaluate the measures we use to judge research work. If all these measures have been devalued to being merely targets, they are no longer effective measures. Instead, they should be adapted to meet our current needs and priorities. Moreover, today there are alternative measures to evaluate researchers' contributions and

journals' impacts (see Background section). It would be beneficial to the academic community to promote the use of these measures, while concurrently raising awareness of the many limitations of the traditional measures that are still commonly used.

Seventh, by focusing on trends in selected top journals, we can observe that these journals have changed considerably in recent years (see Figures 17, 18, 20, S21, and S22). The number of papers in the selected journals has increased sharply, along with the career age of the authors and the percentage of returning authors. The number of submissions to top journals, like Nature, have increased greatly in recent years [82]; however, many of these journals mainly publish papers in which at least one of the authors has previously published in the journal (see Figure 18 and 20). We believe that this situation is also a result of Goodhart's Law. The target is the impact factor, and so researchers are vigorously seeking journals with high impact factors. Therefore, the yearly volume of papers sent to these top journals has considerably increased, and overwhelmed by the volume of submissions, editors at these journals may choose safety over risk and select papers written by only well-known, experienced researchers.

Eighth, by analyzing how features evolve in the various L0 fields of study using the MAG dataset, we can observe that different fields have completely different sets of features (see Figures 19, 21, 19, S25, S26, and Table 1). While some fields have hundreds of thousands of papers published yearly, others have only thousands published yearly (see Figures 19 and S22). Moreover, similar large differences are reflected in other examined fields' features, such as the average number of references and the average and median citation numbers (see Figures 21 and S35).

Lastly, by examining over 2600 research fields of various scales (see Table 1 and Figure S35), we observed vast diversity in the properties of papers in different domains – some research domains grew phenomenally while others did not. Even research domains in the same subfields presented a wide range of properties, including papers' number of references and median number of citations per research field (see Table 1 and Figures S31, S32, S33, and S34). These results indicate that using measures such as citation number, h-index, and impact factor are useless when comparing researchers in different fields, and even for comparing researchers in the same subfield, such as genetics. These results emphasize that using citation-based measures for comparing various academic entities is like comparing apples to oranges, and is to "discriminate between scientists." [55]. Moreover, using these measures as gauges to compare academic entities can drastically affect the allocation of resources and consequently damage research. For example, to improve their world ranking, universities might choose to invest in faculty for computer science and biology, rather than faculty for less-cited research fields, such as economics and psychology. Moreover, even within a department, the selection of new faculty members can be biased due to using targeted measures, such as citation number and impact factor. A biology department might hire genetic researchers in the field of epigenetics, instead of researchers in the field of medical genetics, due to the higher average number of citations in the epigenetics field. Over time, this can unfairly favor high-citation research fields at the expense of other equally significant fields.

Conclusions

In this study, we performed a large-scale analysis of academic publishing trends, utilizing data on over 120 million papers and over 20,000 journals. By analyzing this huge dataset, we can observe that over the last century, especially the last few decades, published research has changed considerably, including the numbers of papers, authors, and journals; the lengths of papers; and the average number of references in specific fields of study (see Figure 22).

While the research environment has changed, many of the measures to determine the impact of papers, authors, and journals have not changed. Even with the development of some new and better measures, the academic publishing world too often defaults to the traditional measures based on citations, such as impact factor and citation number, that were used 60 years ago, in a time before preprint repositories and mega-journals existed and before academia became such a hyper-competitive environment. Most important, however, is that these measures have degenerated into becoming purely targets. Goodhart's Law is clearly being illustrated: when a citation-based measure becomes the target, the measure itself ceases to be meaningful, useful, or accurate.

Our study's extensive analysis of academic publications reveals why using citation-based metrics as measures of impact are wrong from the core: First, not all citations are equal; there is a big difference between a study that cites a paper that greatly influenced it and a study that cites multiple papers with only minor connections. Many of the impact measures widely used today do not take into consideration distinctions among the various types of citations. Second, it is not logical to measure a paper's impact based on the citation numbers of other papers that are published in the same journal. In the academic world, there are over 20,000 journals that publish hundreds or even thousands of papers each year, with papers written by hundreds or even thousands of authors. It is even less logical to measure a researcher's impact based on a paper coauthored with many other researchers according to the journal in which it is published. Third, as we demonstrated in the Results of Fields-of-Research Trends section, it is wrong to compare studies from different fields, and even to compare papers and researchers within the same parent field of study, due to the many differences in the median and average number of citations in each field (see Table 1).

As we have revealed in this study, to measure impact with citation-based measures—that have now become targets—clearly has many undesirable effects. The number of papers with limited impact has increased sharply (see Figure S11), papers may contain hundreds of self-citations (see Figure 7), and some top journals have become "old boys' clubs" that mainly publish papers from the same researchers (see Figures 17 and 18). Moreover, using citation-based measures to compare researchers in different fields may have the dangerous effect of allocating more resources to high-citation domains, shortchanging other domains that are equally important.

We believe the solution to the above issues is to utilize data-science tools and release new and open datasets in order to promote using existing unbiased measures or to develop new measures that will more accurately determine a paper's impact in a specific research field. Moreover, it is vital to raise awareness of the shortcomings of commonly used measures, such as the number of citations, h-index, and impact factor. Certain metrics have been proposed, but the key is to wisely and carefully evaluate new measures to ensure that they will not follow Goodhart's Law and end up merely as targets. Researchers do valuable work. Communicating the work to others is vital, and correctly assessing the impact of that work is essential.

Methods

To analyze the above MAG and AMiner large-scale datasets, we developed an open source framework written in Python, which provided an easy way to query the datasets. The framework

utilizes TuriCreate's SFrame dataframe objects [83] to perform big-data analysis on tens of millions of records to calculate how various properties have changed over time. For example, we used SFrame objects to analyze how the average number of authors and title lengths evolved. However, while SFrame is exceptionally useful for calculating various statistics using all-papers features, it is less convenient and less computationally cost effective for performing more complicated queries, such as calculating the average age of the last authors in a certain journal in a specific year.

To perform more complex calculations, we loaded the datasets into the MongoDB database [84]. Next, we developed a code framework that easily let us obtain information on papers, authors, paper collections, venues, and research fields. The framework supports calculating complex features of the above object in a straightforward manner. For example, with only a few and relative simple lines of Python code, we were able to calculate the average number of coauthors per author in a specific year for authors who started their career in a specific decade. An overview of our code framework is presented in Figure S1.

To make our framework accessible to other researchers and to make this study completely reproducible, we have written Jupyter Notebook tutorials which demonstrate how the SFrame and MongoDB collections were constructed from the MAG, AMiner, and SJR datasets (see Availability of Source Code and Requirements section and RRID SCR_016958).

Availability of Supporting Data

An interactive web interface to explore the study's data is available at the project's website. The web interface provides researchers the ability to interactively explore and better understand how various journals' properties have changed over time (see Figure S36 and RRID: SCR_016958). Additionally, the website contains the *Fields-of-Research Features* data.

Availability of Source Code and Requirements

One of the main goals of this study was to create an open source framework, which provided an easy way to query the datasets. Our code framework, including tutorials, is available at the project's website.

Availability of source code and requirements:

- Project name: Science Dynamics
- Project home page: sciencedynamics.cs.washington.edu
- Operating system(s): Platform independent
- Programming language: Python
- Other requirements: Python 2.7, MongoDB, TuriCreate Python Package
- License: MIT License
- RRID: SCR_016958

Declarations

Competing interests

The authors declare no competing financial interests.

Author Contributions

Both MF and CG conceived the concept of this study and developed the methodology. MF developed the study's code and visualization, and performed the data computational analysis. CG supervised the research.

Funding

This research was supported by the Washington Research Foundation Fund for Innovation in Data-Intensive Discovery, the Moore/Sloan Data Science Environments Project at the University of Washington, the AWS Cloud Credits for Research, and the Microsoft Azure Research Award.

Acknowledgements

First and foremost, we would like to thank the AMiner, Microsoft Academic Graph, and SJR teams for making their datasets available online. Additionally, we thank the AWS Cloud Credits for Research. We also thank the Washington Research Foundation Fund for Innovation in Data-Intensive Discovery, the Moore/Sloan Data Science Environments Project at the University of Washington, and Microsoft Azure Research Award for supporting this study. Furthermore, we wish to thank the reviewers and the editors for their insightful comments which we feel have substantially improved our paper. In addition, we wish to thank to Lior Rokach and Yuval Shahar for their helpful suggestions. Datasets, software implementations, code tutorials, and an interactive web interface for investigating the studied networks are available at this link.

We also wish to especially thank Carol Teegarden for editing and proofreading this article to completion, and to Sean McNaughton for designing and illustrating the article's infographic.

References

1. Ware M, Mabe M. The STM report: An overview of scientific and scholarly journal publishing 2015;.
2. Herrmannova D, Knoth P. An analysis of the microsoft academic graph. D-Lib Magazine 2016;22(9/10).
3. Björk BC. Have the "mega-journals" reached the limits to growth? PeerJ 2015;3:e981.
4. Kelly S, The continuing evolution of publishing in the biological sciences. The Company of Biologists Ltd; 2018.
5. Roemer RC, Borchardt R. From bibliometrics to altmetrics: A changing scholarly landscape. College & Research Libraries News 2012;73(10):596–600.
6. Hirsch JE. An index to quantify an individual's scientific research output. Proceedings of the National academy of Sciences 2005;102(46):16569–16572.
7. Garfield E. The agony and the ecstasy—the history and meaning of the journal impact factor 2005;.
8. Wilsdon J. The metric tide: Independent review of the role of metrics in research assessment and management. Sage; 2016.
9. Edwards MA, Roy S. Academic research in the 21st century: Maintaining scientific integrity in a climate of perverse incentives and hypercompetition. Environmental Engineering Science 2017;34(1):51–61.
10. Biagioli M. Watch out for cheats in citation game. Nature News 2016;535(7611):201.
11. Campbell DT. Assessing the impact of planned social change. Evaluation and program planning 1979;2(1):67–90.
12. Newton AC. Implications of Goodhart's Law for monitoring global biodiversity loss. Conservation Letters 2011;4(4):264–268.
13. Mizen P. Central Banking, monetary theory and practice:

essays in honour of Charles Goodhart, vol. 1. Edward Elgar Publishing; 2003.
14. Chrystal KA, Mizen PD, Mizen P. Goodhart's Law: its origins, meaning and implications for monetary policy. Central banking, monetary theory and practice: Essays in honour of Charles Goodhart 2003;1:221–243.
15. Francescani C, NYPD Report Confirms Manipulation of Crime Stats. Reuters; 2012.
16. Kliger AS. Quality measures for dialysis: Time for a balanced scorecard. Clinical Journal of the American Society of Nephrology 2016;11(2):363–368.
17. Šupak Smolčić V. Salami publication: Definitions and examples. Biochemia medica: Biochemia medica 2013;23(3):237–241.
18. Schofferman J, Wetzel FT, Bono C. Ghost and guest authors: You can't always trust who you read. Pain Medicine 2015;16(3):416–420.
19. Head ML, Holman L, Lanfear R, Kahn AT, Jennions MD. The extent and consequences of p-hacking in science. PLoS biology 2015;13(3):e1002106.
20. Bartneck C, Kokkelmans S. Detecting h-index manipulation through self-citation analysis. Scientometrics 2010;87(1):85–98.
21. Kupferschmidt K, Tide of lies. American Association for the Advancement of Science; 2018.
22. Haug CJ. Peer-review fraud—hacking the scientific publication process. New England Journal of Medicine 2015;373(25):2393–2395.
23. Dansinger M. Dear plagiarist: a letter to a peer reviewer who stole and published our manuscript as his own. Annals of internal medicine 2017;166(2):143–143.
24. Post A, Li AY, Dai JB, Maniya AY, Haider S, Sobotka S, et al. c-index and Subindices of the h-index: New Variants of the h-index to Account for Variations in Author Contribution. Cureus 2018;10(5).
25. Romanovsky AA, Revised h index for biomedical research. Taylor & Francis; 2012.
26. Wu Q. The w-index: A measure to assess scientific impact by focusing on widely cited papers. Journal of the American Society for Information Science and Technology 2010;61(3):609–614.
27. Waltman L, van Eck NJ, van Leeuwen TN, Visser MS, van Raan AF. Towards a new crown indicator: An empirical analysis. Scientometrics 2011;87(3):467–481.
28. Google Scholar - Top publications; (Accessed: 13-February-2019). /https://scholar.google.com/citations?view_op=top_venues.
29. Journal Citation Reports (JCR); (Accessed: 16-February-2019). /https://jcr.incites.thomsonreuters.com/.
30. Fortunato S, Bergstrom CT, Börner K, Evans JA, Helbing D, Milojević S, et al. Science of science. Science 2018;359(6379):eaao0185.
31. arXiv, arXiv Monthly Submission Rates; (Accessed: 20-January-2019). /https://arxiv.org/stats/monthly_submissions.
32. Learn JR. What bioRxiv's first 30,000 preprints reveal about biologists. Nature News 2019;.
33. Davis P, Scientific Reports Overtakes PLOS ONE As Largest Megajournal - The Scholarly Kitchen; (Accessed: 9-July-2018). /https://scholarlykitchen.sspnet.org/2017/04/06/scientific-reports-overtakes-plos-one-as-largest-megajournal/.
34. Cronin B. Hyperauthorship: A postmodern perversion or evidence of a structural shift in scholarly communication practices? Journal of the American Society for Information Science and Technology 2001;52(7):558–569.
35. Von Bergen C, Bressler MS. Academe's Unspoken Ethical Dilemma: Author Inflation in Higher Education. Research in Higher Education Journal 2017;32.
36. Mallapaty S. Paper authorship goes hyper Author. Nature Index News 2018;.
37. Abbott BP, Abbott R, Abbott T, Abernathy M, Acernese F, Ackley K, et al. Observation of gravitational waves from a binary black hole merger. Physical review letters 2016;116(6):061102.
38. Castelvecchi D. LIGO's unsung heroes. Nature News 2017;.
39. Aboukhalil R. The rising trend in authorship. The winnower 2014;2:e141832.
40. Wislar JS, Flanagin A, Fontanarosa PB, DeAngelis CD. Honorary and ghost authorship in high impact biomedical journals: a cross sectional survey. Bmj 2011;343:d6128.
41. Kennedy MS, Barnsteiner J, Daly J. Honorary and ghost authorship in nursing publications. Journal of Nursing Scholarship 2014;46(6):416–422.
42. Vera-Badillo FE, Napoleone M, Krzyzanowska MK, Alibhai SM, Chan AW, Ocana A, et al. Honorary and ghost authorship in reports of randomised clinical trials in oncology. European Journal of Cancer 2016;66:1–8.
43. Economist T. Why research papers have so many authors. The Economist 2016;/http://www.economist.com/news/science-and-technology/21710792-scientific-publications-are-getting-more-and-more-names-attached-them-why.
44. Lewison G, Hartley J. What's in a title? Numbers of words and the presence of colons. Scientometrics 2005;63(2):341–356.
45. Lockwood G. Academic clickbait: articles with positively-framed titles, interesting phrasing, and no wordplay get more attention online. The Winnower 2016;3.
46. Ucar I, López-Fernandino F, Rodriguez-Ulibarri P, Sesma-Sanchez L, Urrea-Micó V, Sevilla J. Growth in the number of references in engineering journal papers during the 1972–2013 period. Scientometrics 2014;98(3):1855–1864.
47. Gálvez A, Maqueda M, Martinez-Bueno M, Valdivia E. Scientific Publication Trends and the Developing World: what can the volume and authorship of scientific articles tell us about scientific progress in various regions? American scientist 2000;88(6):526–533.
48. Jagsi R, Guancial EA, Worobey CC, Henault LE, Chang Y, Starr R, et al. The "gender gap" in authorship of academic medical literature—a 35-year perspective. New England Journal of Medicine 2006;355(3):281–287.
49. Johnson MR, Wagner NJ, Reusch J. Publication trends in top-tier journals in higher education. Journal of Applied Research in Higher Education 2016;8(4):439–454.
50. Aldhous P. Scientific publishing: The inside track. Nature 2014;.
51. Porter A, Rafols I. Is science becoming more interdisciplinary? Measuring and mapping six research fields over time. Scientometrics 2009;81(3):719–745.
52. Fanelli D, Larivière V. Researchers' individual publication rate has not increased in a century. PLoS One 2016;11(3):e0149504.
53. Dong Y, Ma H, Shen Z, Wang K. A century of science: Globalization of scientific collaborations, citations, and innovations. In: Proceedings of the 23rd ACM SIGKDD International Conference on Knowledge Discovery and Data Mining ACM; 2017. p. 1437–1446.
54. Yan E, Ding Y. Weighted citation: An indicator of an article's prestige. Journal of the American Society for Information Science and Technology 2010;61(8):1635–1643.
55. Lehmann S, Jackson AD, Lautrup BE. Measures for measures. Nature 2006;444(7122):1003.
56. Garfield E. The meaning of the impact factor. International Journal of Clinical and Health Psychology 2003;3(2).
57. Hirsch JE. Does the h index have predictive power?

Proceedings of the National Academy of Sciences 2007;104(49):19193–19198.
58. Fong EA, Wilhite AW. Authorship and citation manipulation in academic research. PloS one 2017;12(12):e0187394.
59. Editorial N. The cost of salami slicing. Nature Materials 2005;4:1.
60. Delgado López-Cózar E, Robinson-García N, Torres-Salinas D. The Google scholar experiment: How to index false papers and manipulate bibliometric indicators. Journal of the Association for Information Science and Technology 2014;65(3):446–454.
61. Van Bevern R, Komusiewicz C, Niedermeier R, Sorge M, Walsh T. H-index manipulation by merging articles: Models, theory, and experiments. Artificial Intelligence 2016;240:19–35.
62. Falagas ME, Kouranos VD, Arencibia-Jorge R, Karageorgopoulos DE. Comparison of SCImago journal rank indicator with journal impact factor. The FASEB journal 2008;22(8):2623–2628.
63. Lariviere V, Kiermer V, MacCallum CJ, McNutt M, Patterson M, Pulverer B, et al. A simple proposal for the publication of journal citation distributions. BioRxiv 2016;p. 062109.
64. Callaway E. Beat it, impact factor! Publishing elite turns against controversial metric. Nature 2016;.
65. Altmetric; (Accessed: 14-February-2019). /https://www.altmetric.com/.
66. Griffin SA, Oliver CW, Murray A, 'Altmetrics'! Can you afford to ignore it? BMJ Publishing Group Ltd and British Association of Sport and Exercise Medicine; 2017.
67. AI2, Semantic Scholar; (Accessed: 14-February-2019). /https://www.semanticscholar.org.
68. Seglen PO. Why the impact factor of journals should not be used for evaluating research. BMJ: British Medical Journal 1997;314(7079):498.
69. Byrne A. Comment: Measure for measure. Nature 2017;.
70. Hecht F, Hecht BK, Sandberg AA. The journal "impact factor": a misnamed, misleading, misused measure. Cancer genetics and cytogenetics 1998;104 2:77–81.
71. Google Scholar; (Accessed: 13-February-2019). /https://scholar.google.com.
72. Sinha A, Shen Z, Song Y, Ma H, Eide D, Hsu BJP, et al. An Overview of Microsoft Academic Service (MAS) and Applications. In: WWW; 2015. .
73. 2016 KC, SIGKDD: KDD Cup 2016: Whose papers are accepted the most: towards measuring the impact of research institutions; 2016. /http://www.kdd.org/kdd-cup/view/kdd-cup-2016/Data.
74. Semantic Scholar - An Overview of Microsoft Academic Service (MAS) and Applications; (Accessed: 14-February-2019). /https://www.semanticscholar.org/paper/An-Overview-of-Microsoft-Academic-Service-(MAS)-and-Sinha-Shen/b6b6d2504fd57d27a0467654fa62169cc7dedbdd?navId=citing-papers.
75. Pitts M, Savvana S, Roy SB, Mandava V. ALIAS: Author Disambiguation in Microsoft Academic Search Engine Dataset. In: EDBT; 2014. .
76. Academic M, How Microsoft Academic uses knowledge to address the problem of conflation/disambiguation; 2018. /https://www.microsoft.com/en-us/research/project/academic/articles/microsoft-academic-uses-knowledge-address-problem-conflation-disambiguation/ (Accessed: 21-January-2019).
77. Tang J, Zhang J, Yao L, Li JZ, Zhang L, Su Z. ArnetMiner: extraction and mining of academic social networks. In: KDD; 2008. .
78. Butler D. Free journal-ranking tool enters citation market. Nature 2008;451:6–6.
79. Scimago Journal & Country Rank; (Accessed: 14-February-2019). /https://www.scimagojr.com/journalrank.php.
80. Pycld2 - Python Package; (Accessed: 14-February-2019). /https://pypi.org/project/pycld2/.
81. Colavizza G, Franssen T, van Leeuwen T. An empirical investigation of the Tribes and their Territories: are research specialisms rural and urban? Journal of Informetrics 2019;13(1):105–117.
82. Nature Editorial Criteria and Processes.; (Accessed: 15-July-2018). /https://www.nature.com/nature/for-authors/editorial-criteria-and-processes.
83. Low Y, Gonzalez J, Kyrola A, Bickson D, Guestrin C, Hellerstein JM. GraphLab: A New Framework For Parallel Machine Learning. In: UAI; 2010. .
84. MongoDB; (Accessed: 14-February-2019). /http://www.mongodb.com.

Supplementary Materials – Additional Results

Additional Results

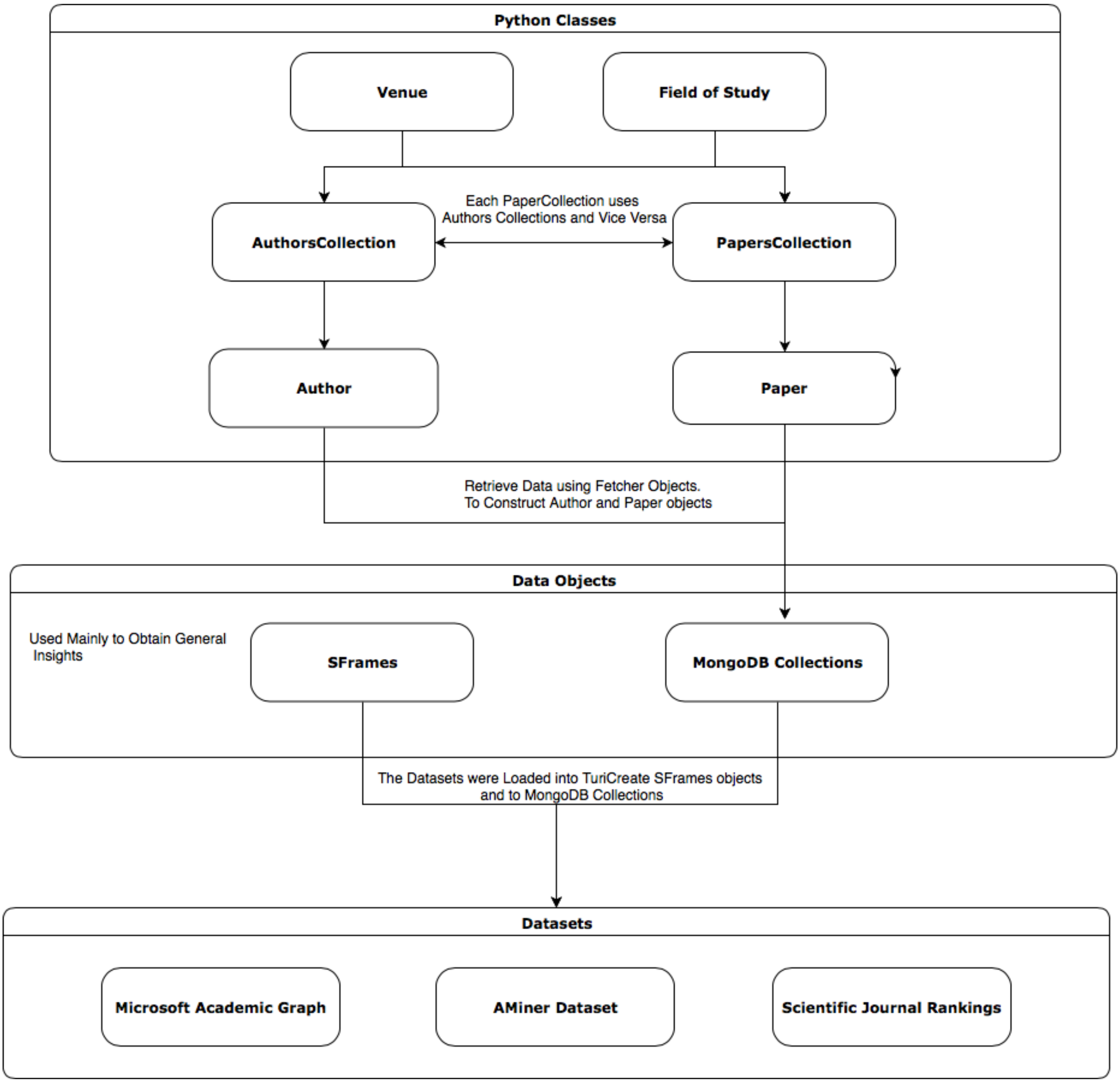

Figure S1. Overview of the Code Framework. The datasets are loaded into SFrame objects and MongoDB collections. The SFrame objects are used mainly to obtain general insights by analyzing tens of millions of papers and author records. The MongoDB collections are used to construct Paper and Author objects that can be used to analyze more complicated statistics for specific venues and research fields with usually hundreds of thousands of records.

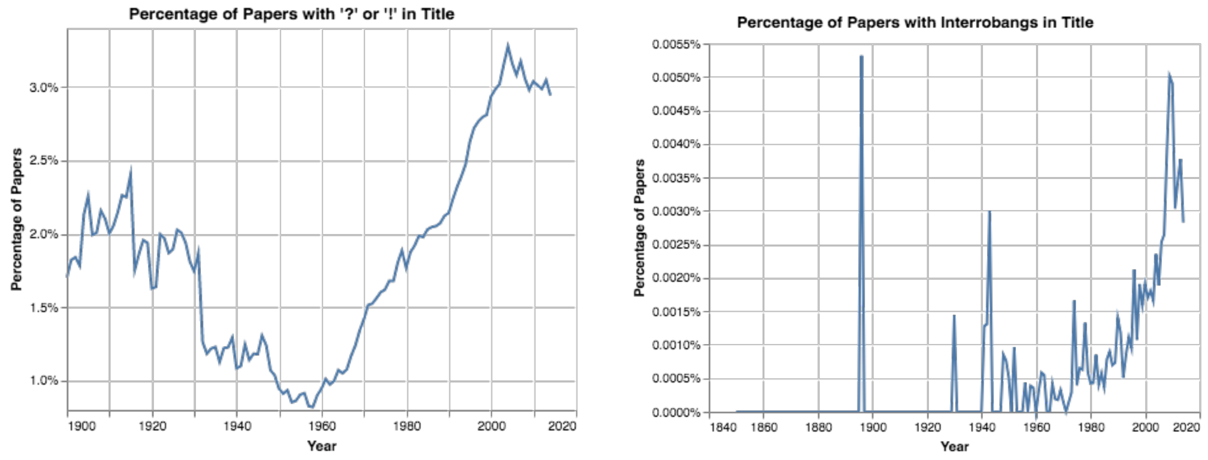

Figure S2. Percentage of Titles with Question or Exclamation Marks. The percentage of papers with question or exclamation marks in their titles increased over time, as well as the percentage of titles with interrobangs (represented by ?! or !?).

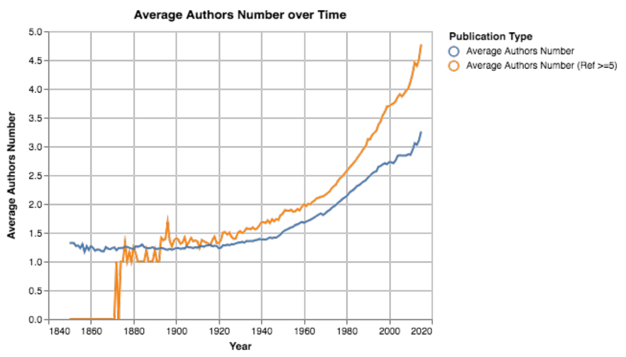

Figure S3. Average Number of Authors over Time. There has been a rise in the average number of authors, especially in recent decades.

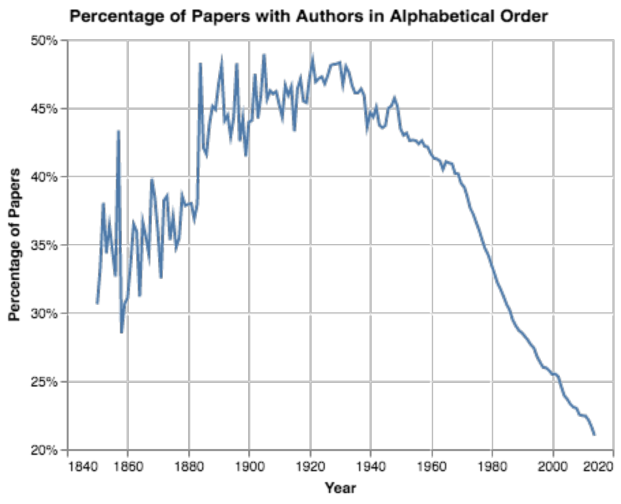

Figure S5. Percentage of Author Lists in Alphabetical Order. There has been a decline in the number of author lists organized in alphabetical order.

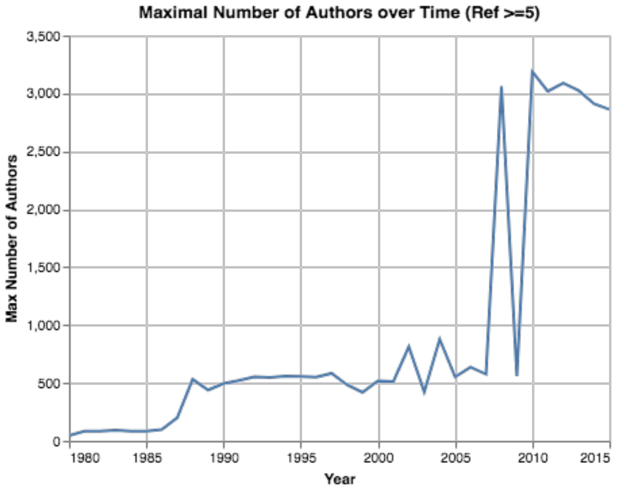

Figure S4. Maximal Number of Authors over Time. In recent years the maximal number of authors per paper increased sharply from 520 authors in 2000 to over 3100 authors in 2010.

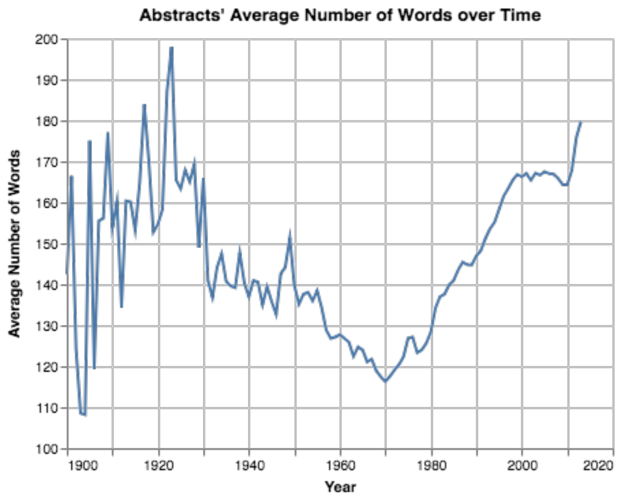

Figure S6. Average Length of Abstracts. Since 1970 there has been an increase in abstracts' average number of words.

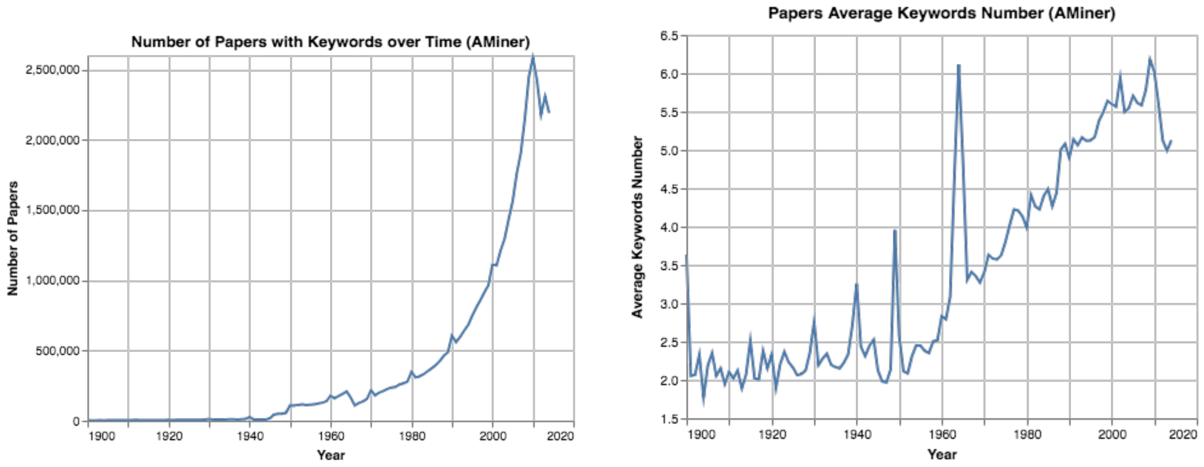

Figure S7. Keyword Trends. Both the number of papers with keywords has increased, as well as the average number of keywords per paper.

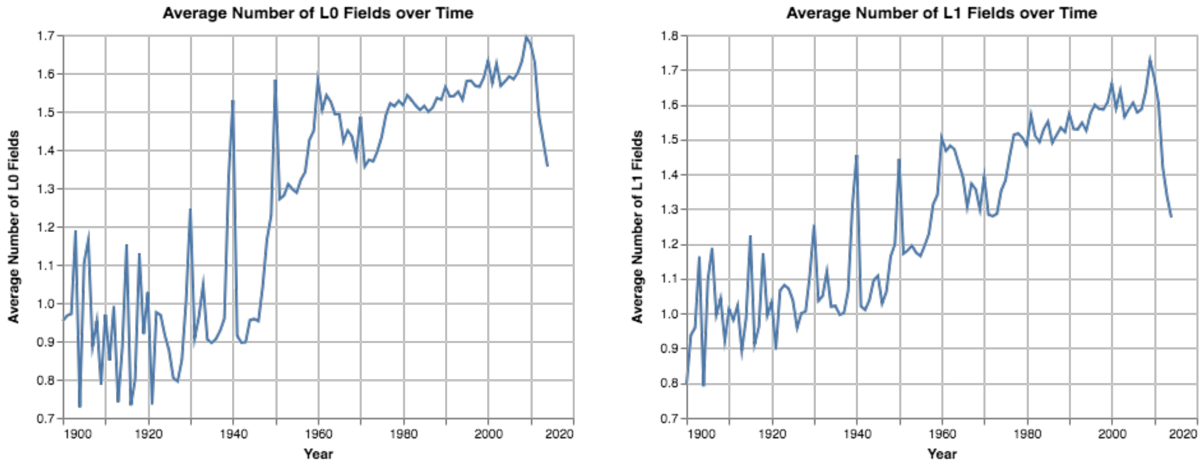

Figure S8. Average Number of Fields of Study over Time. Over time both the average number of L0 and L1 fields of studies per paper considerably increased. We believe the drop in the average number of L0 and L1 fields is a direct results of the drop in the number of papers with keywords in the same years (see the Results of Paper Trends) section.

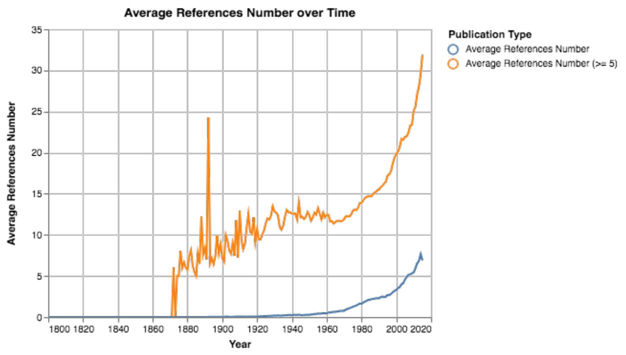

Figure S9. Average Number of References over Time. Over time, the average number of references sharply increased.

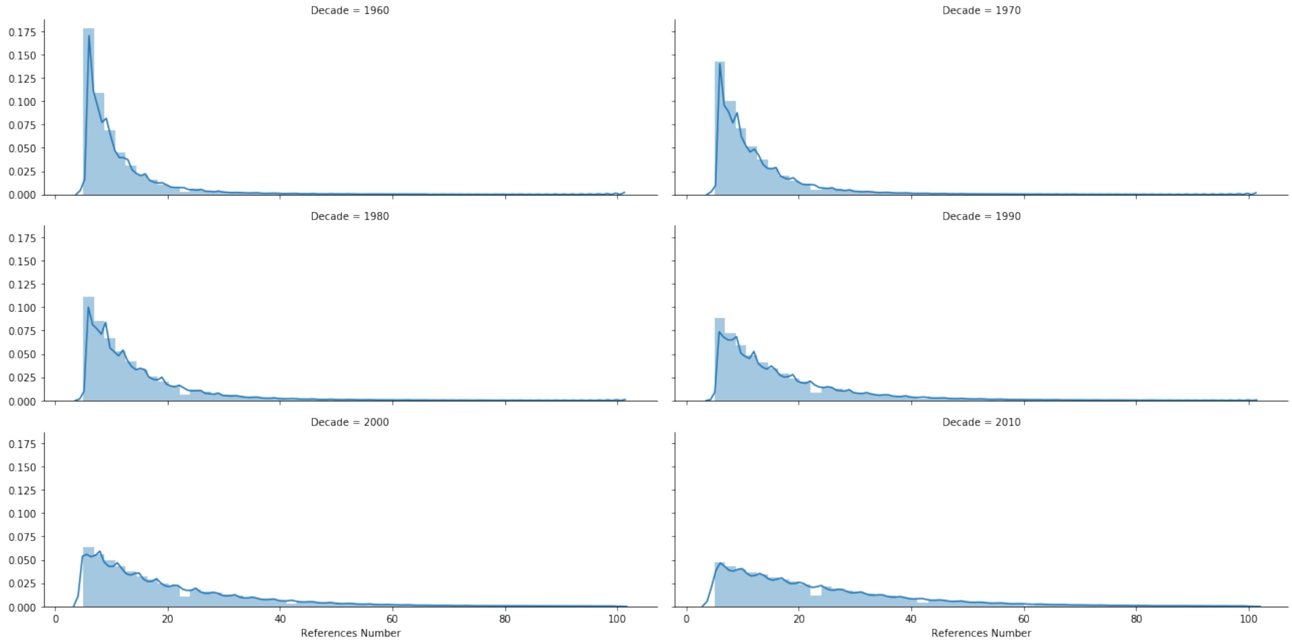

Figure S10. Distributions over Time of References in Papers. Over time, papers with a relatively high number of references have become more common.

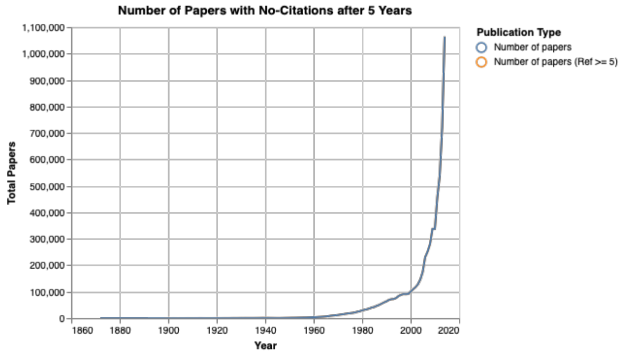

Figure S11. Total Number of Papers with No Citations after 5 Years. The number of papers with increased sharply over time.

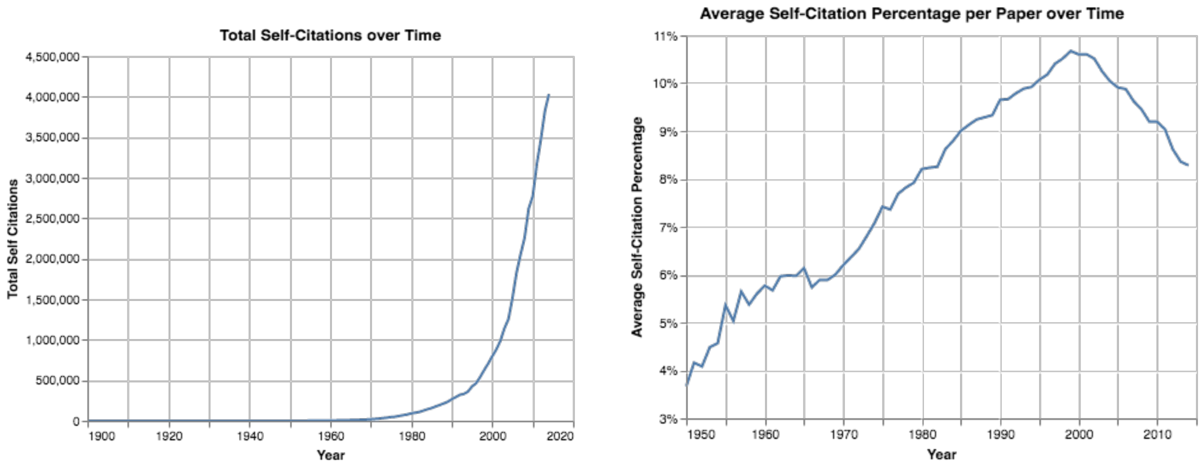

Figure S12. Total Number of Self-Citations and Percentage of Papers with Self-Citations. We can observe that over time both the total number of self-citations as well as the percentage of papers with self-citations increased significantly.

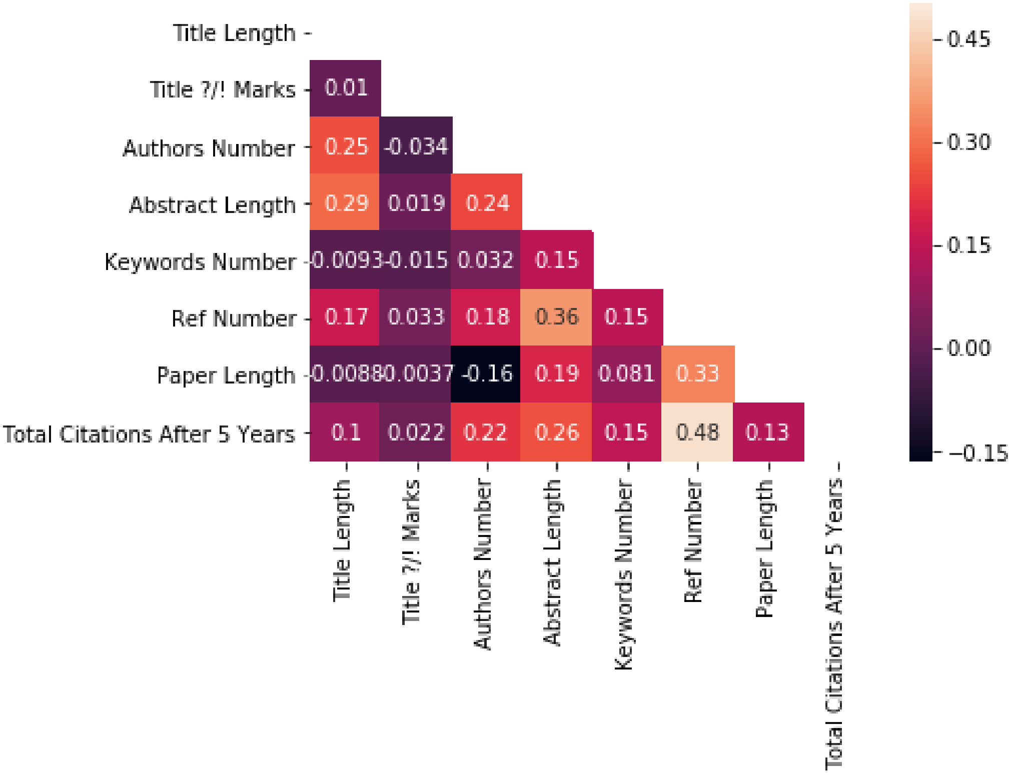

Figure S13. Spearman Correlation Heat Map for Papers' Properties. We can observe positive correlations among papers' various structural properties and the papers' total number of citations after 5 years.

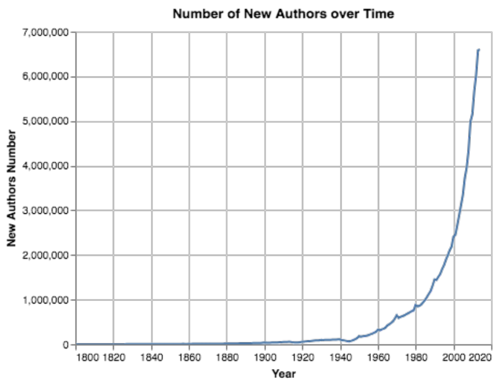

Figure S14. New Authors over Time. The number of authors, with unique MAG author IDs, who published their first paper each year.

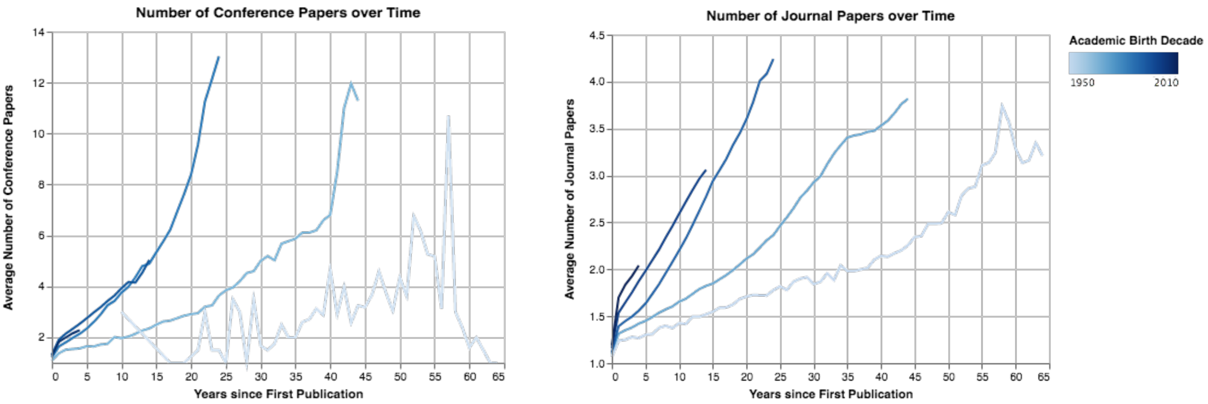

Figure S15. Authors Average Number of Conference and Journal Papers over Time. The average publication rate of both journal and conference papers increased with every decade.

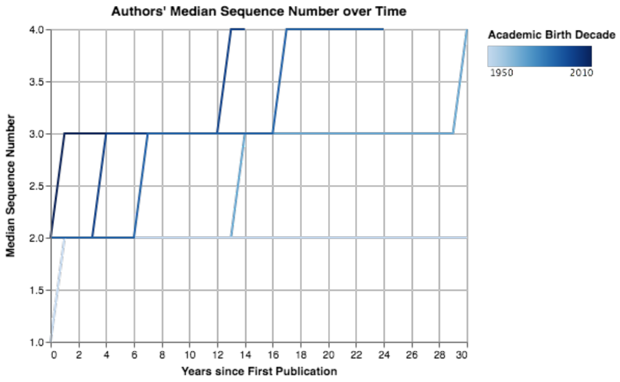

Figure S16. Authors' Median Sequence Number over Time. We can see that over time the median sequence numbers increased; i.e., senior researchers tend to have higher sequence numbers.

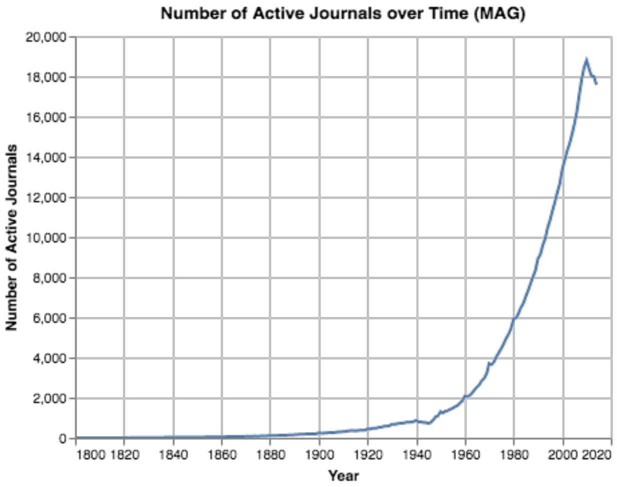

Figure S17. Number of Journals over Time according to the MAG Dataset. There has been a drastic increase in the number of journals since the 1960s.

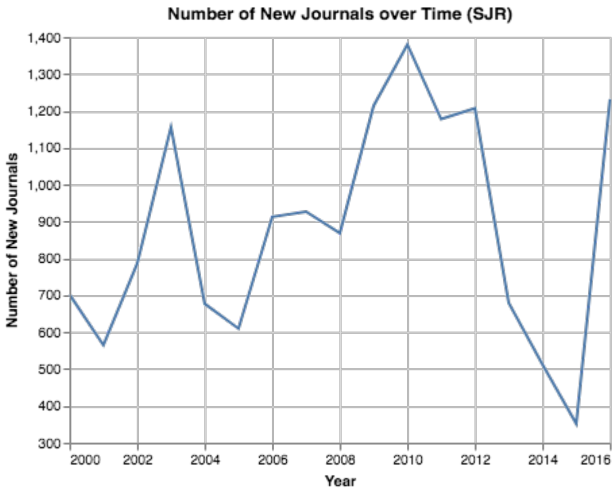

Figure S18. Number of New Journals by Year. Hundreds of new ranked journals are being published each year.

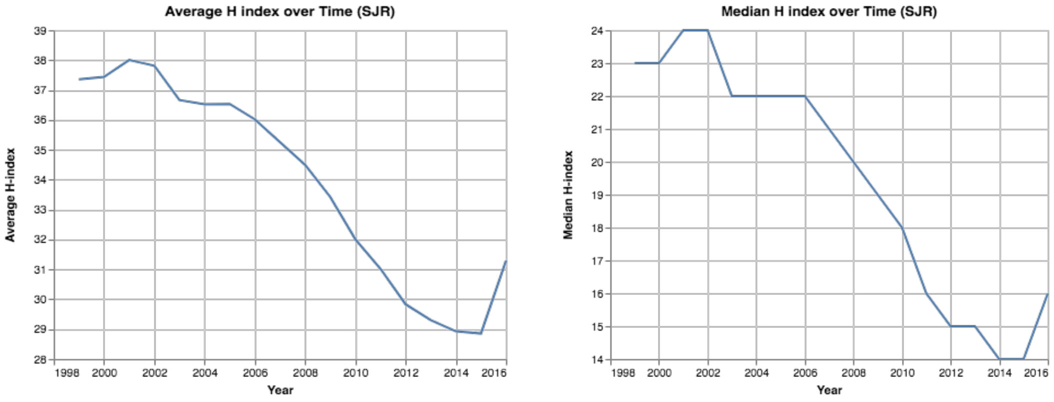

Figure S19. Journals' H-Index Average and Median Values. We can notice that over time both the average and median values of the journals' h-index measures decreased.

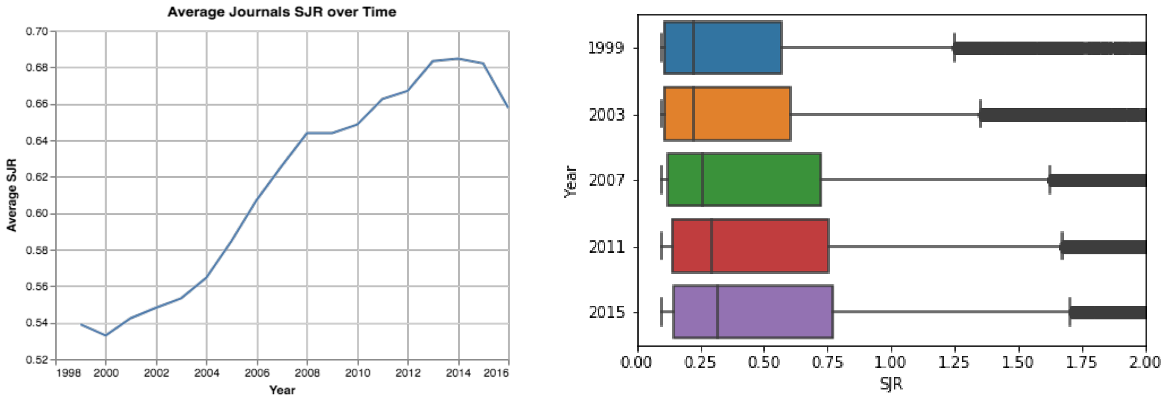

Figure S20. SJR Values over Time. We can observe that over time both the average and median SJR values increased.

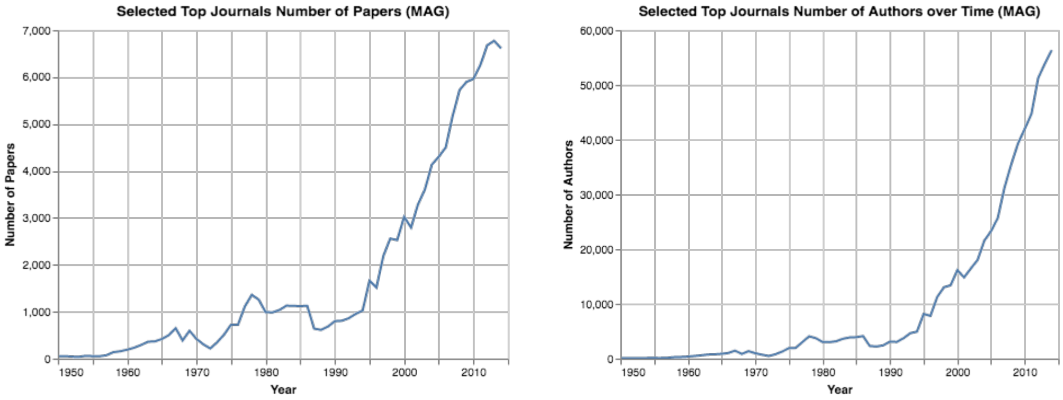

Figure S21. Top Journals' Number of Papers and Authors over Time. We can observe that both the number of papers and authors increased sharply in recent years.

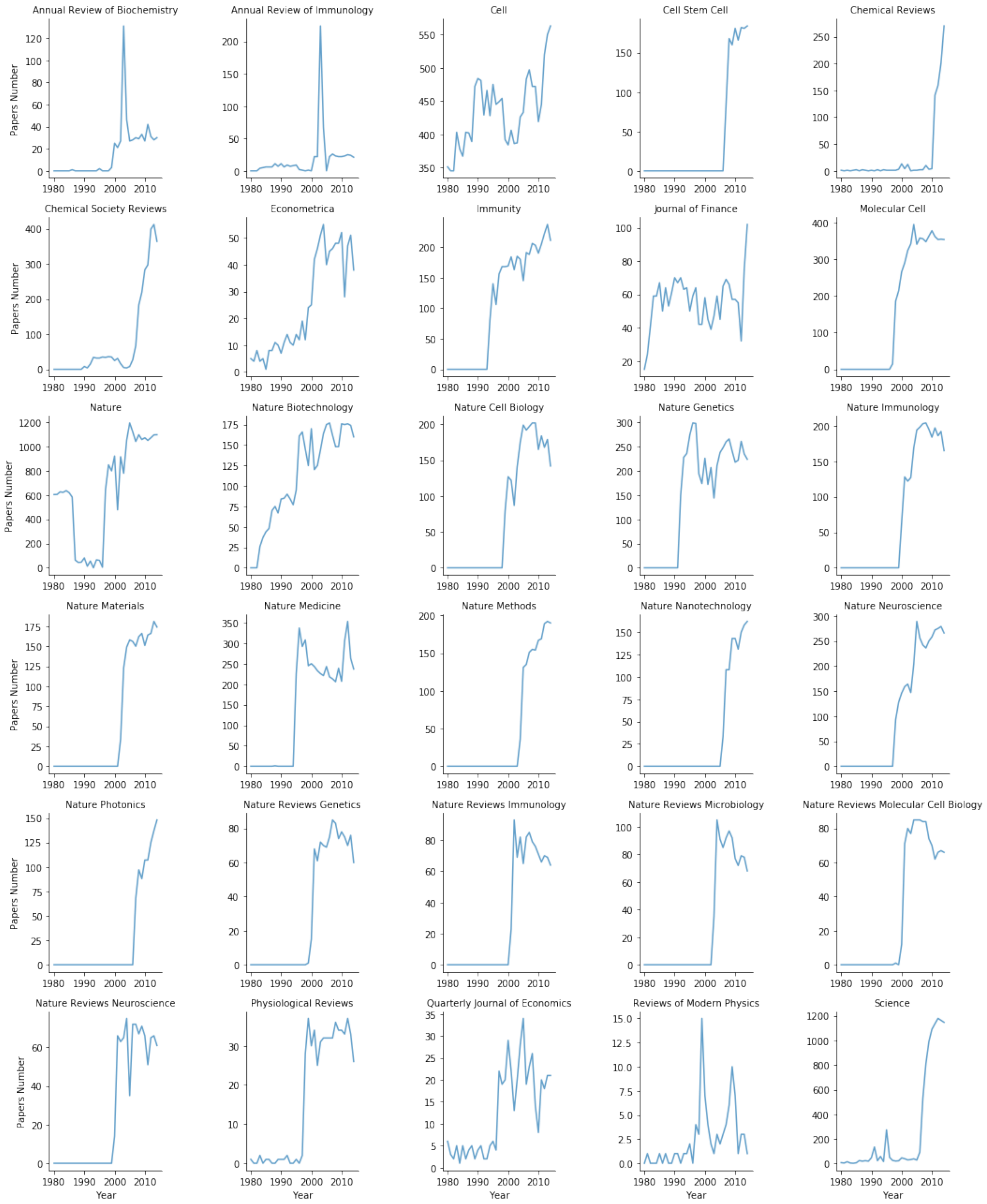

Figure S22. Top Selected Journals' Number of Papers over Time. It can be noted that in the vast majority of the selected journals the number of published papers with at least 5 references increased considerably over time.

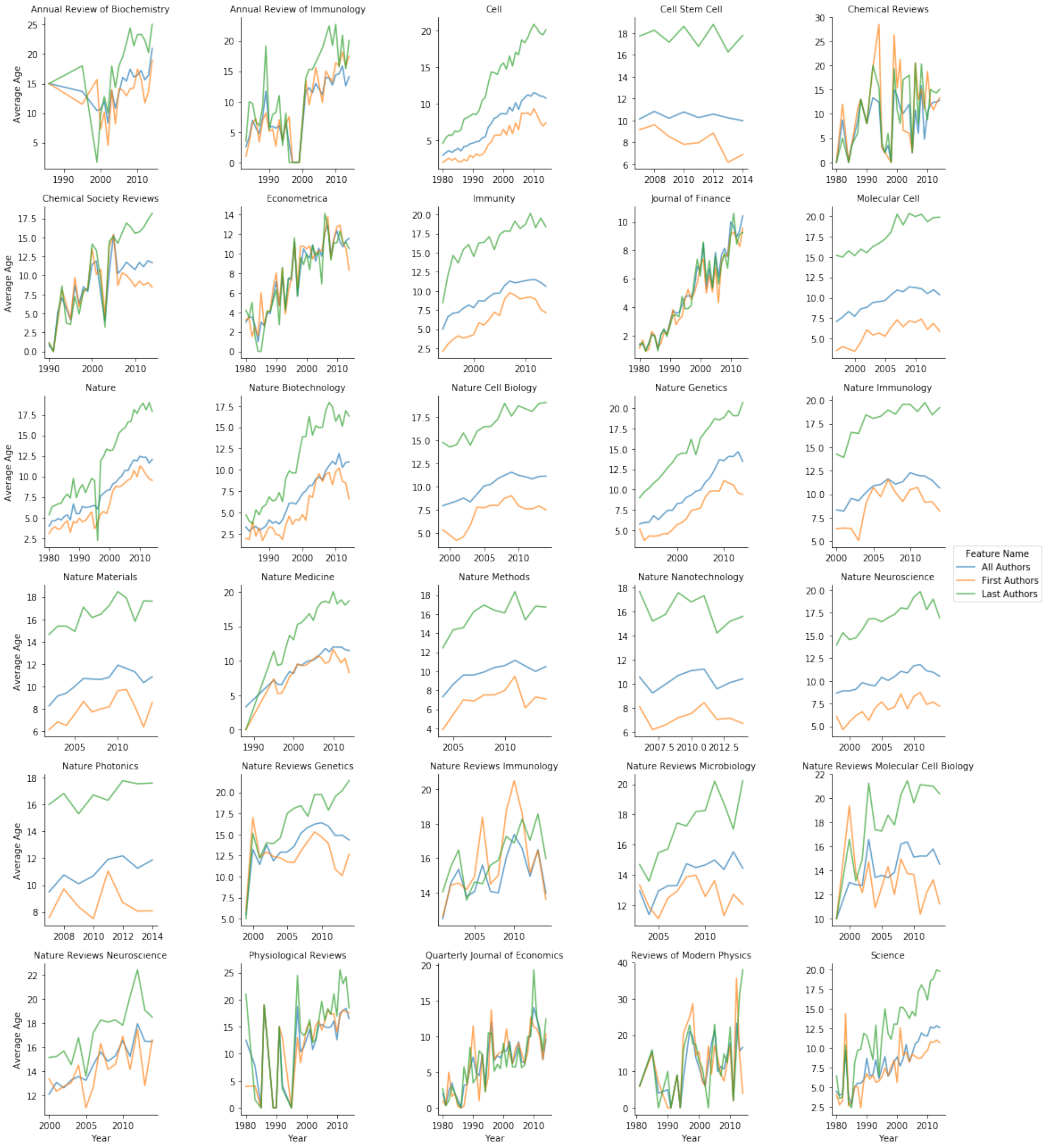

Figure S23. Top Selected Journals Average Author Career Age over Time. It can be noted that in the vast majority of the selected journals, the average age of authors, especially last authors, increased greatly over time.

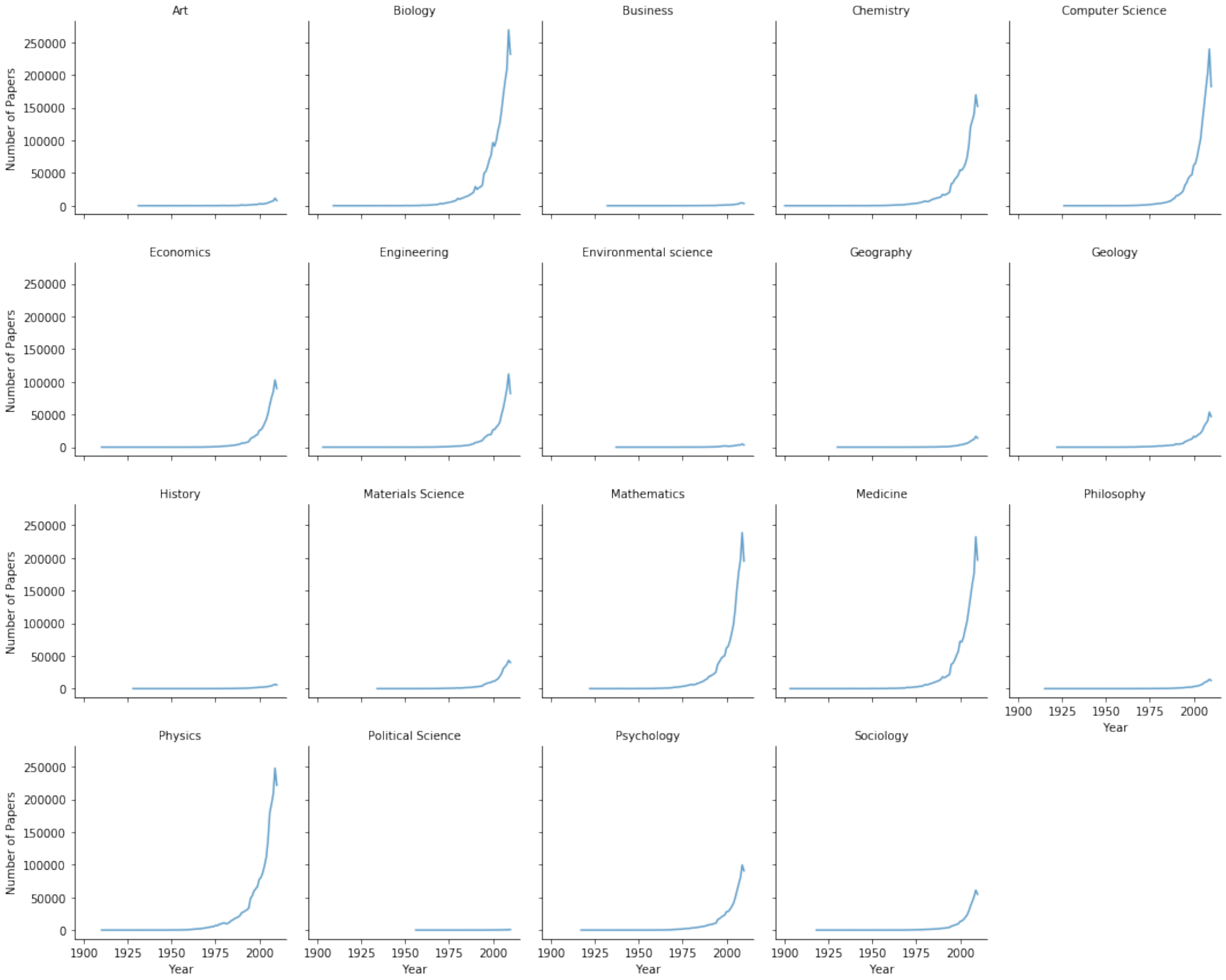

Figure S24. L0 Fields-of-Study Number of Papers over Time. We can observe the large diversity in the number of papers published in each L0 research field.

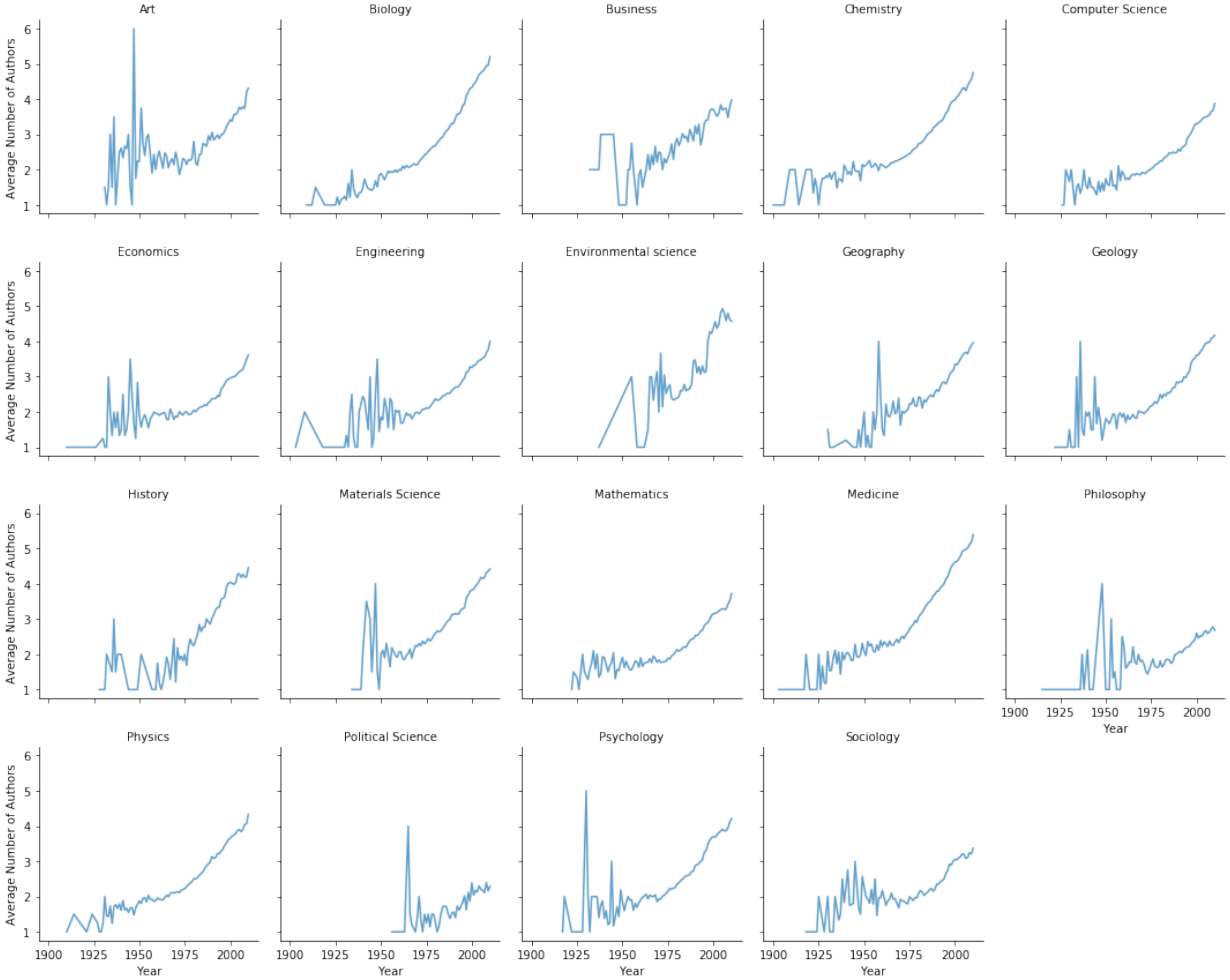

Figure S25. L0 Fields-of-Study Average Authors Number. We can observe a variation in the average number of authors across the various research fields.

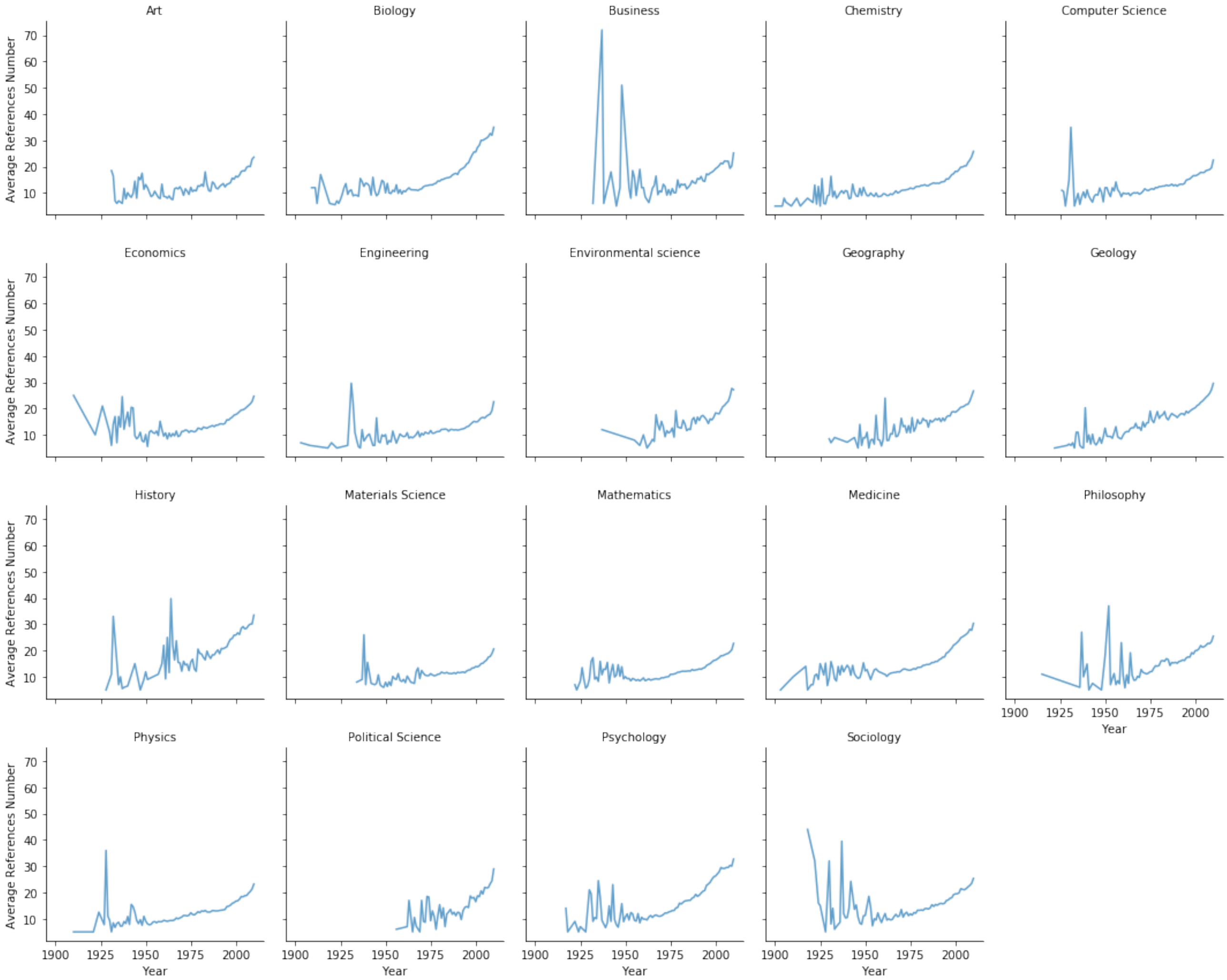

Figure S26. L0 Fields-of-Study Average References Numbers. We can observe variance among the reference numbers in different fields.

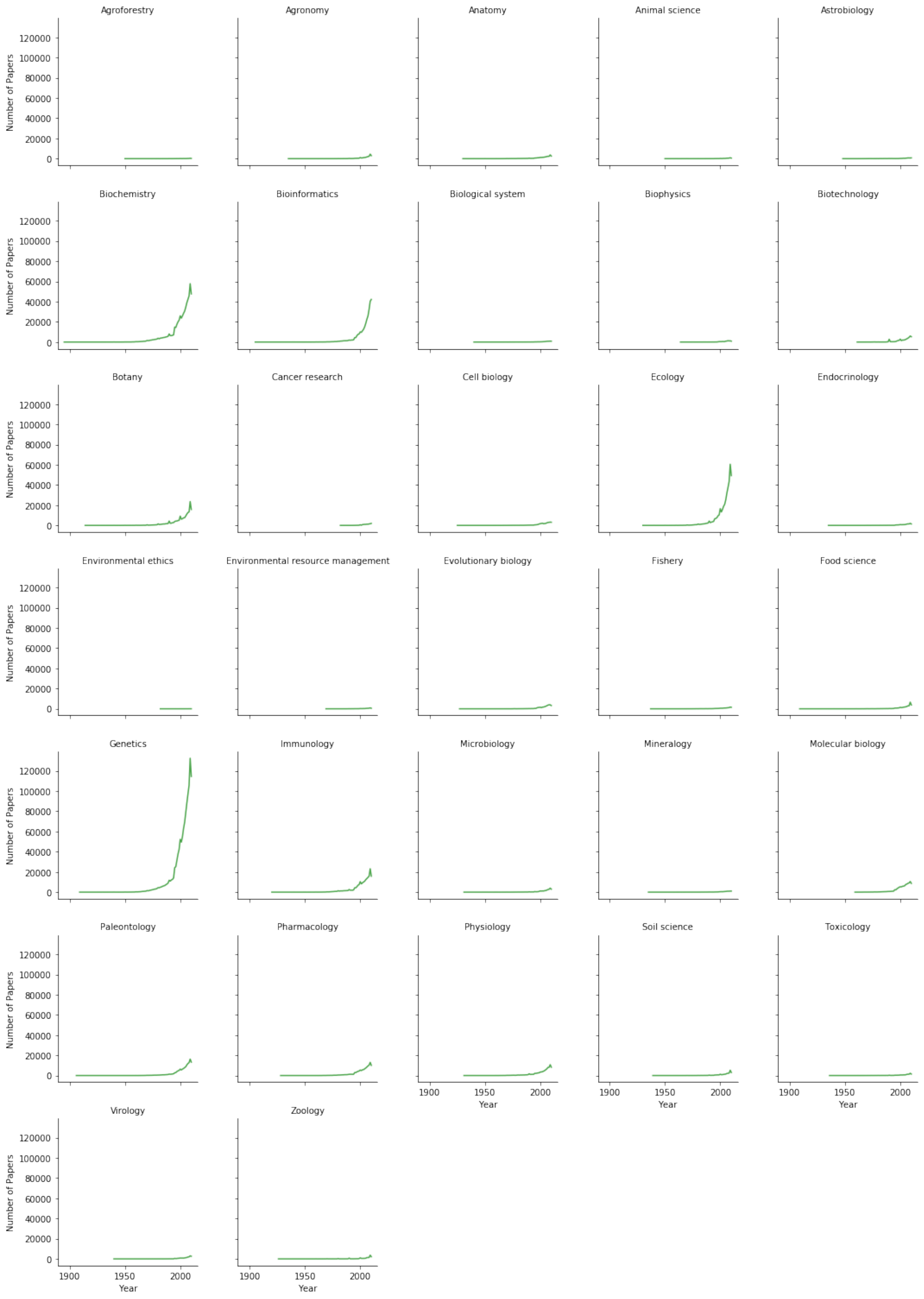

Figure S27. Biology L1-Subfields Number of Papers over Time. We can observe a big variance in the number of papers over time in the various biology subfields.

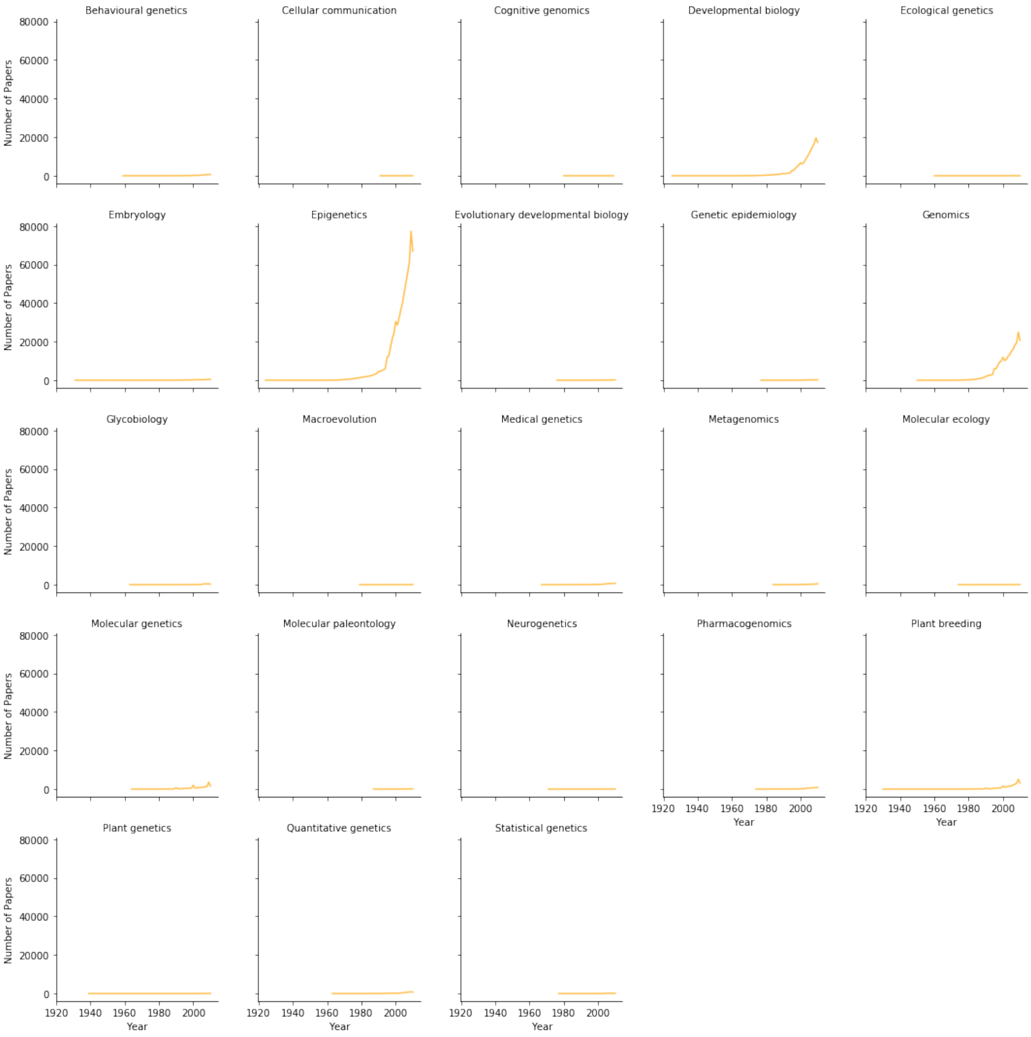

Figure S28. Genetics L2-Subfields Number of Papers over Time. We can observe a big variance in the number of papers over time in the various genetics subfields.

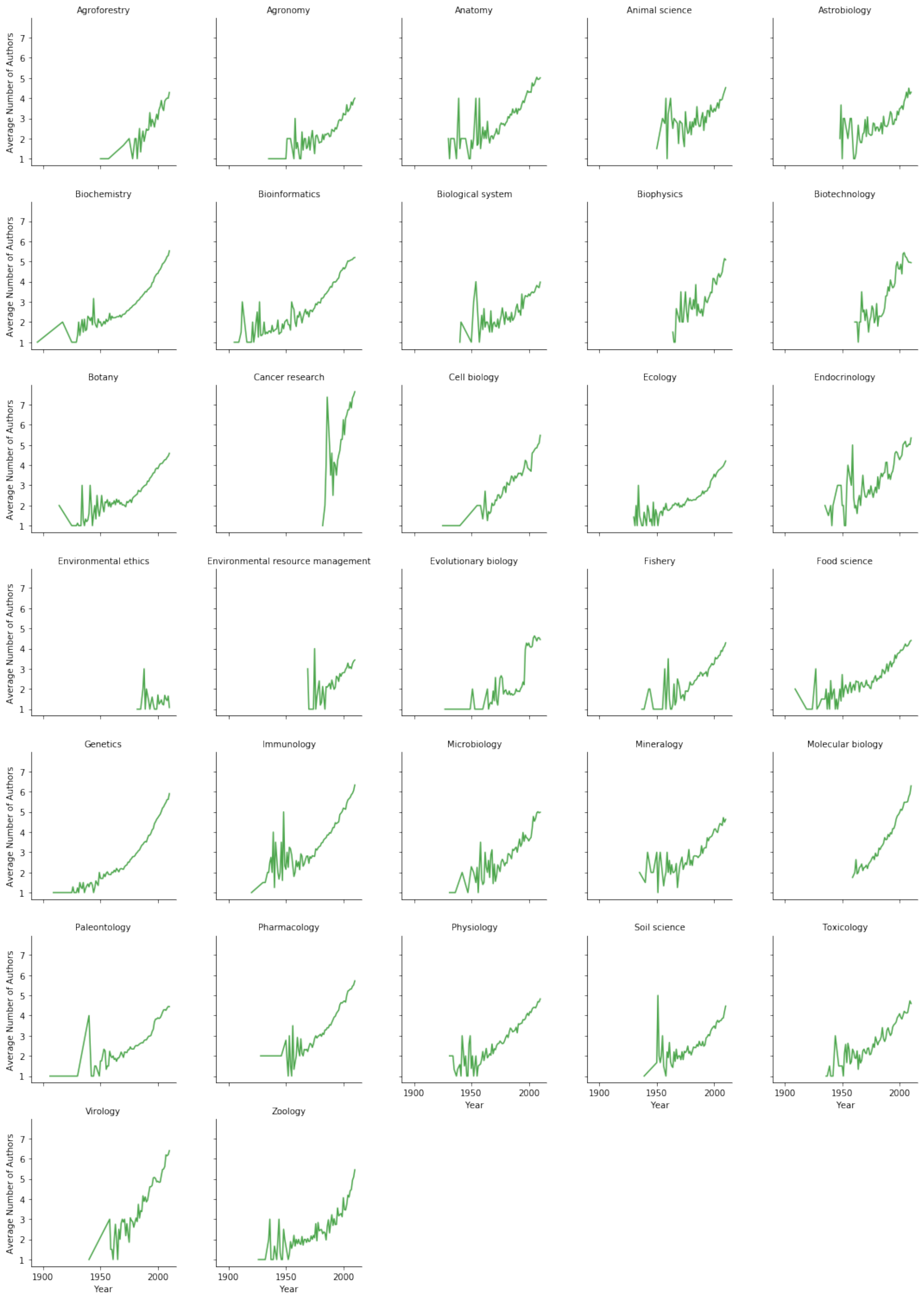

Figure S29. Biology L1-Subfields Average Number of Authors over Time. We can observe a variance in the average number of authors over time in the various biology subfields.

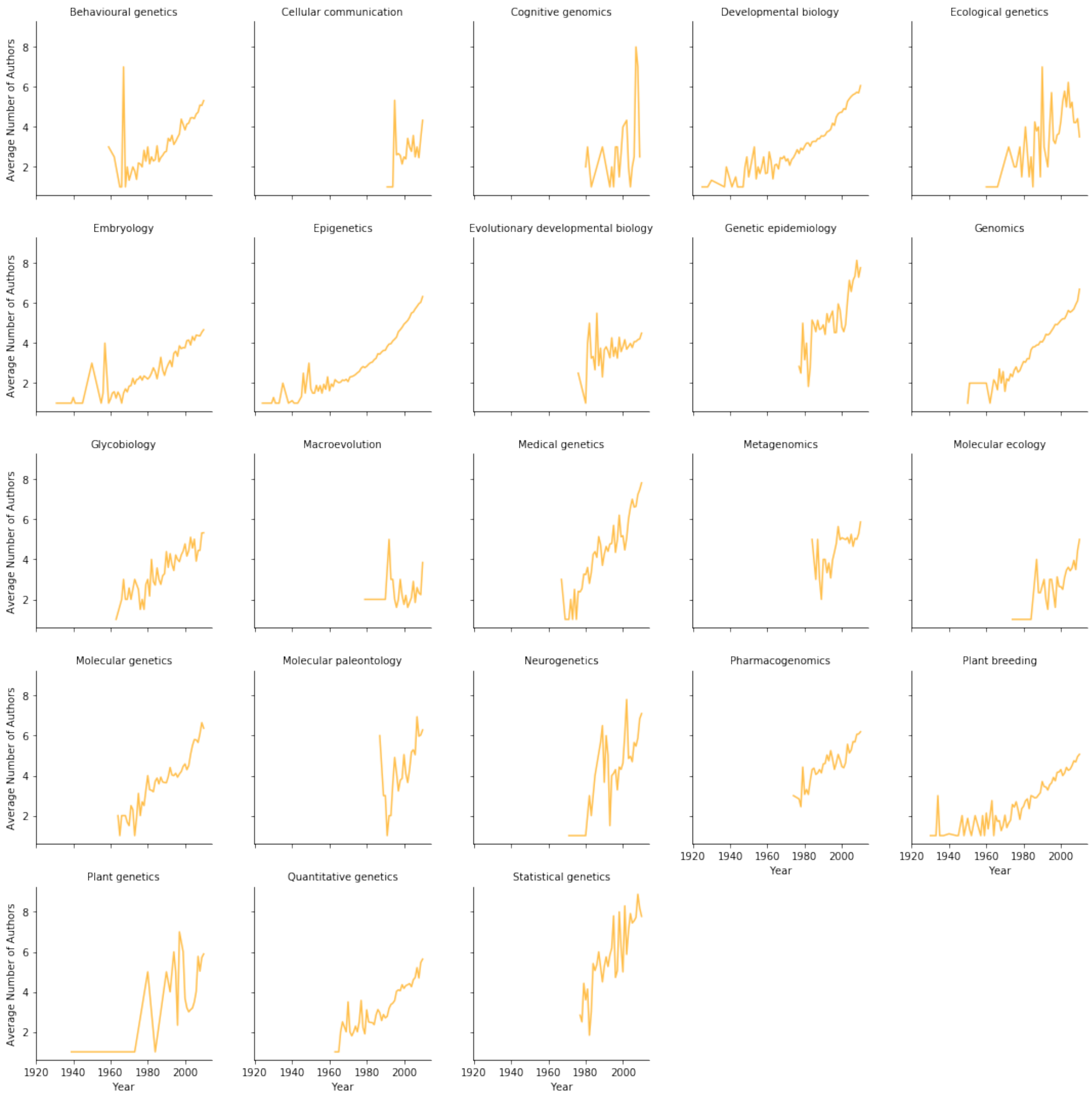

Figure S30. Genetics L3-Subfields Average Number of Authors over Time. We can observe a significant variance in the average number of authors over time in the various genetics subfields.

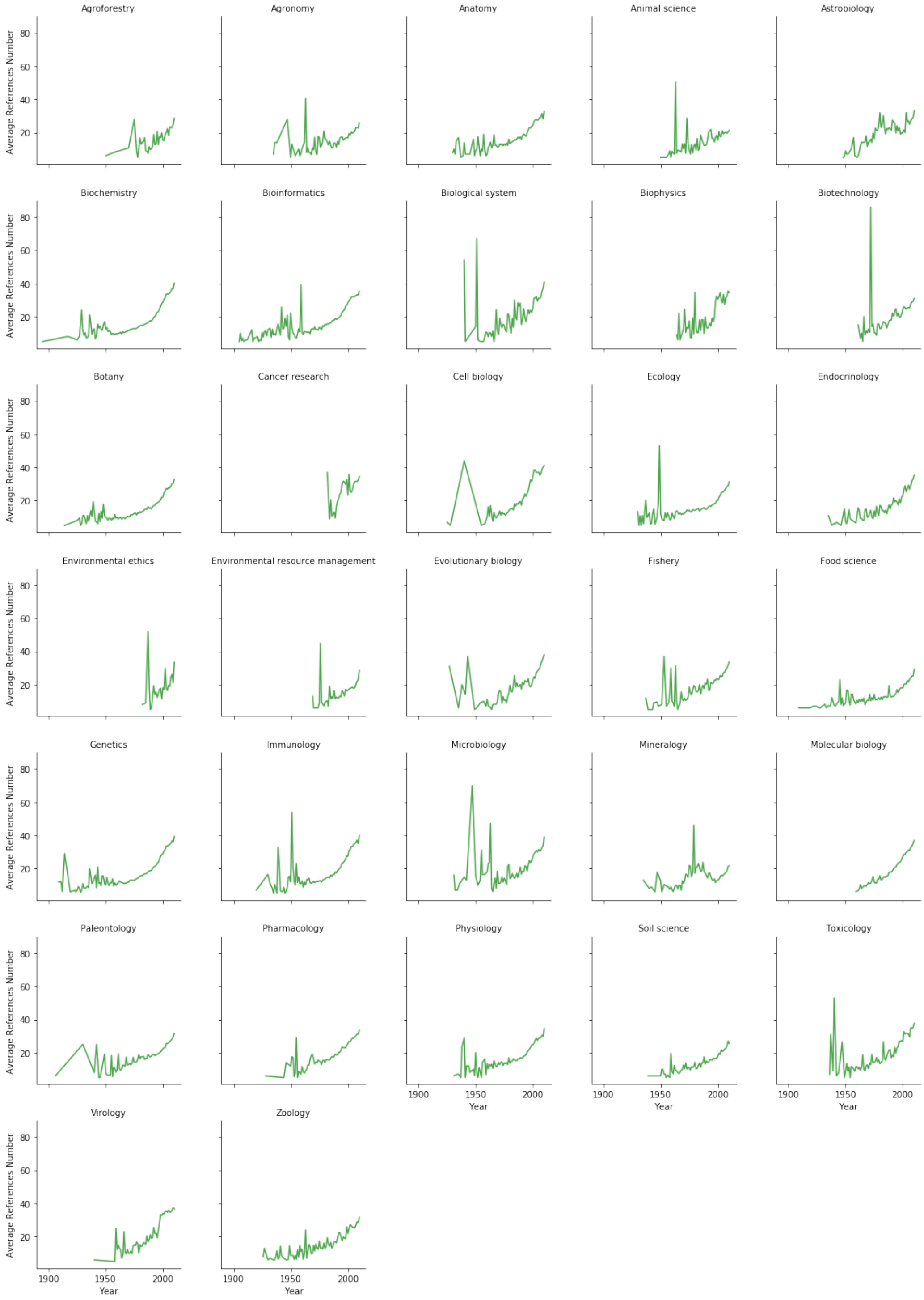

Figure S31. Biology L1-Subfields Average Number of References over Time. We can observe a variance in the average number of references over time in the various biology subfields.

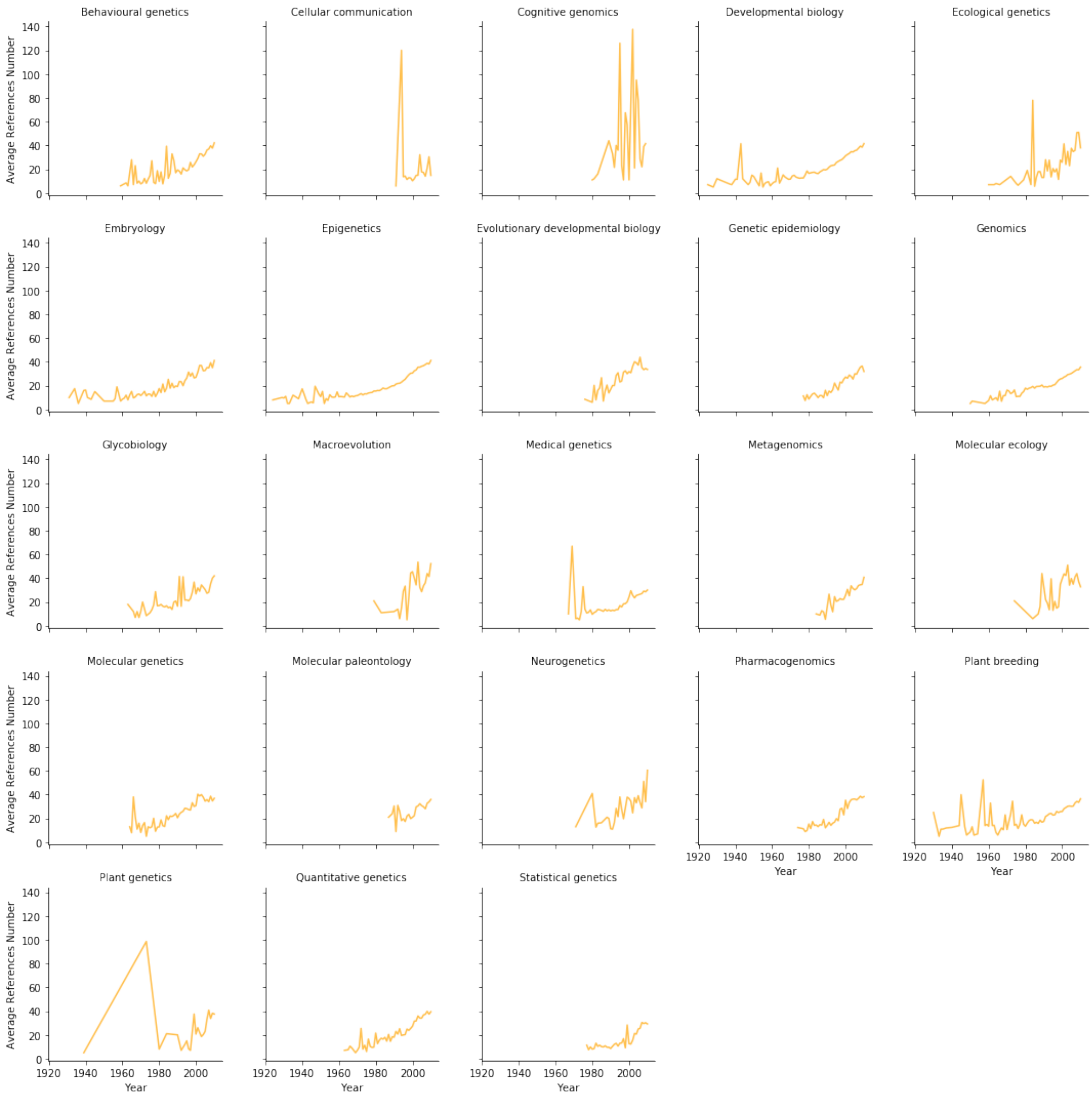

Figure S32. Genetics L2-Subfields Average Number of References over Time. We can observe a significant variance in the average number of references over time in the various genetics subfields.

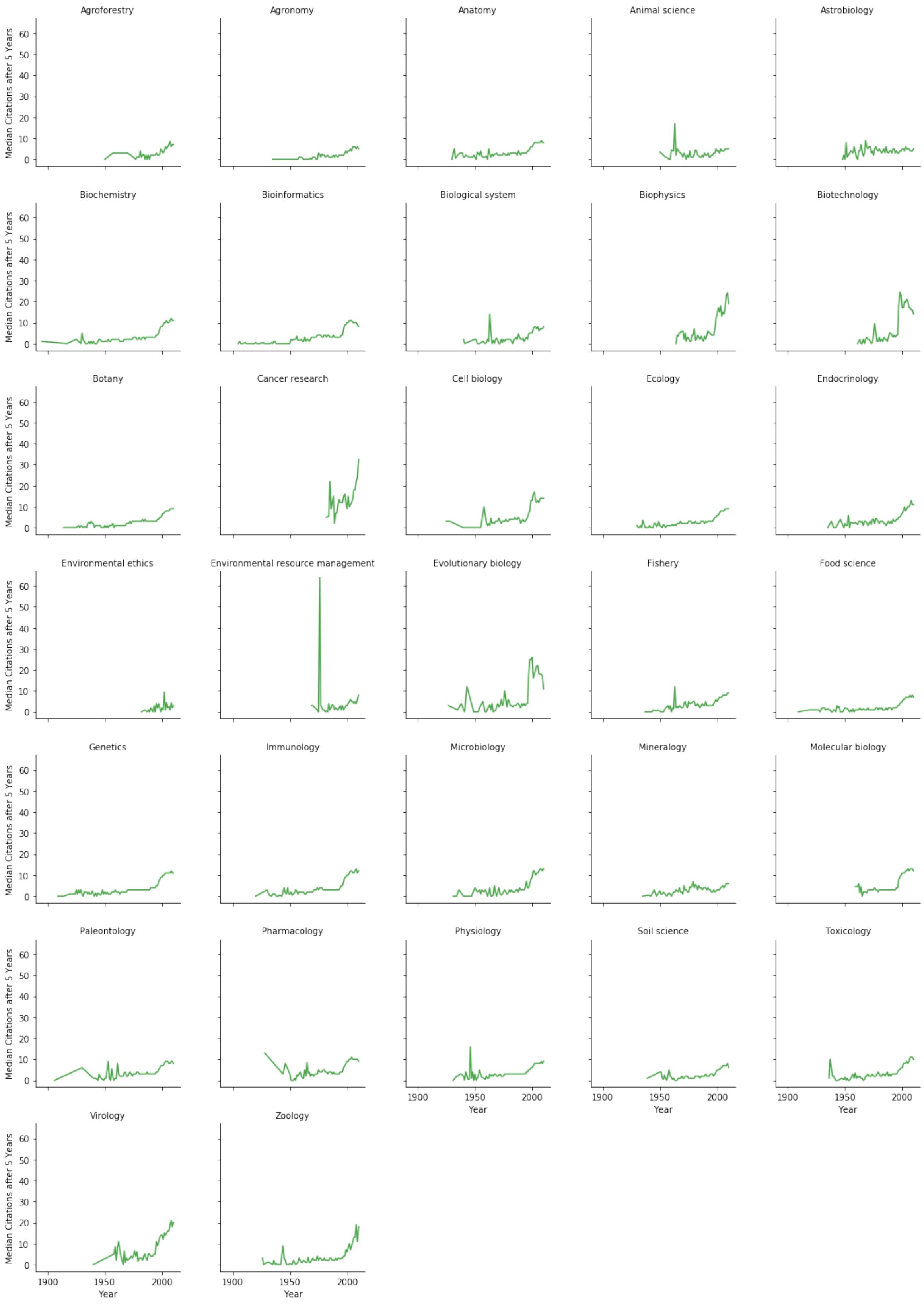

Figure S33. Biology L1-Subfields Median Number of 5-Year Citations over Time. We can observe a variance in the median number of citations over time in the various biology subfields.

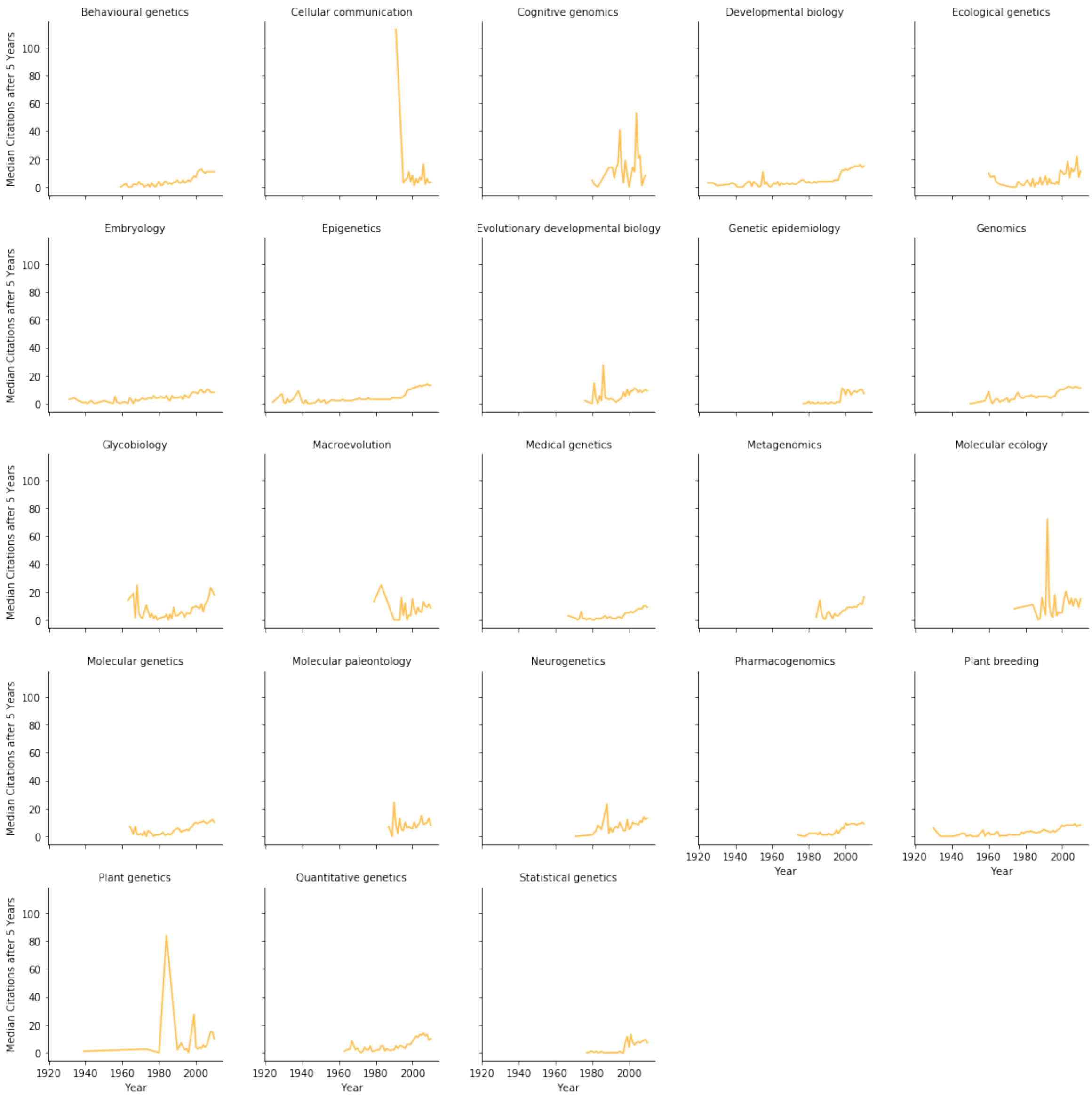

Figure S34. Genetics L2-Subfields Median Number of 5-Year Citations over Time. We can observe a significant variance in the median number of citations over time in the various genetics subfields.

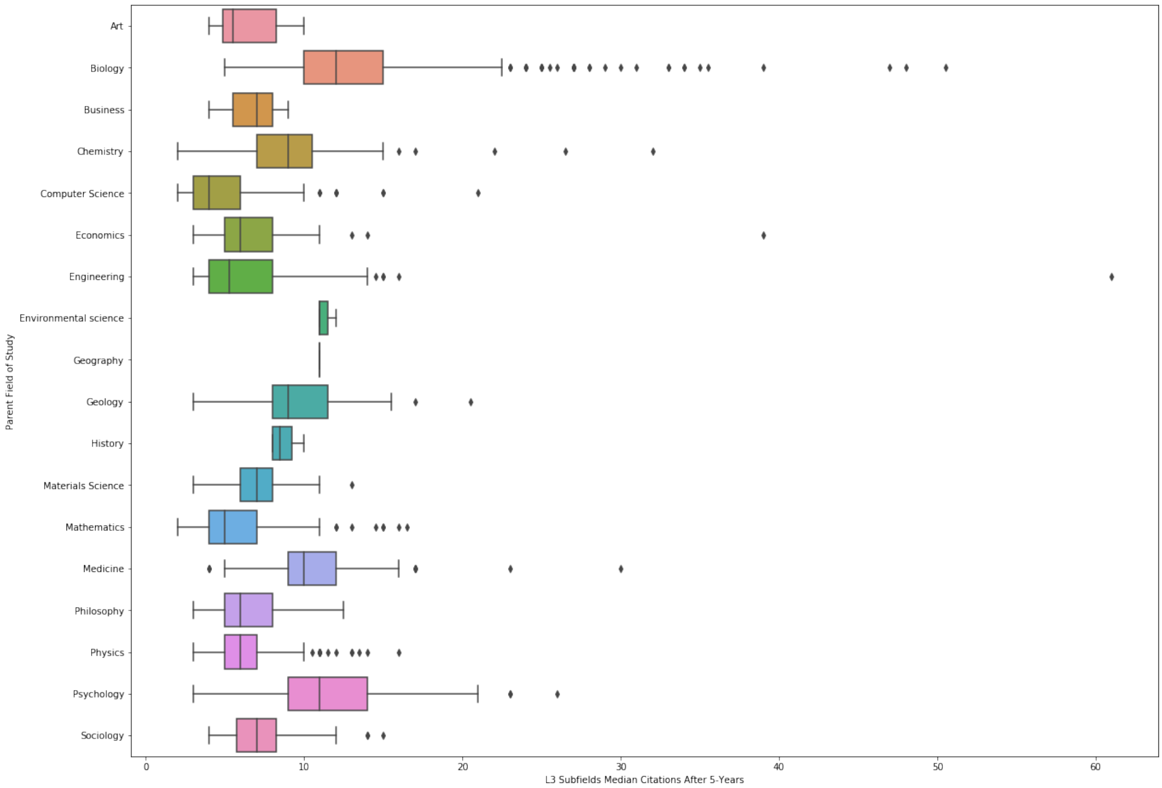

Figure S35. L3 Fields-of-Study Median 5-Year Citation Distributions by Parent Fields. We can observe the high variance among the L3 fields-of-study median citation numbers.

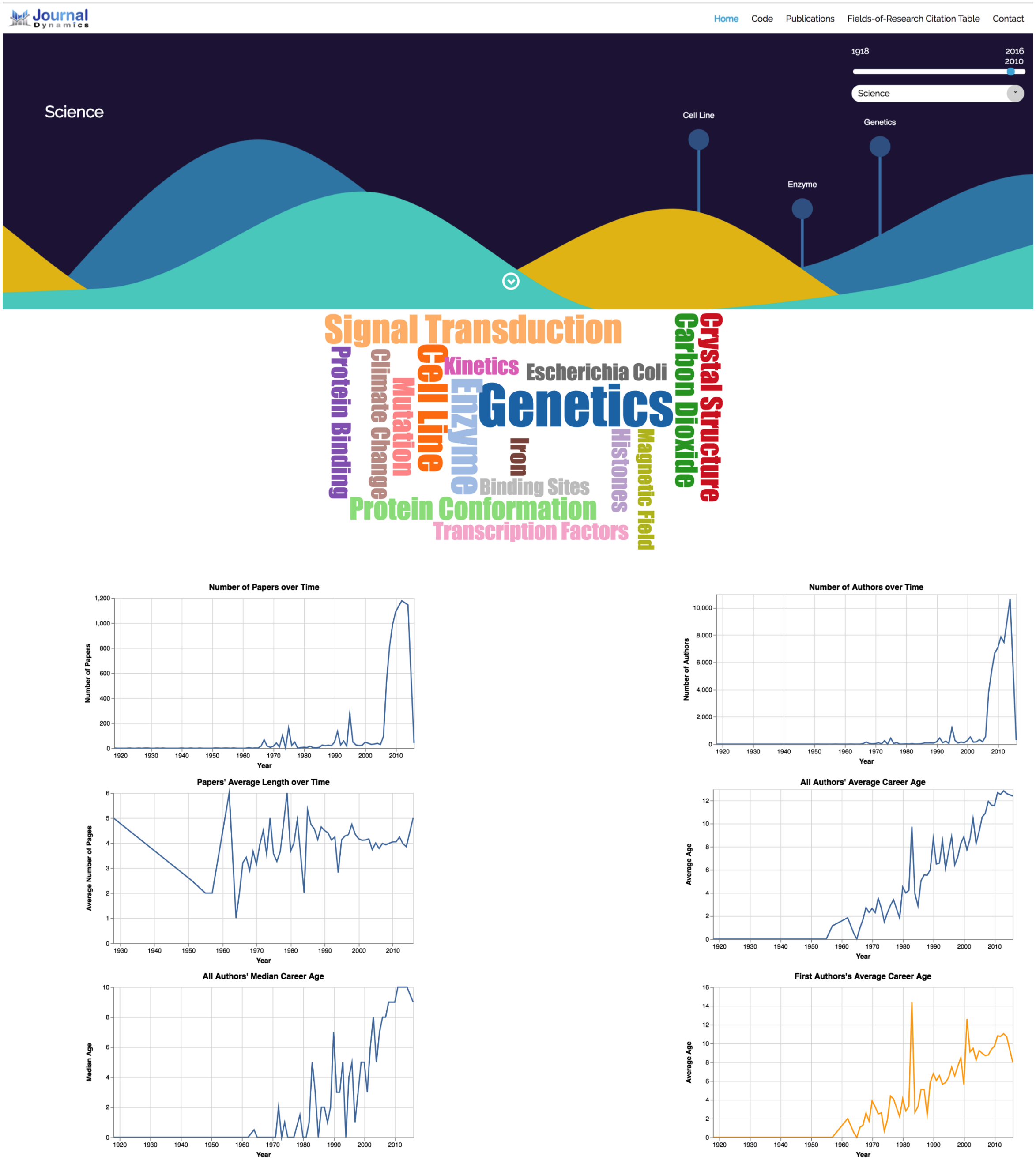

Figure S36. Interactive Website. We have developed an interactive website that makes it possible to view and interact directly with the study's data.

Click here to access/download;Personal Cover;reviewer_response_minor - final.pdf

"Over-Optimization of Academic Publishing Metrics: Observing Goodhart's Law in Action"

Response to Reviewers

We would like to thank the reviewers for their highly valuable and constructive criticism. The comments have been very helpful in the preparation of the revised manuscript. We have addressed the reviewers' concerns and have improved the article accordingly.

The following is a description of the revisions we have made in order to address the comments pointed out by the reviewers.

Reviewer 1:

Introduction

Comment 1: Figure 1 is very interesting, however, it appeared far too early in the paper. It makes the figure not very understandable (readers at this stage have no idea on how these data have been collected and analyzed). Maybe Figure 1 should be provided as a summary-of-results, later in the paper (in the discussion section?)

Response 1: We thank the reviewer for this comment. We have moved Figure 1 (now labeled as Figure 22) to the Discussion section.

Comment 2: A citation (or several citations if needed) from the literature would be sufficient to describe the exponential growth of academic publishing. Introduction shouldn't reports results from the present study (so Figures 14 and S17 shouldn't be mentioned in this section) - numbering of figures should also be checked in the entire article (Figure 14 shouldn't follow the Figure 1 in the order of apparition).

Response 2: As suggested, we have added citations to the relevant work for describing the exponential growth of academic publishing. In addition, we have removed the reference to figures from the current study. Furthermore, we have updated the numbering of the figures so they are ordered correctly.

Comment 3: Again in the Introduction section, paragraphs Papers, Authors, Journals and Fields of Research are in fact summary of results. It should be reported later in the paper.
The introduction section should present hypotheses that were formulated before analyses were performed.
Paragraphs "These observations support the hypothesis []... (see the Results of Paper Trends section and Figure S13)" and
"It is time to consider [...] academic publishing world" are in fact discussion paragraphs.
There is a need in the introduction section to formulate the general objective of the paper.

Response 3: We have revised the Introduction section according to these helpful comments. The paragraphs related to results have been removed, as have those that fit better in the Discussion section. Moreover, we have inserted a paragraph to make the general objective of the paper very clear.

Background

Comment 4: The sentence "In this section, we give a short overview of the relevant scientometric papers to this study" is unclear. Suggestion: "In this section, we present studies that analyze changes in academic publications in recent years ..."

Response 4: In the revised manuscript, we have changed the text according to the above suggestion.

Comment 5: Paragraph " Our study is greatly influenced by a recent study by [...](and hence the status) of the research." would be better in the introduction section.

Response 5: We agree with the reviewer's comment, and we have moved this paragraph to the Introduction section.

Data Description

Comment 6: DOI is a good way of identifying an article, but the "unique author ID value" is not very clearly explained.

Response 6: One of the interesting challenges in analyzing bibliometric datasets is solving the author disambiguation problem. In other words, in order to calculate various statistics, it would very helpful to know which papers were written by the same author. The problem is that in many cases matching an author to a paper can be extremely challenging. For example, researchers can change their last names, affiliations, and even their research domains. If a researcher published only few papers under one name and then changed his/her name, it is very difficult to match the old papers to the new name. Moreover, there are some names that are extremely common. To tackle this challenge, the MAG dataset uses an author disambiguation algorithm and sets a unique author ID value to each author identified by the algorithm. Recently, Microsoft Academic released a post that explains how they address the problem of conflation/disambiguation.

In the revised manuscript, we have elaborated on the unique author ID value and added relevant references.

Comment 7: it is quite uncommon to use these datasets for scientometric purposes (if not, please provide examples of such previous use).
Authors should better explain why they use these datasets instead of more traditional databases (e.g, for biomedical research, scopus, embase, medline, psychinfo etc). They should also explain how fields of research are integrated into these datasets, how complete they are, how representative of the literature they are.
Main comment of this reviewing: More precisions on datasets that have been used are very important to assess external validity of the present analyses (are the references included in these datasets representative of the overall knowledge?).**:**

Response 7: In recent years with the significant advantages of data science tools, the availability of big-scale datasets, and the advancements in cloud computing, it has finally become possible for researchers to analyze big datasets, such as MAG and AMiner. For example, about six years ago when we needed to analyze a large-scale dataset, we required a strong Hadoop cluster with dozens and even hundreds of nodes (in one case we used thousands of nodes). For this study, we could simply use a strong cloud instance with 1-2TB of RAM and dozens of virtual CPUs.

While traditional scientometric datasets, such as Scopus, Mendeley, Medline, PsychINFO, etc., are useful for scientometric research, they are usually limited to specific domains and time. This limits the ability to observe global trends, such as those presented throughout this study. Moreover, the MAG dataset has additional benefits, such as author disambiguation and mapping papers to topics. In fact, the use of the MAG dataset for scientometrics has gained increasing popularity in recent years. Moreover, a recent study by Herrmannova and Knoth [1] describes in detail the properties of the MAG dataset, including various statistics like the number of papers in each field of study and the comparison of the dataset to other scientometric datasets, such Mendeley. For example, according to Herrmannova and Knoth's study, the MAG dataset contains nearly 15 million papers in the field of biology, while the Mendeley dataset contains fewer than 300,000 biological science papers [1].

While MAG is a great tool for scientometric research, the MAG dataset didn't contain all the paper features we required for this research. Therefore, we utilized the AMiner dataset to add additional features and to compare results with those obtained using the MAG dataset in order to validate the existence of observed patterns in both datasets. The AMiner is indeed a relatively new dataset, and we are among the first to use it for a scientometric study.

In the revised manuscript, we have elaborated on the MAG dataset and its increasing popularity. We also have added a reference to Herrmannova and Knoth's paper [1].

Comment 8: Authors should consider to better explain how Q1, Q2, Q3 and Q4 are defined in the SCImago journal rank dataset.

Response 8: In the revised manuscript, we have elaborated on the quartile definition and use.

Comment 9: Authors should also better describe the L0 to L3 classification: on which value is based the hierarchy ranking?

Response 9: In the revised manuscript, we have further developed our explanation of the field-of-study classifications. Additionally, we have added a reference to the Herrmannova and Knoth study [1], which contains an in-depth analysis of the various fields of study in the MAG dataset.

Analyses

Comment 10: Authors should better explain how they deal with non-English papers (since a specific analysis on languages appears in the beginning of the Results section).

Response 10: We thank the reviewer for this comment. In order to detect paper language, we utilized the pycld2 python package, which can identify a text language. The main advantage of using pycld2 is its speed, which is critical for analyzing over 100 million titles and abstracts, and we also appreciate its ease of use (one line of code).

In the revised manuscript, we elaborate on how we use pycld2 for language detection. Moreover, in the code section of the project's website there are more details on the creation of each result, including identifying non-English papers and presenting additional results regarding publication trends of non-English papers.

Comment 11: Y axis of Fig 5 should be labelled.

Response 11: We have added a Y-axis label to Figure 5.

Comment 12: The analysis of the total number of papers with no citations (Fig 9) should be presented using proportion data (%), so Fig S11 should be preferred to Fig 9 in the main text (+ there is a typo in the title of Fig 9 "aftetr"). Presenting a crude increase is not very useful, given the overall growth of yearly number of publications.

Response 12: As recommended, in the revised manuscript, we have swapped Figure S11 with Figure 9 and fixed the typo.

Results of Author Trends

Comment 13: A global information on how many unique author ID have been identified would be important. Footnote number 10 should be quantified: what is the proportion of unique authors with several IDs?

Response 13: We have mentioned the number of unique author IDs in the Data Description section (22.4 million authors with a unique author ID). Unfortunately, there are no available data for the performance of the author disambiguation algorithm used in the MAG dataset. Therefore, we aren't able to add an estimation of the number of authors with several IDs.

Results of Journal Trends

Comment 14: The authors should avoid to give information about methods in the result section : "We matched the journals' titles and ISSNs ..." and subsequent sentences would be better in the Methods section.

Response 14: We agree with the reviewer, and these sentences are now located more appropriately in the Analyses section.

Comment 15: Y-axis of Figure 8 should be labelled more precisely (number of pages?)

Response 15: We appreciate the reviewer's close attention to detail. We have updated the figure's Y-axis to be "Papers' Average Number of Pages."

References

[1] Herrmannova, Drahomira, and Petr Knoth. "An analysis of the Microsoft academic graph." *D-Lib Magazine* 22.9/10 (2016)

Reviewer 2:

Comment 1: The main question of the study ("how and if Goodhart's Law has changed academic publishing") - can't be answered with the presented data and analyses.

Response 1: In this study, our main hypothesis is that Goodhart's Law has changed academic publishing. While this hypothesis has been offered by other studies [1] and supported by a large survey [2], our main goal was to perform a wide-scale big data analysis to examine the effect of the Goodhart's law on academia. Indeed, the bottom-up results presented throughout the study strongly support the impact of Goodhart's law on the academic ecosystem, from the average length of papers' titles to the maximal number of self-citations.

Hypercompetition among researchers can also cause increased productivity and collaboration. Yet, according to Fanelli and Larivière [3], researchers' individual productivity didn't increase in the last century. Nevertheless, we agree with the reviewer that other currently unknown factors may contribute to changing the academic publishing world. However, current evidence of previous studies and our research results strongly support the position that hypercompetition causes measures to be targets; hence, Goodhart's Law is in action.

In the revised manuscript, we discuss additional causes that may be at work to influence academic publishing throughout the last century. Additionally, we have updated the Introduction, Background, Discussion, and Conclusion sections to include these considerations.

References

[1] Edwards, M. A., & Roy, S. (2017). Academic research in the 21st century: Maintaining scientific integrity in a climate of perverse incentives and hypercompetition. *Environmental Engineering Science*, *34*(1), 51-61.

[2] Fong, Eric A., and Allen W. Wilhite. "Authorship and citation manipulation in academic research." *PloS one* 12.12 (2017): e0187394

[3] Fanelli, Daniele, and Vincent Larivière. "Researchers' individual publication rate has not increased in a century." *PLoS One* 11.3 (2016): e0149504

Comment 2: The main conclusion of the study ("While the research environment has changed, the measures to determine the impact of papers, authors, and journals have not changed.") is only correct if the current state of research in the field of bibliometrics is completely ignored.

Response 2: We thank the reviewer for his viewpoint and for providing the useful references. There is no doubt that there are many new, more precise measures that quantify the impact of papers, journals, and authors. Furthermore, many researchers agree that the traditional measures, such as h-index and impact factor, are irrelevant. Moreover, our study supports the usage of these new measures.

However, the traditional measures are still widely used in academia. For example, from our own recent conversations with colleagues from different countries, we were told that committees that evaluate tenure track promotions consider mainly h-index, citation number, and number of Q1 papers. Moreover, many journals publish the journal's impact factor in the landing page of the journal. Furthermore, the most popular academic search platforms, such as Google Scholar, Microsoft Academic, ResearchGate, and Semantic Scholar, present, for each researcher's profile, a variety of traditional metrics, such as number of citations, h-index, and number of papers. Moreover, journals are commonly ranked by these same traditional metrics that are easy to understand. For example, Thomson Reuters Scientific Citation Reports rely on the impact factor to rank journals. Additionally, Google Scholar ranks conferences and journals based on their h-index.

We can make an analogy by considering QWERTY layout still the standard, even though other layouts are more efficient and intuitive (like the Dvorak layout). Many people still use the QWERTY layout because of unawareness, availability, or the ease of consumption. We believe that academia is similar: while researchers have developed new and better bibliometrics, the traditional metrics are still are very widely used, causing a negative effect on the academia ecosystem. For example, currently it is much easier to rank researchers using their number of publications, h-index, and citation number than to evaluate their sum of factorized contributions to each paper, or to calculate normalized metrics based on a publication domain and year of publication.

In the revised manuscript, we elaborate on alternative measures and their advantages (see the Background section). We also update the Discussion section accordingly. On a personal note, we truly hope that this paper will increase awareness of the problems of using the traditional measures, and will promote movement toward using new and better measures.
