# Supplementary material for: Over-optimization of academic publishing metrics: observing Goodhart’s Law in action
Source: Gigascience. 2019 May 30;8(6):giz053. doi: 10.1093/gigascience/giz053 (PMC6541803; doi:10.1093/gigascience/giz053)
Supplement: giz053_Supplement_Figures [file giz053_supplement_figures.pdf]

Supplementary Materials – Additional Results

Additional Results

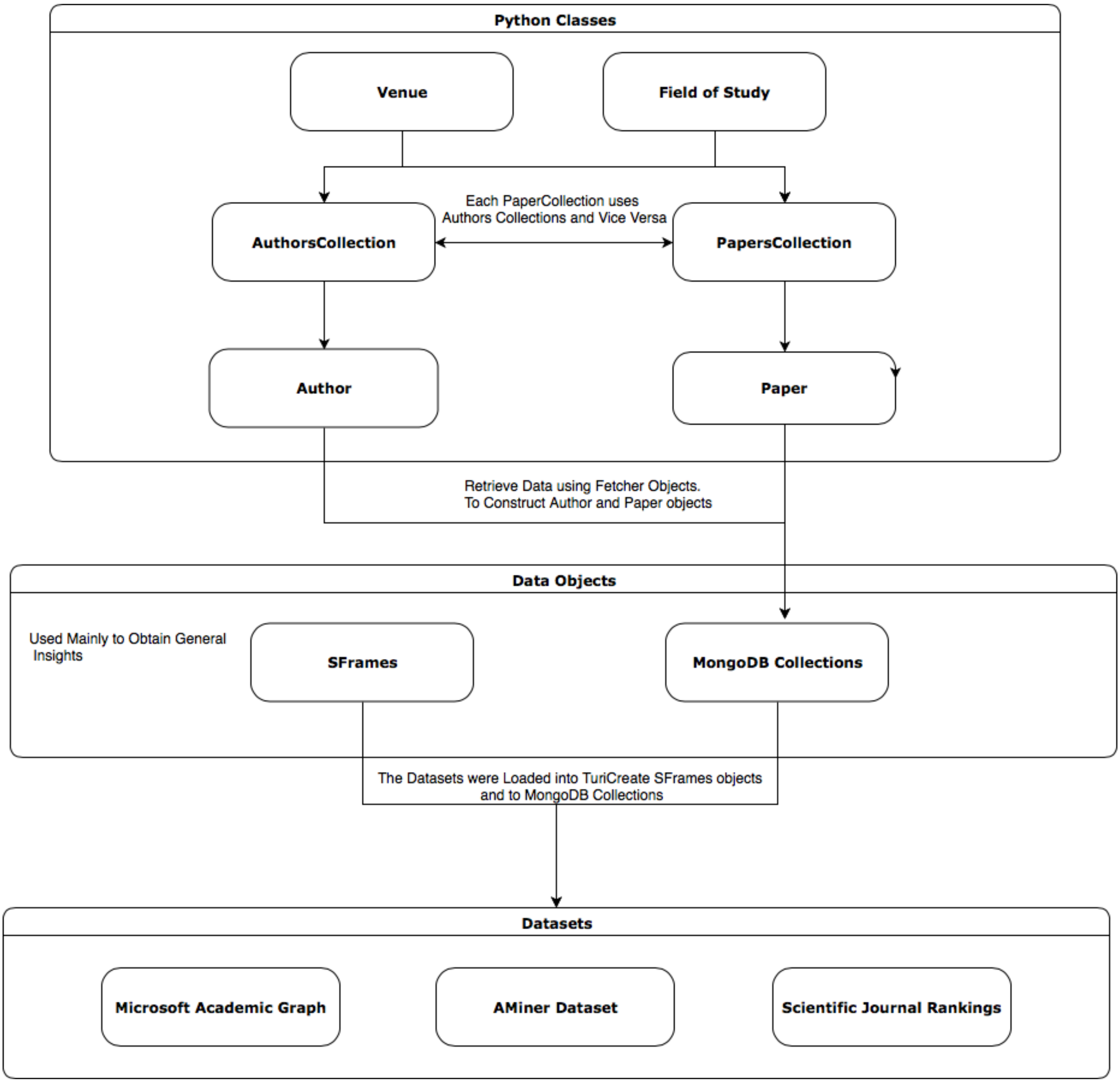

Figure S1. Overview of the Code Framework. The datasets are loaded into SFrame objects and MongoDB collections. The SFrame objects are used mainly to obtain general insights by analyzing tens of millions of papers and author records. The MongoDB collections are used to construct Paper and Author objects that can be used to analyze more complicated statistics for specific venues and research fields with usually hundreds of thousands of records.

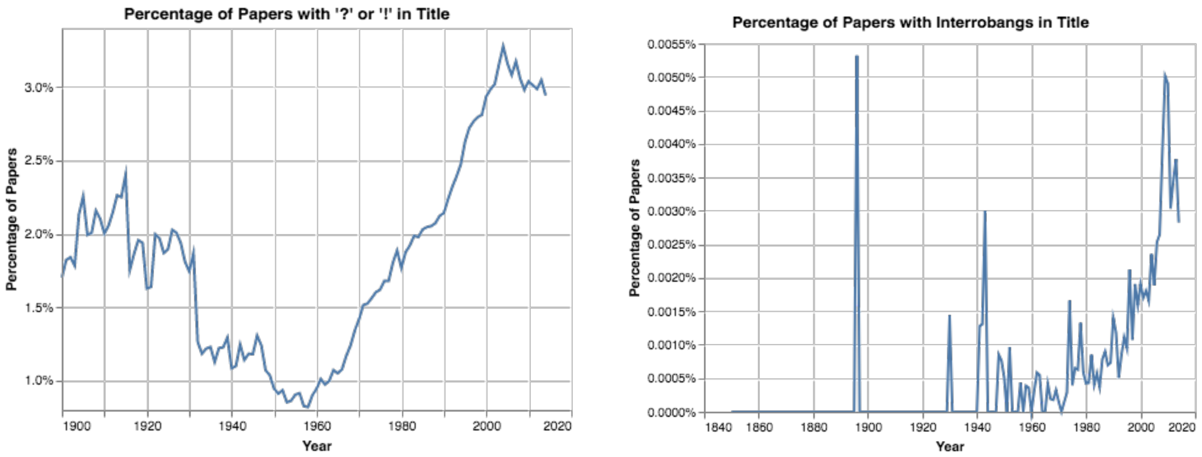

Figure S2. Percentage of Titles with Question or Exclamation Marks. The percentage of papers with question or exclamation marks in their titles increased over time, as well as the percentage of titles with interrobangs (represented by ?! or !?).

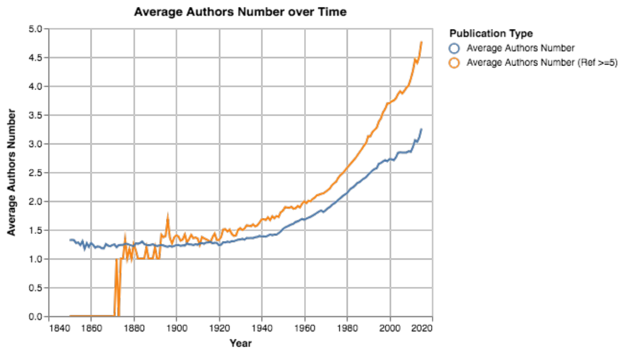

Figure S3. Average Number of Authors over Time. There has been a rise in the average number of authors, especially in recent decades.

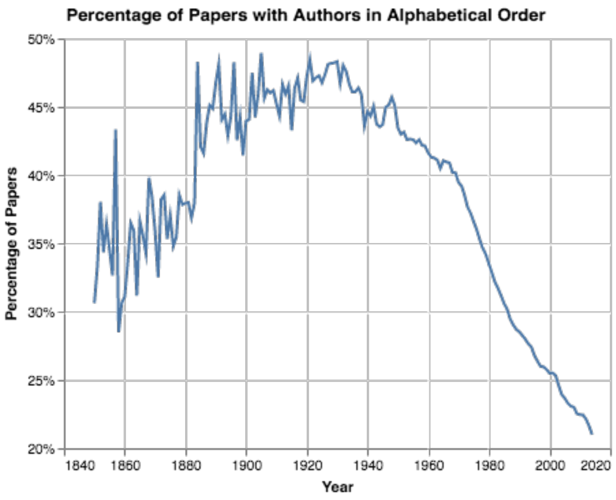

Figure S5. Percentage of Author Lists in Alphabetical Order. There has been a decline in the number of author lists organized in alphabetical order.

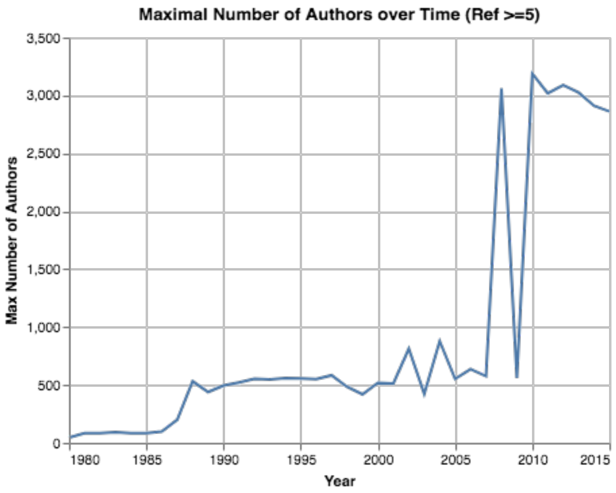

Figure S4. Maximal Number of Authors over Time. In recent years the maximal number of authors per paper increased sharply from 520 authors in 2000 to over 3100 authors in 2010.

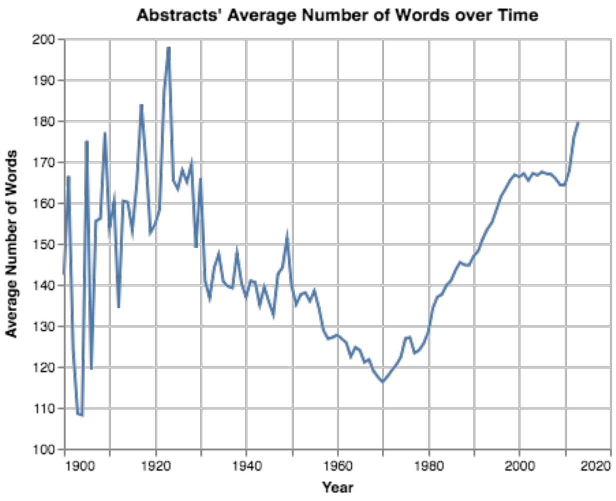

Figure S6. Average Length of Abstracts. Since 1970 there has been an increase in abstracts' average number of words.

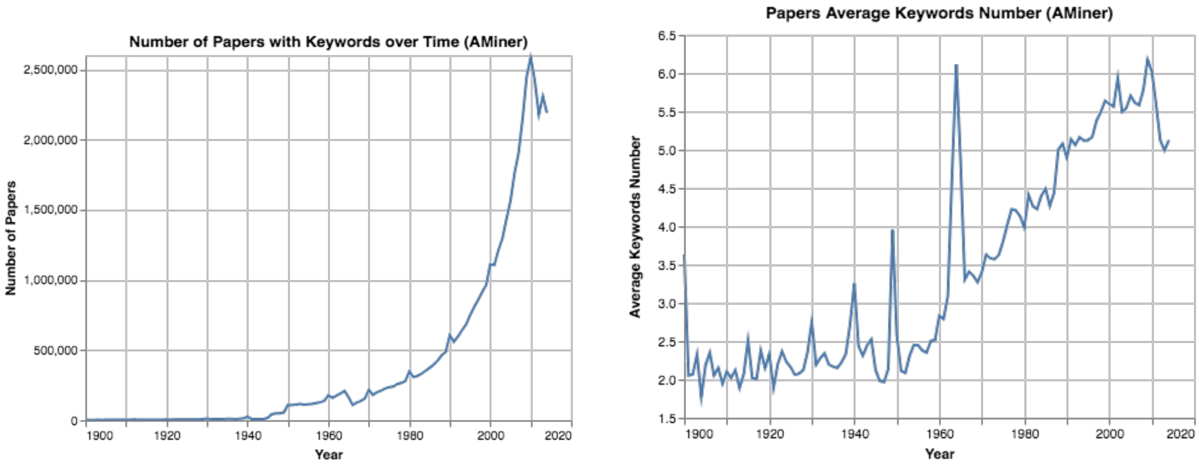

Figure S7. Keyword Trends. Both the number of papers with keywords has increased, as well as the average number of keywords per paper.

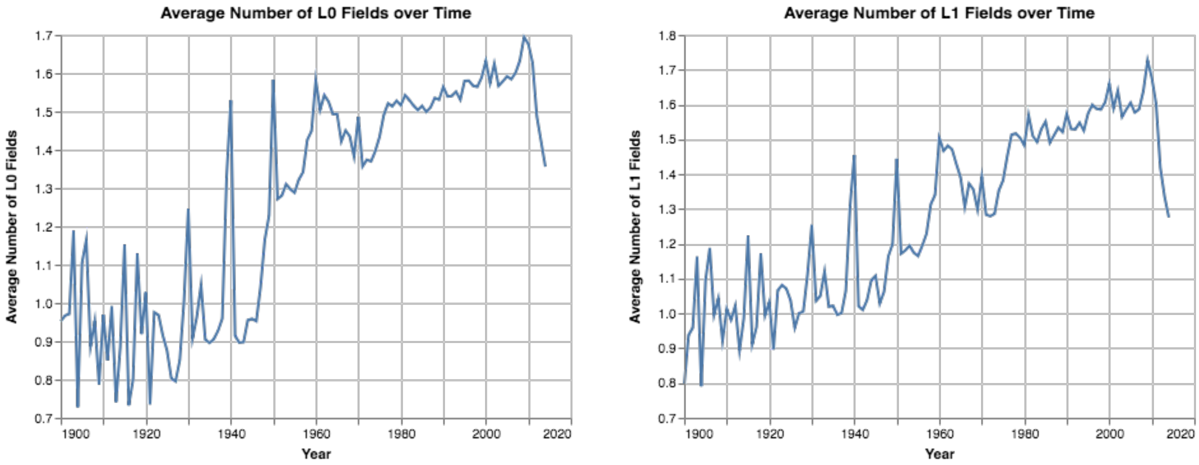

Figure S8. Average Number of Fields of Study over Time. Over time both the average number of L0 and L1 fields of studies per paper considerably increased. We believe the drop in the average number of L0 and L1 fields is a direct results of the drop in the number of papers with keywords in the same years (see the Results of Paper Trends) section.

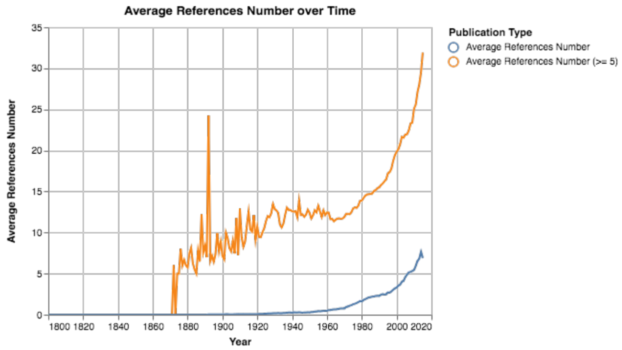

Figure S9. Average Number of References over Time. Over time, the average number of references sharply increased.

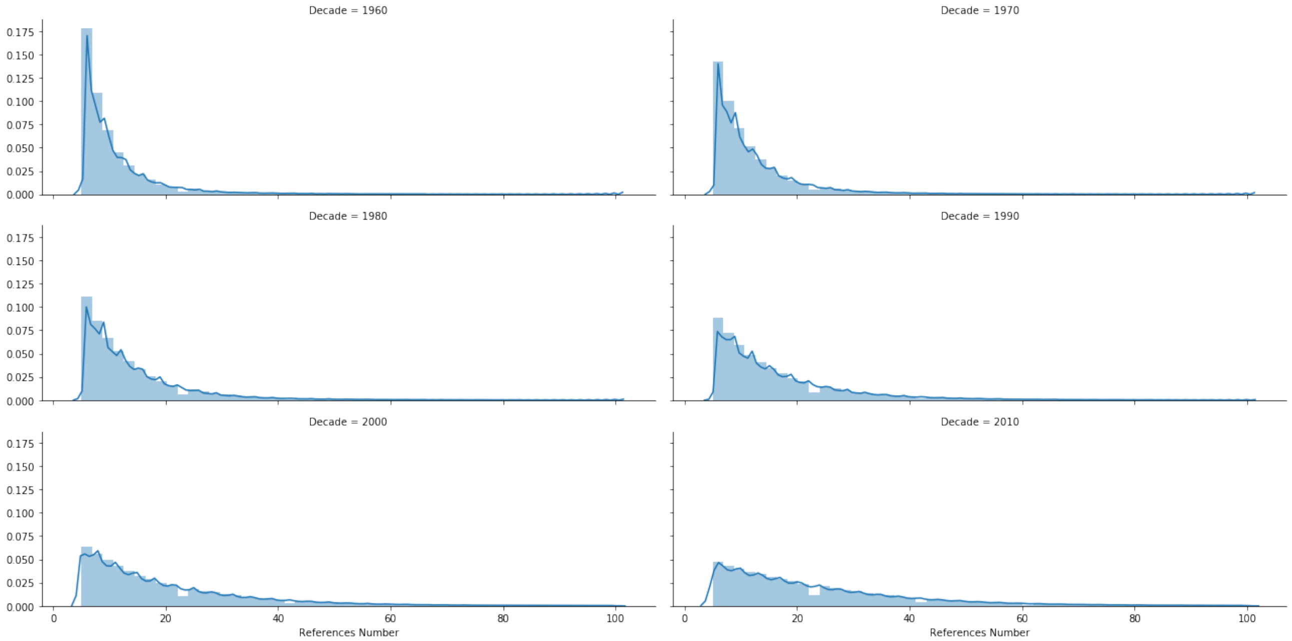

Figure S10. Distributions over Time of References in Papers. Over time, papers with a relatively high number of references have become more common.

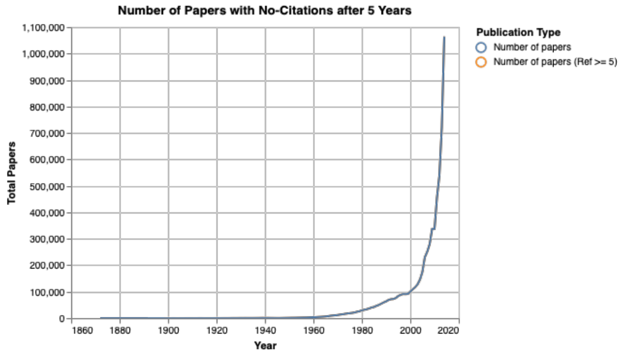

Figure S11. Total Number of Papers with No Citations after 5 Years. The number of papers with increased sharply over time.

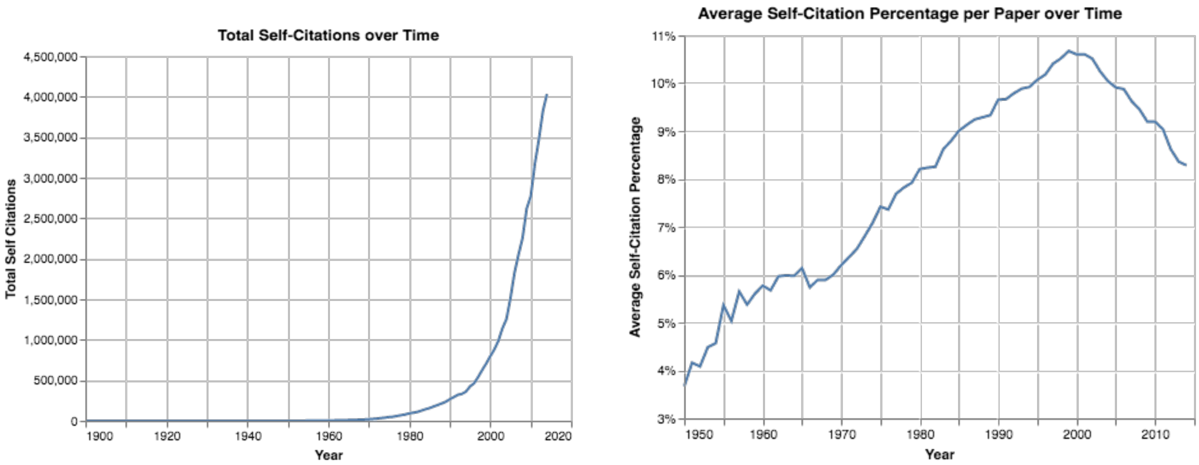

Figure S12. Total Number of Self-Citations and Percentage of Papers with Self-Citations. We can observe that over time both the total number of self-citations as well as the percentage of papers with self-citations increased significantly.

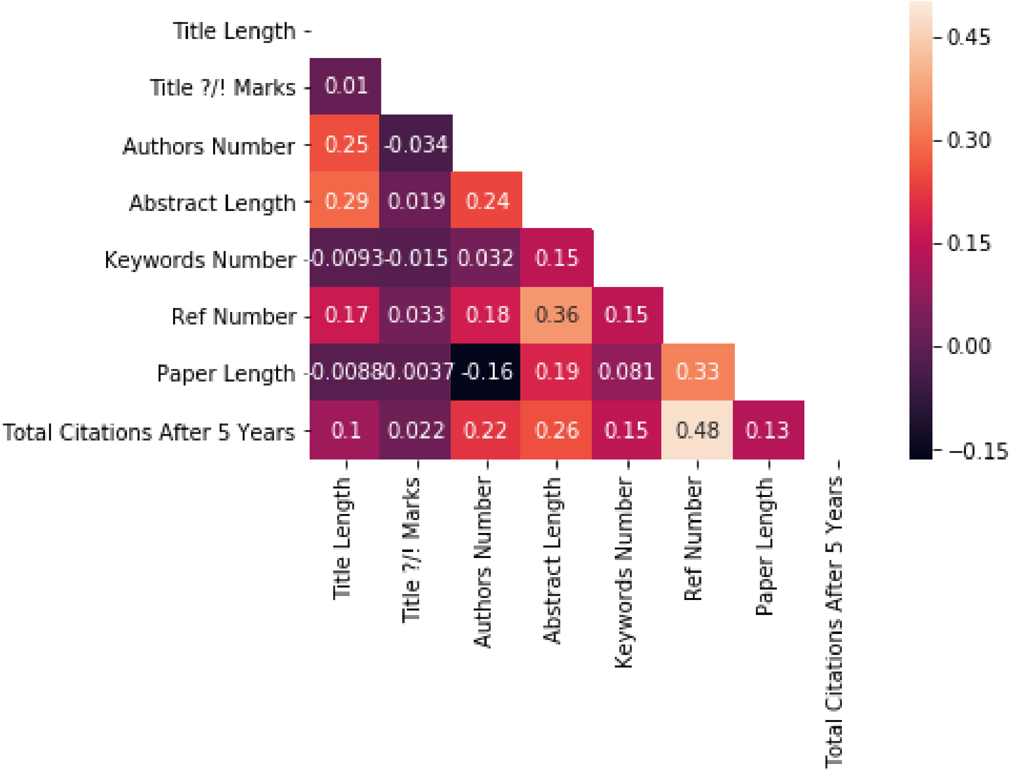

Figure S13. Spearman Correlation Heat Map for Papers' Properties. We can observe positive correlations among papers' various structural properties and the papers' total number of citations after 5 years.

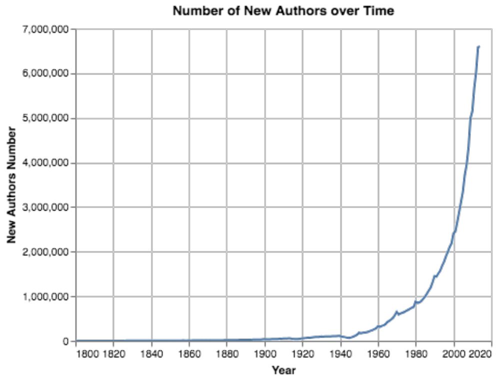

Figure S14. New Authors over Time. The number of authors, with unique MAG author IDs, who published their first paper each year.

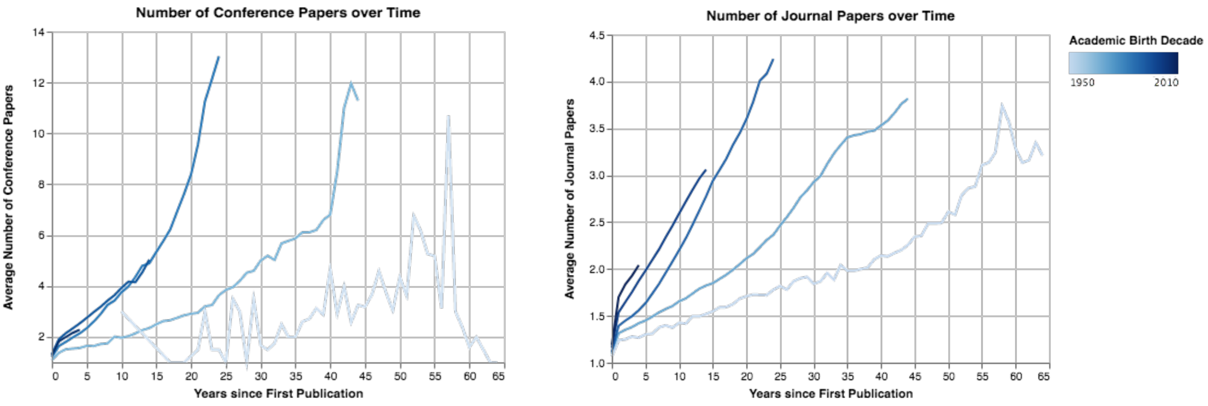

Figure S15. Authors Average Number of Conference and Journal Papers over Time. The average publication rate of both journal and conference papers increased with every decade.

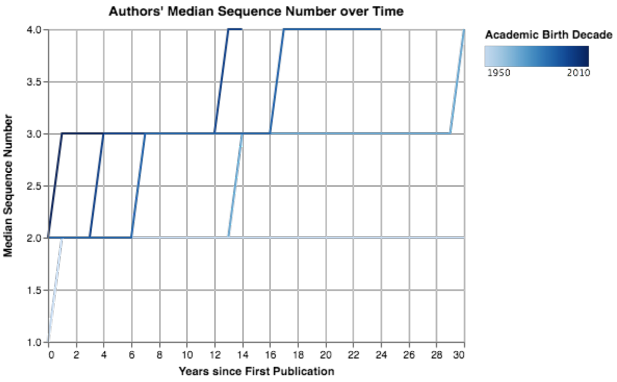

Figure S16. Authors' Median Sequence Number over Time. We can see that over time the median sequence numbers increased; i.e., senior researchers tend to have higher sequence numbers.

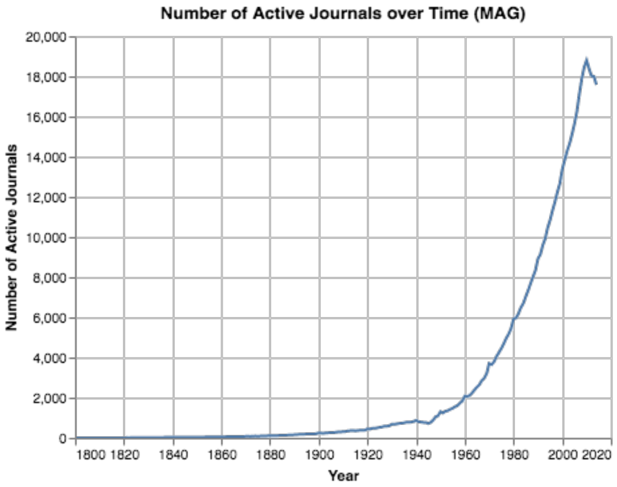

Figure S17. Number of Journals over Time according to the MAG Dataset. There has been a drastic increase in the number of journals since the 1960s.

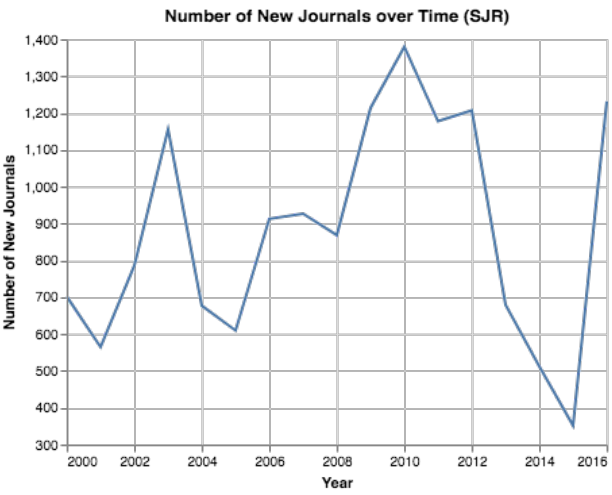

Figure S18. Number of New Journals by Year. Hundreds of new ranked journals are being published each year.

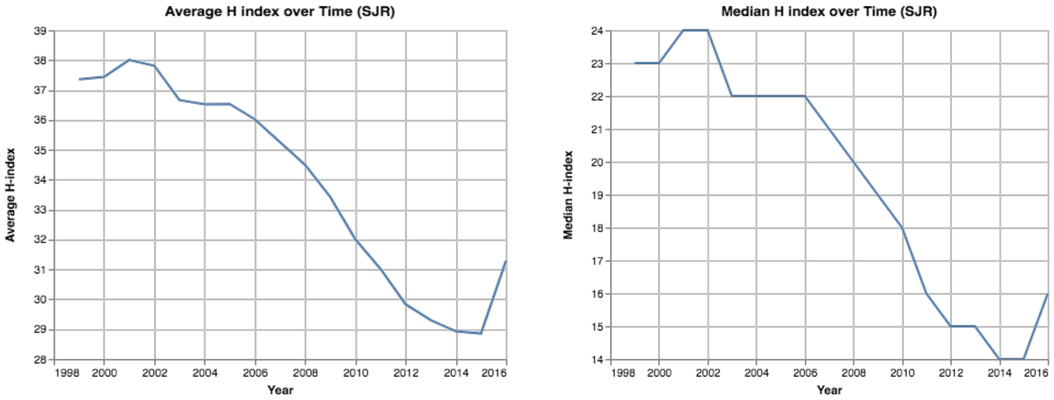

Figure S19. Journals' H-Index Average and Median Values. We can notice that over time both the average and median values of the journals' h-index measures decreased.

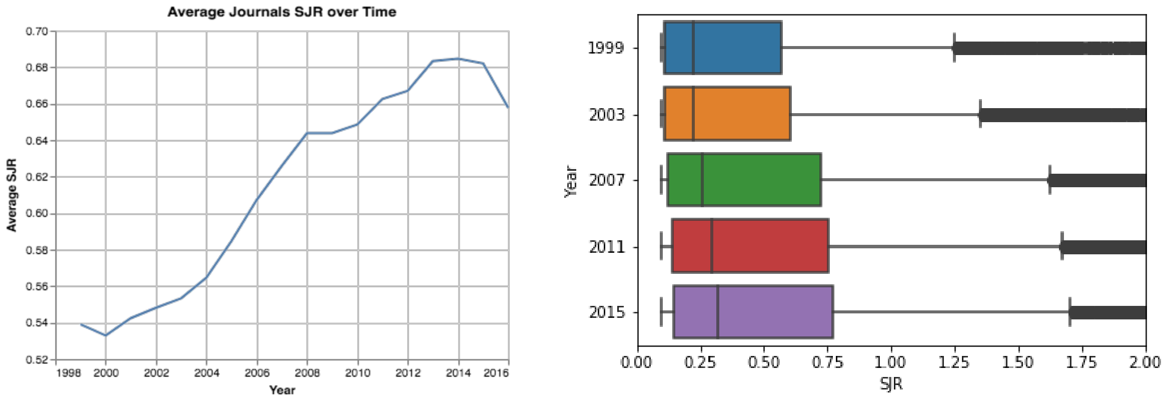

Figure S20. SJR Values over Time. We can observe that over time both the average and median SJR values increased.

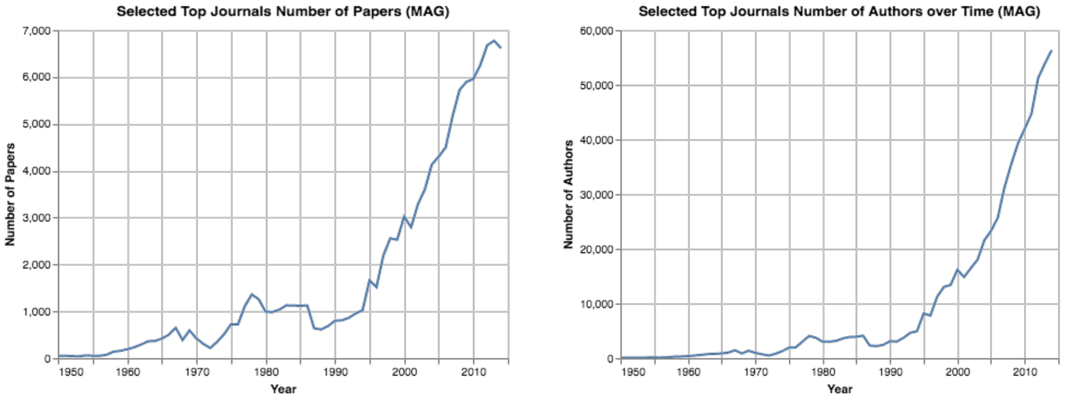

Figure S21. Top Journals' Number of Papers and Authors over Time. We can observe that both the number of papers and authors increased sharply in recent years.

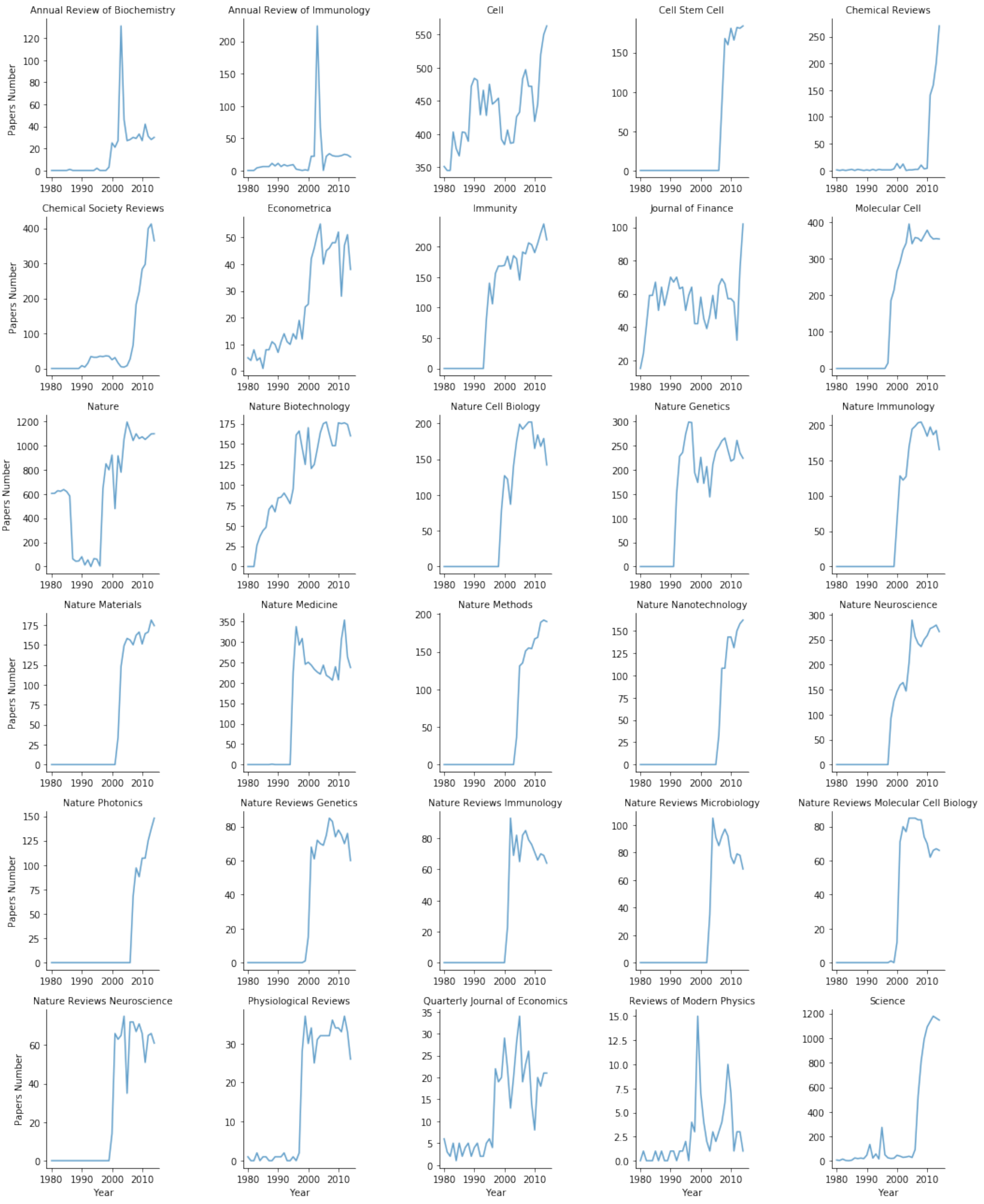

Figure S22. Top Selected Journals' Number of Papers over Time. It can be noted that in the vast majority of the selected journals the number of published papers with at least 5 references increased considerably over time.

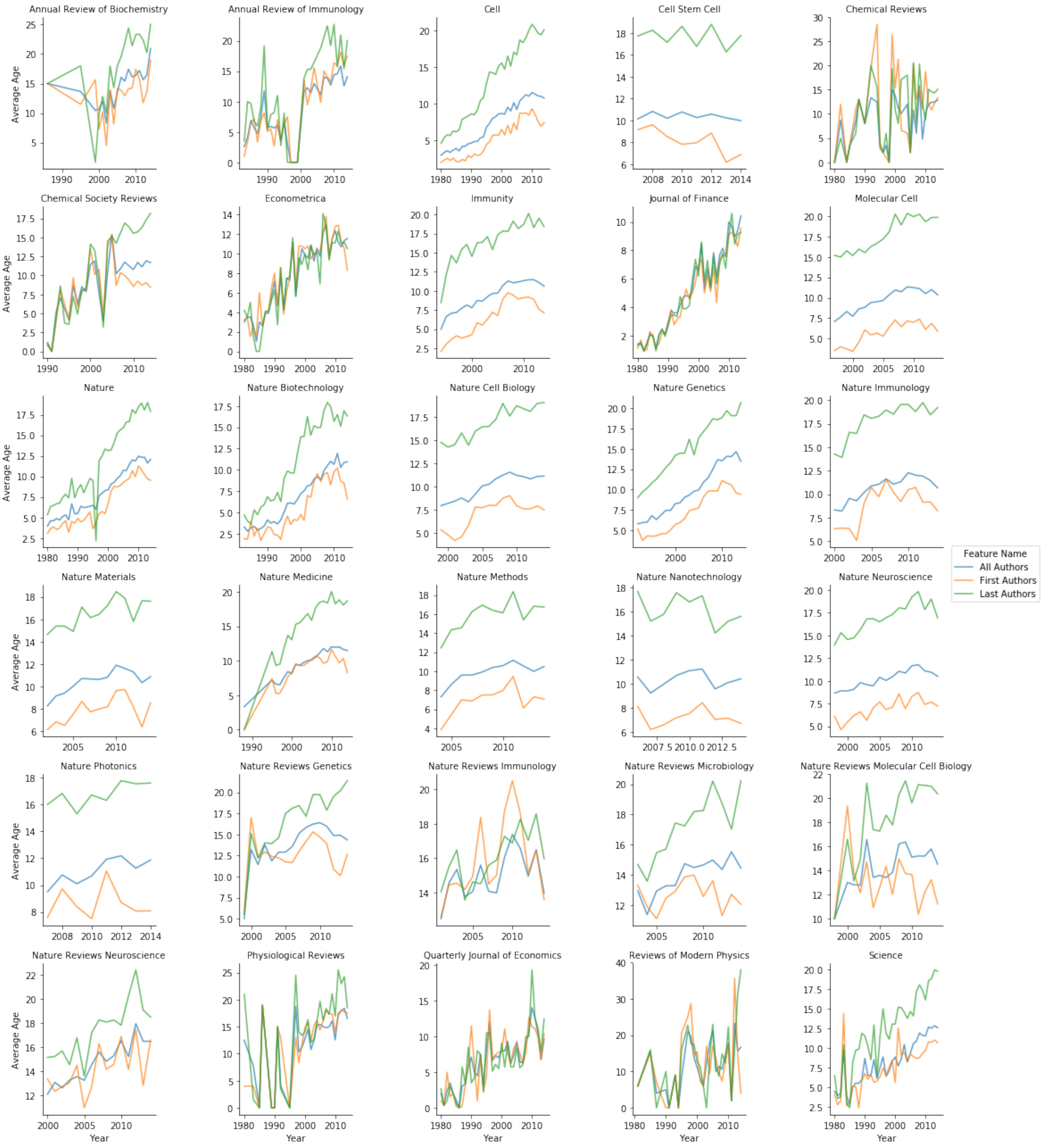

Figure S23. Top Selected Journals Average Author Career Age over Time. It can be noted that in the vast majority of the selected journals, the average age of authors, especially last authors, increased greatly over time.

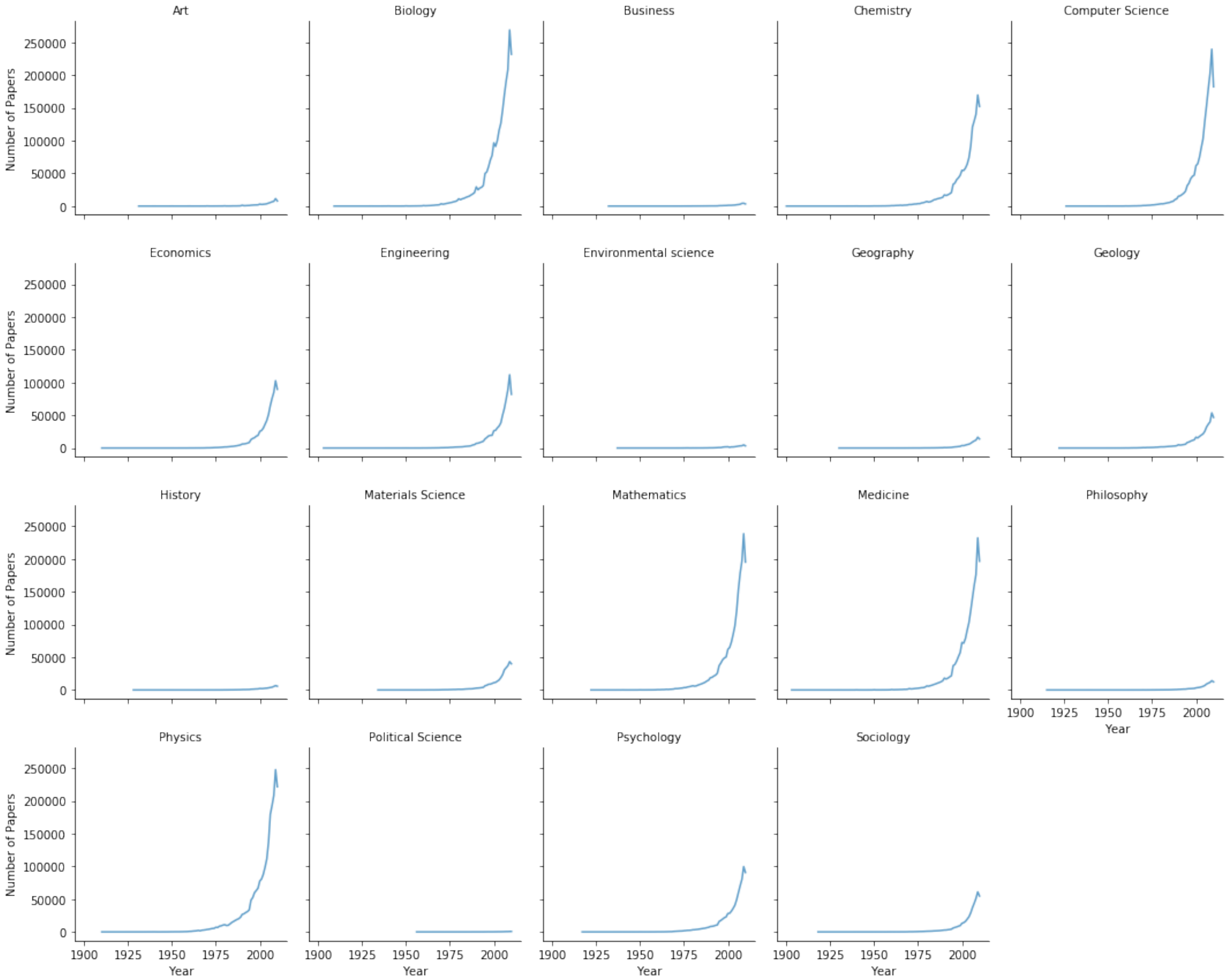

Figure S24. L0 Fields-of-Study Number of Papers over Time. We can observe the large diversity in the number of papers published in each L0 research field.

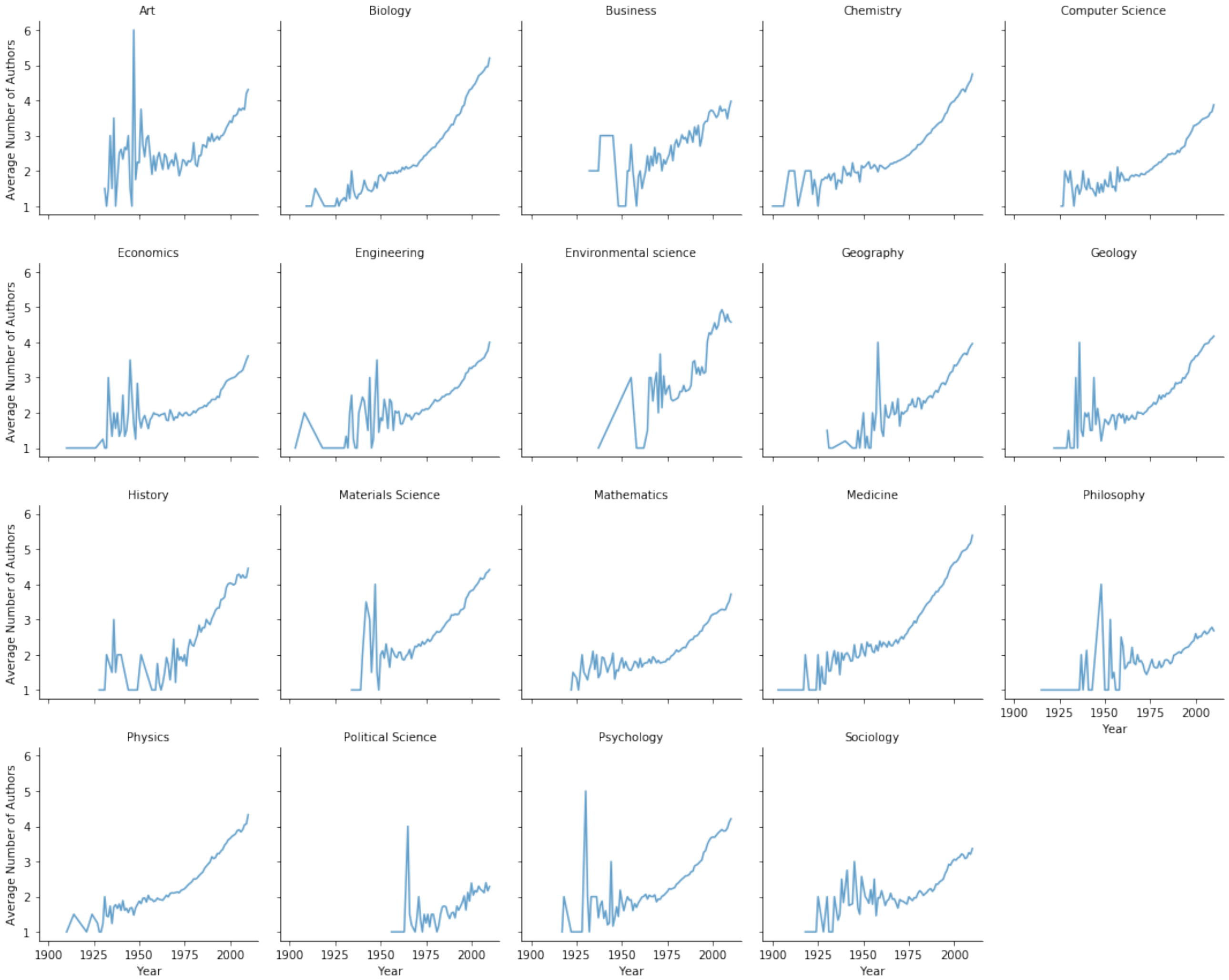

Figure S25. L0 Fields-of-Study Average Authors Number. We can observe a variation in the average number of authors across the various research fields.

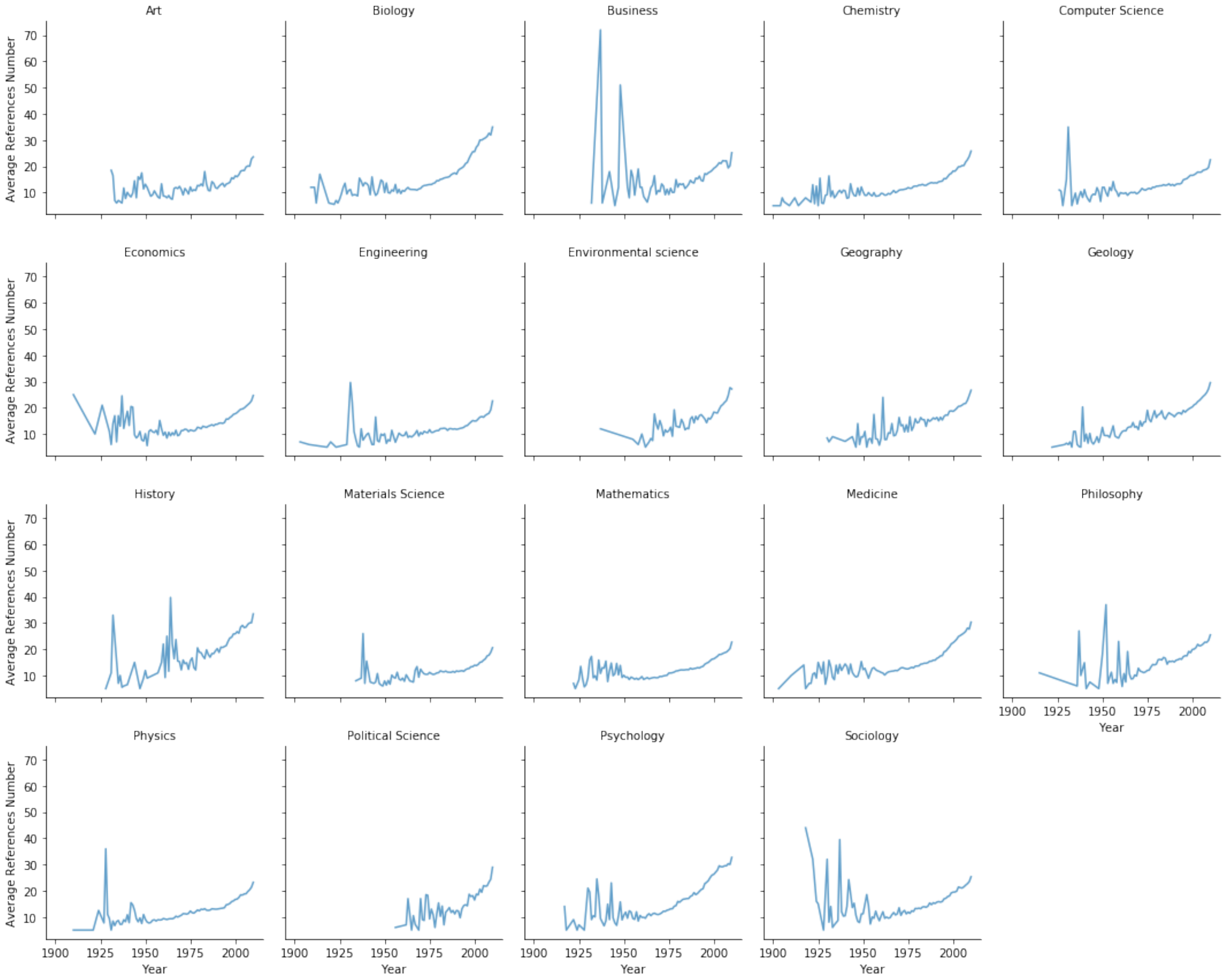

Figure S26. L0 Fields-of-Study Average References Numbers. We can observe variance among the reference numbers in different fields.

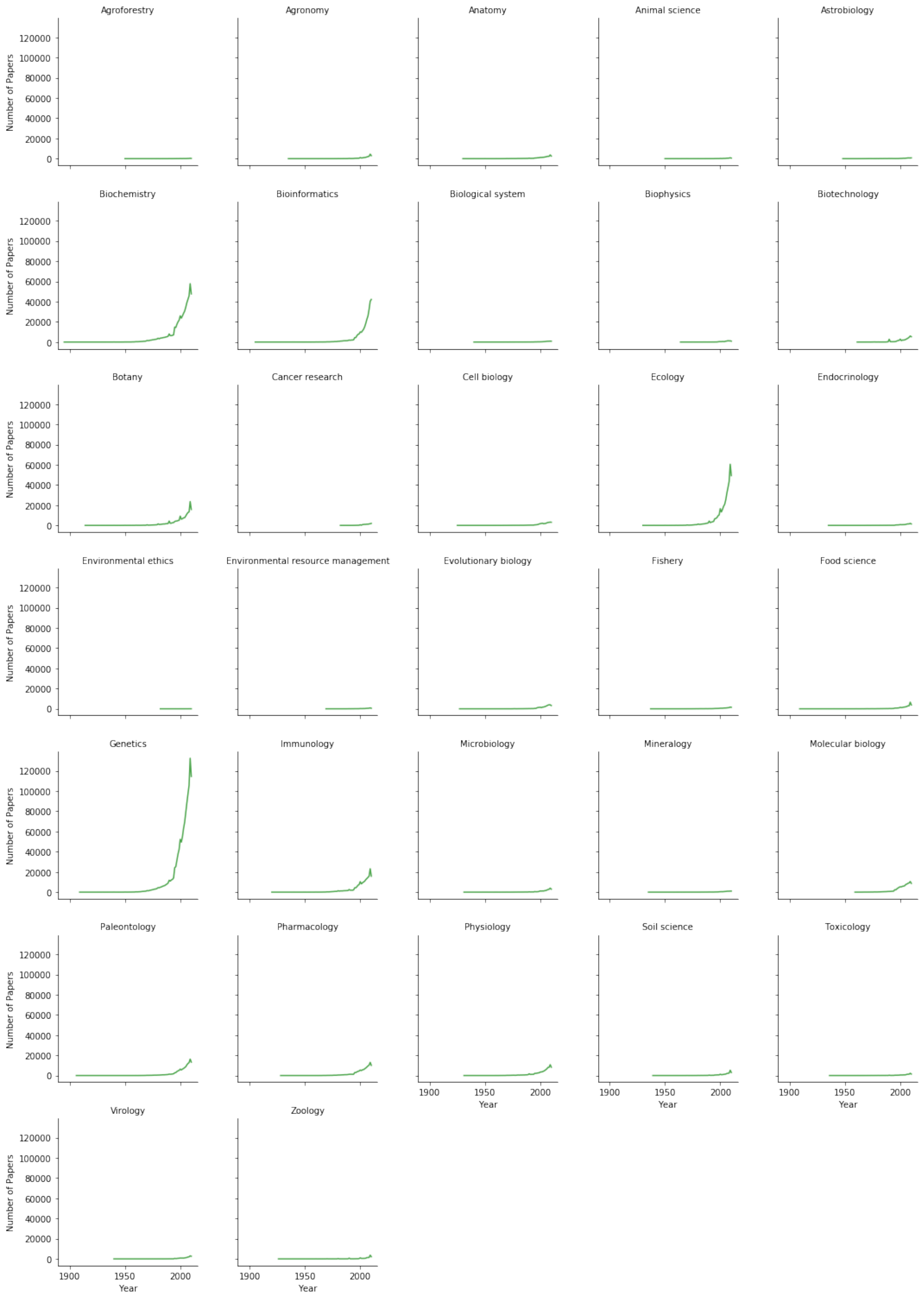

Figure S27. Biology L1-Subfields Number of Papers over Time. We can observe a big variance in the number of papers over time in the various biology subfields.

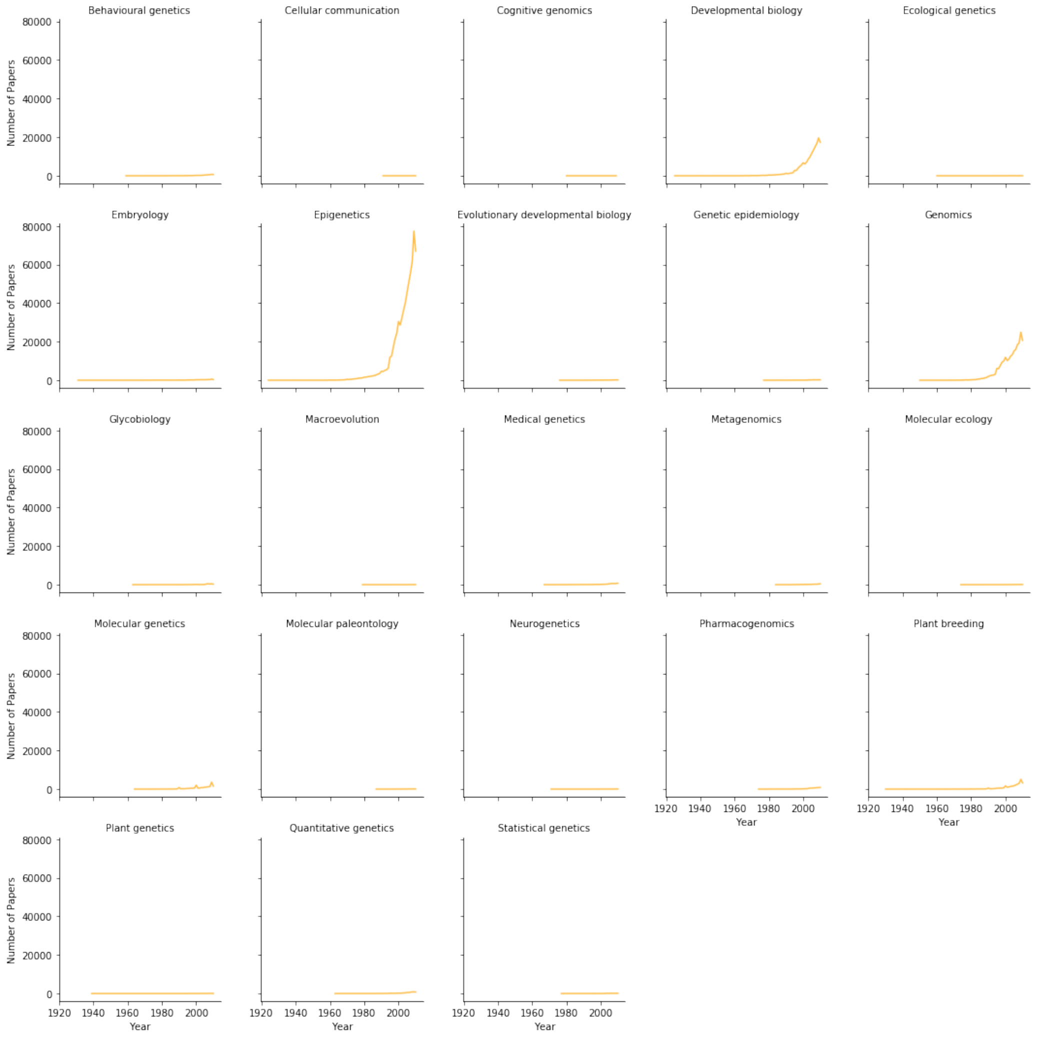

Figure S28. Genetics L2-Subfields Number of Papers over Time. We can observe a big variance in the number of papers over time in the various genetics subfields.

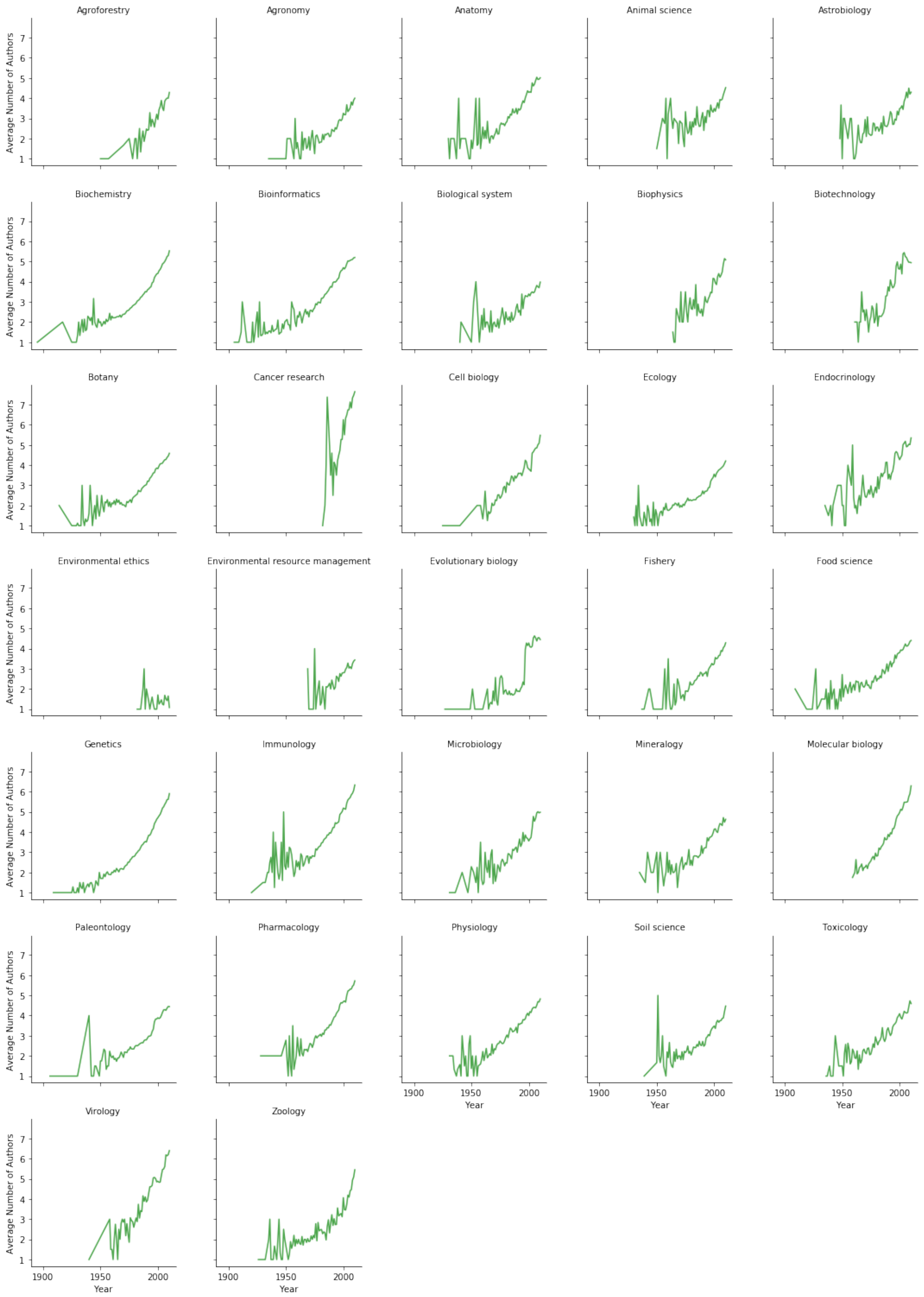

Figure S29. Biology L1-Subfields Average Number of Authors over Time. We can observe a variance in the average number of authors over time in the various biology subfields.

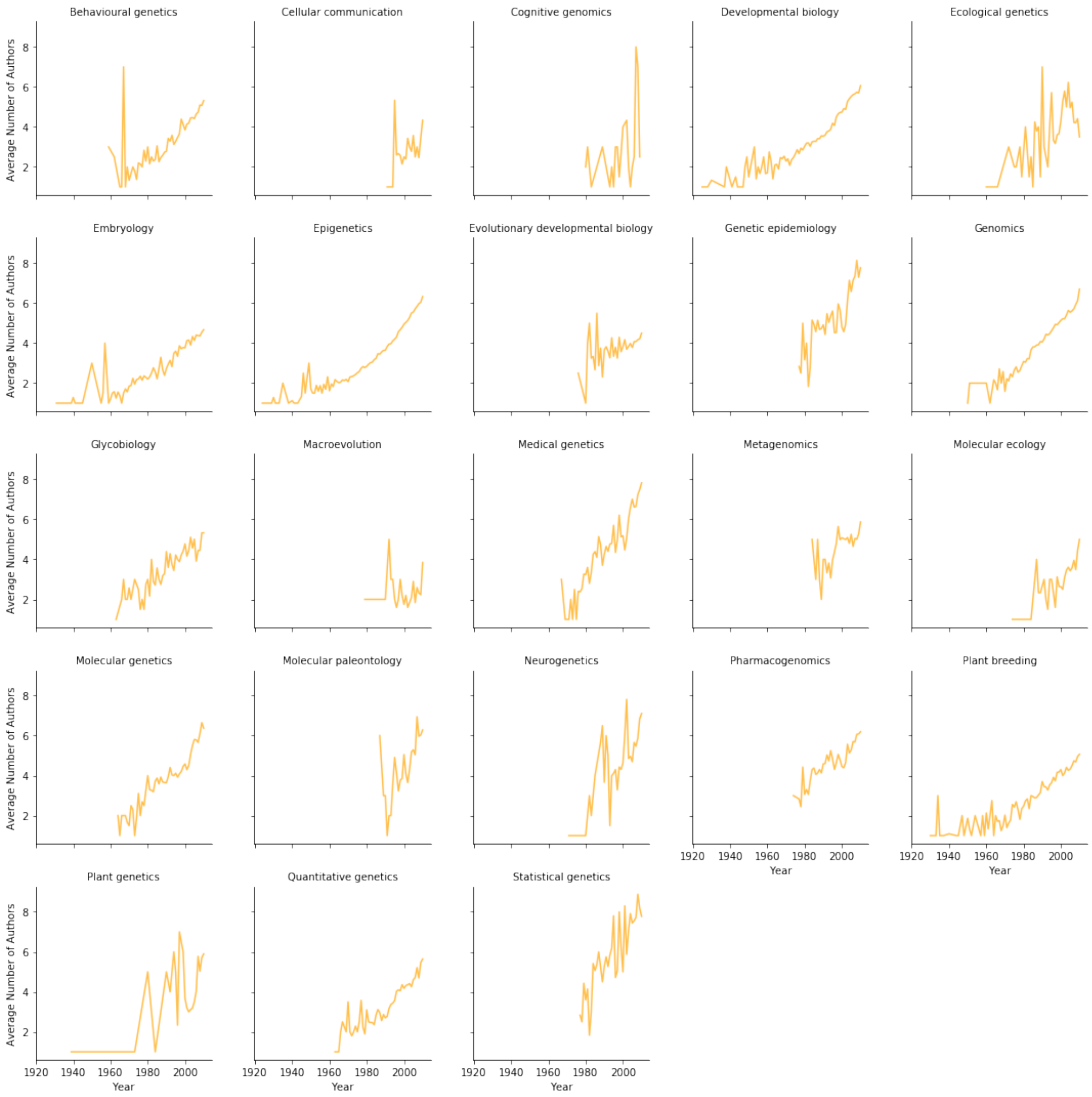

Figure S30. Genetics L3-Subfields Average Number of Authors over Time. We can observe a significant variance in the average number of authors over time in the various genetics subfields.

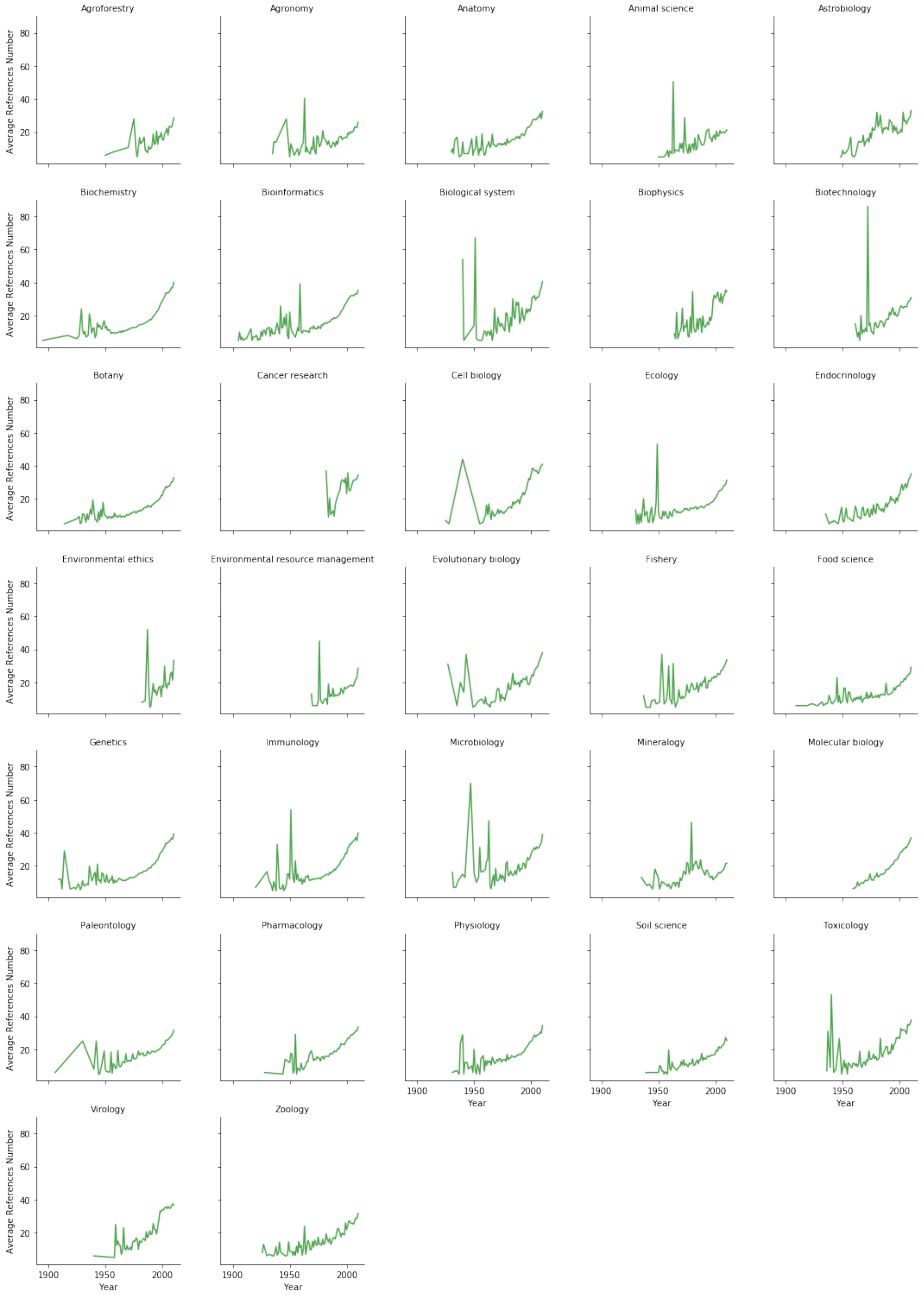

Figure S31. Biology L1-Subfields Average Number of References over Time. We can observe a variance in the average number of references over time in the various biology subfields.

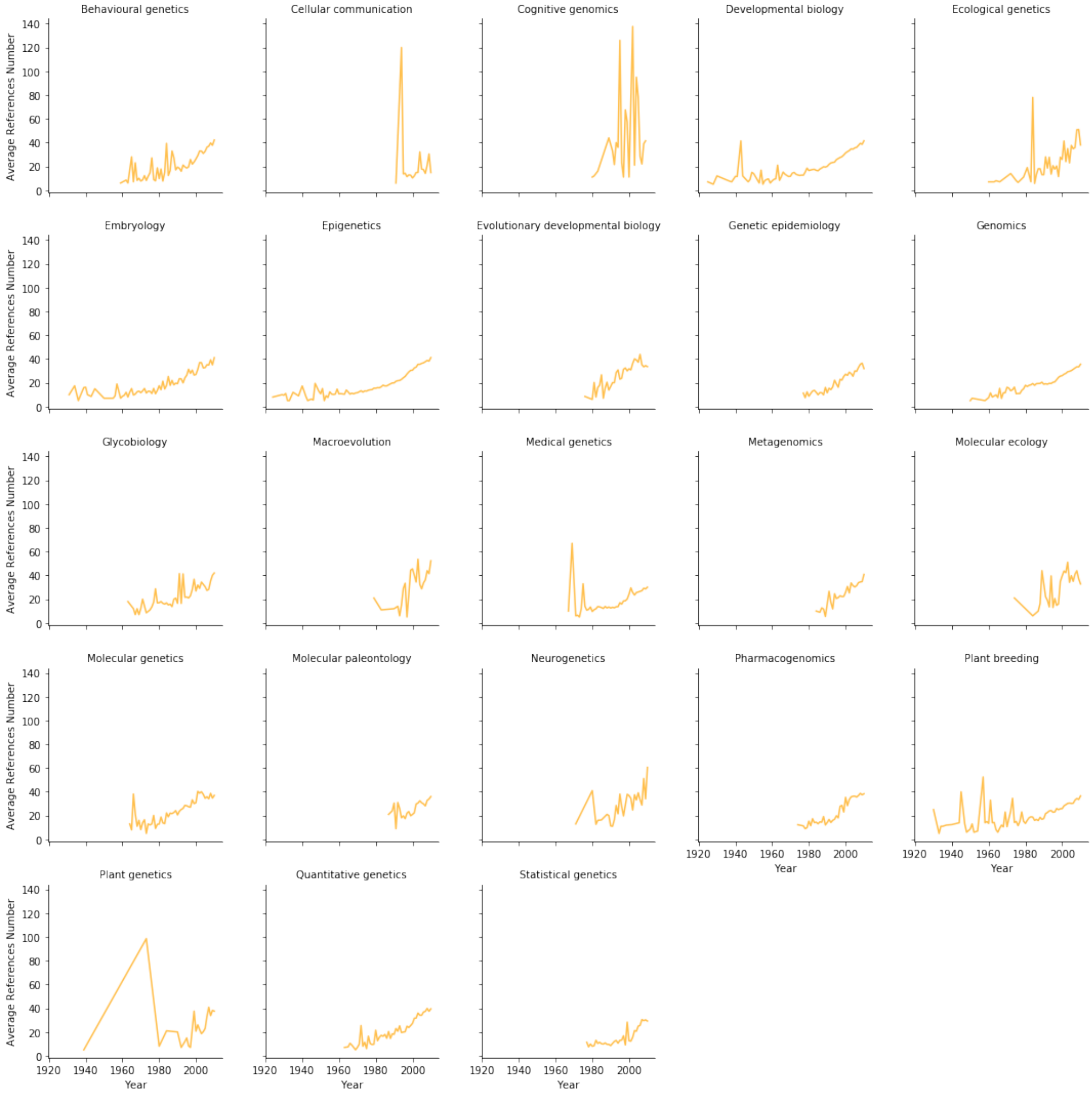

Figure S32. Genetics L2-Subfields Average Number of References over Time. We can observe a significant variance in the average number of references over time in the various genetics subfields.

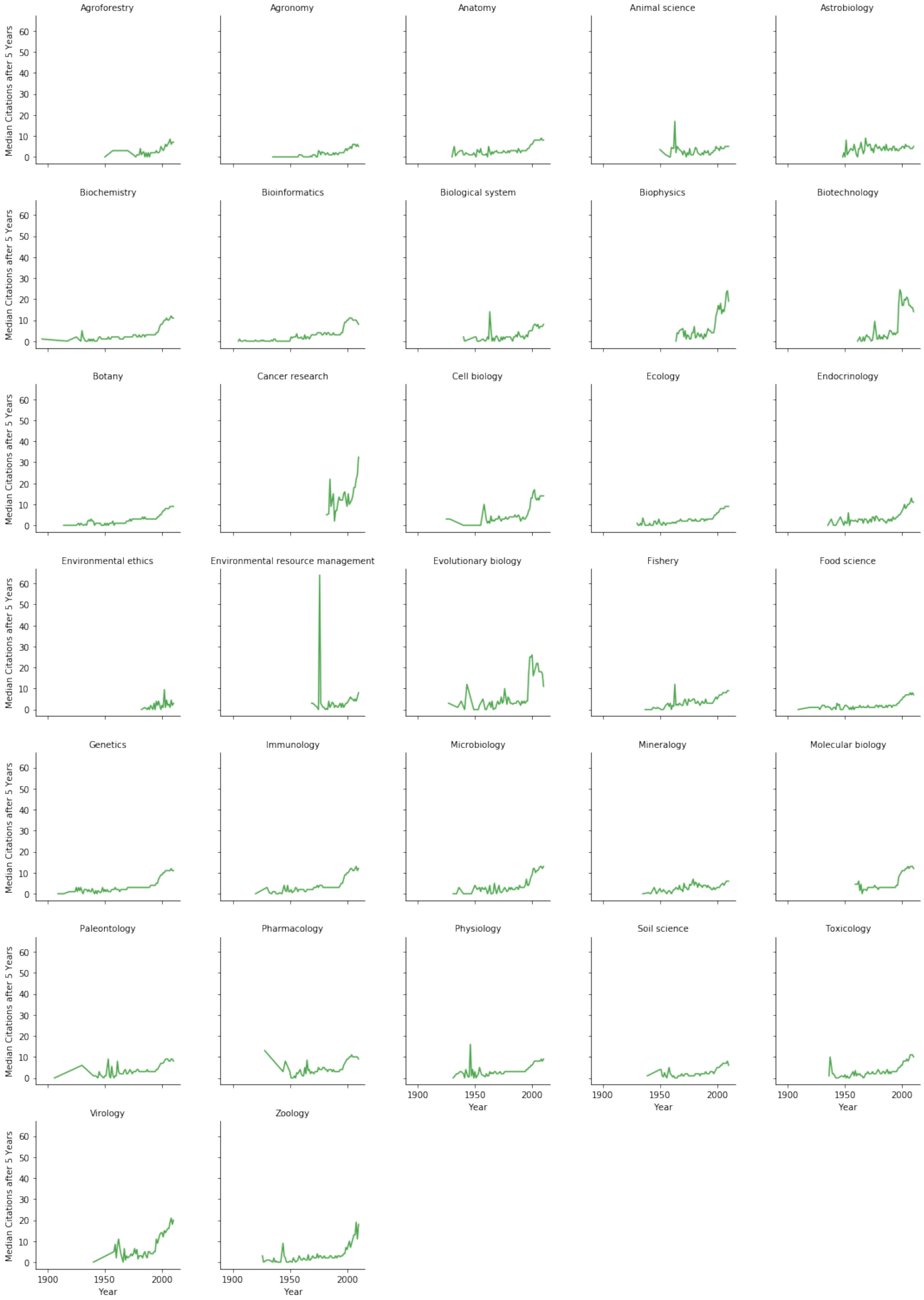

Figure S33. Biology L1-Subfields Median Number of 5-Year Citations over Time. We can observe a variance in the median number of citations over time in the various biology subfields.

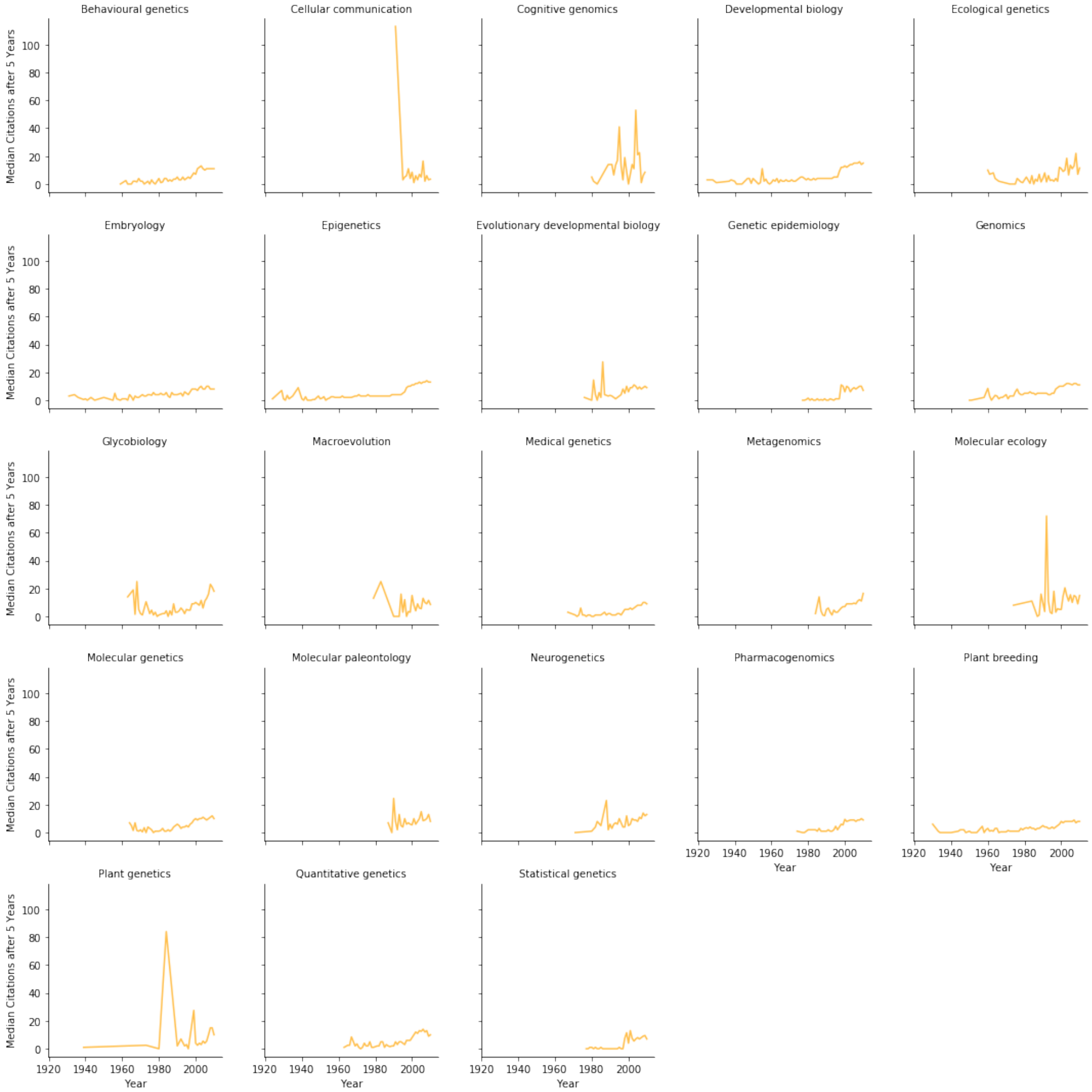

Figure S34. Genetics L2-Subfields Median Number of 5-Year Citations over Time. We can observe a significant variance in the median number of citations over time in the various genetics subfields.

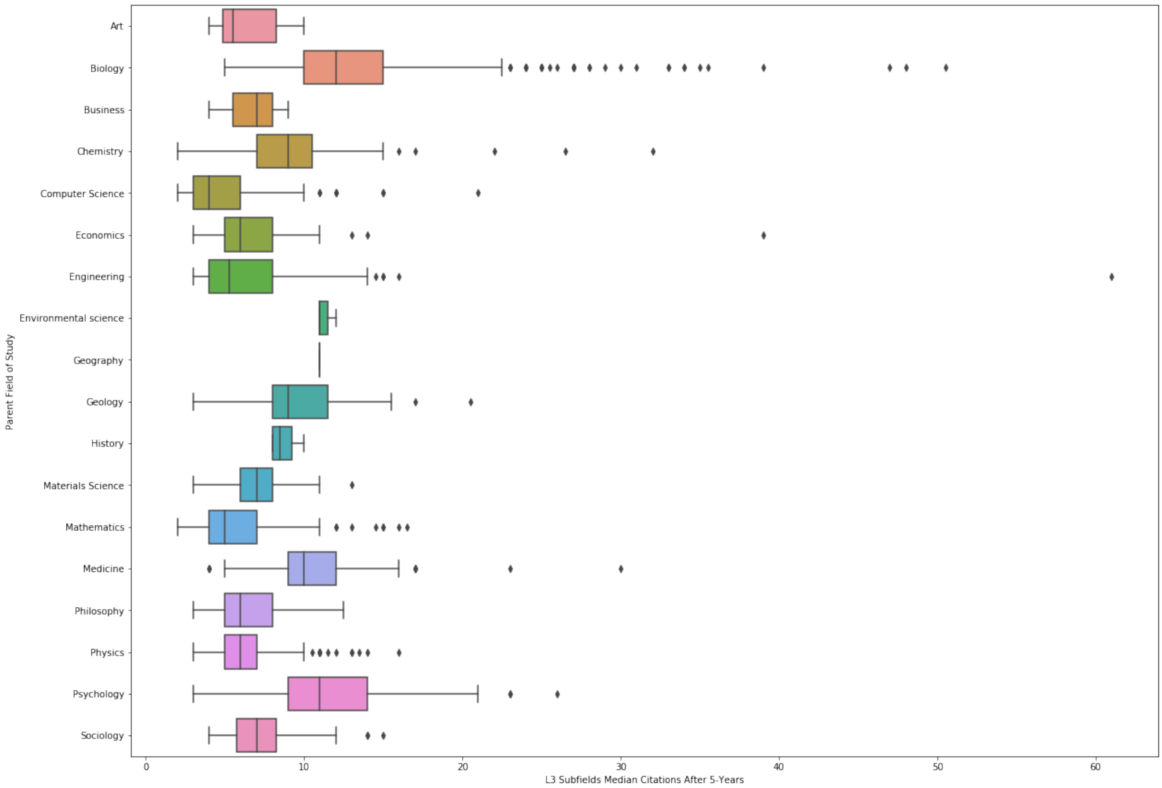

Figure S35. L3 Fields-of-Study Median 5-Year Citation Distributions by Parent Fields. We can observe the high variance among the L3 fields-of-study median citation numbers.

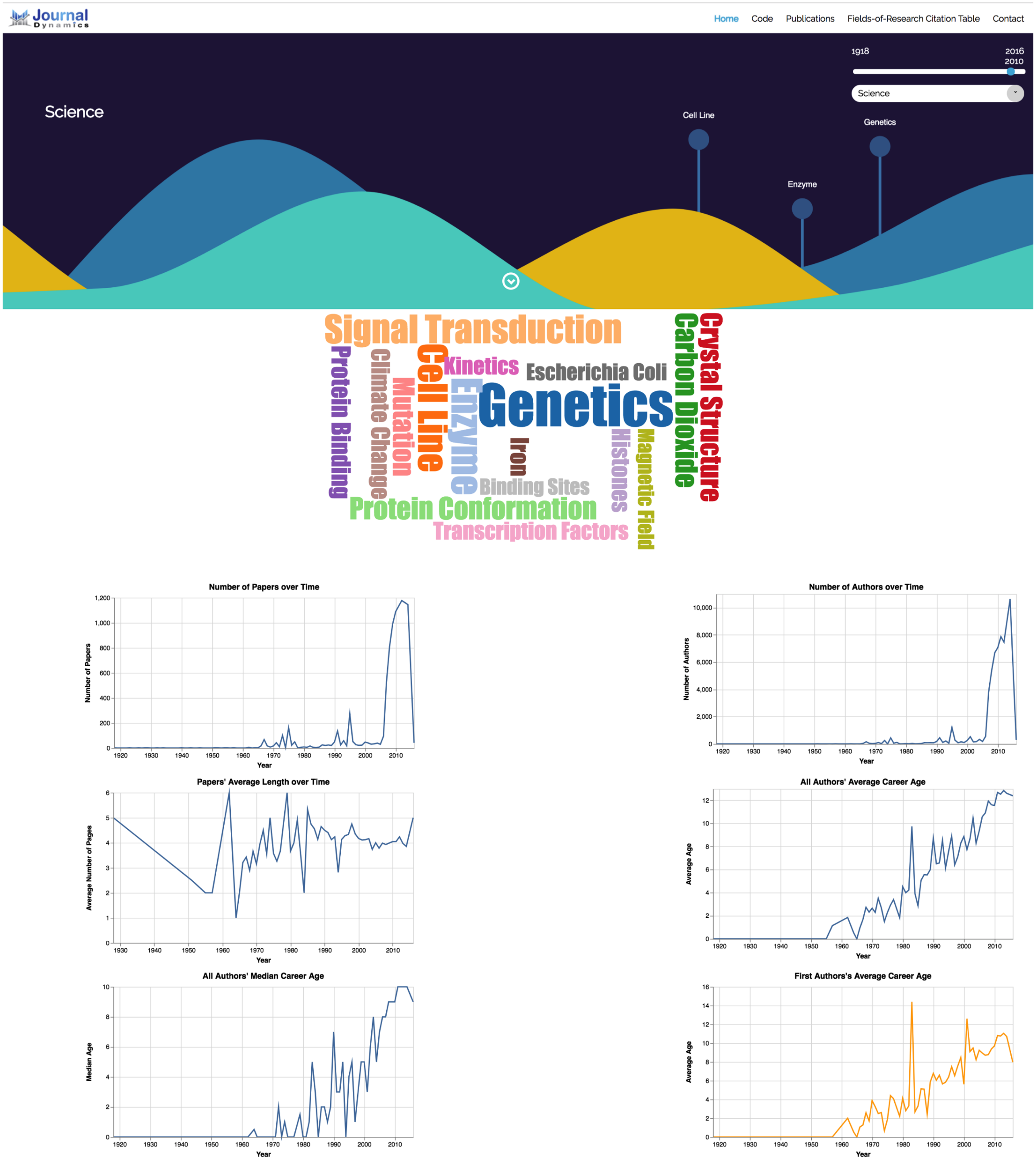

Figure S36. Interactive Website. We have developed an interactive website that makes it possible to view and interact directly with the study's data.
