# Supplementary material for: Over-optimization of academic publishing metrics: observing Goodhart’s Law in action
Source: Gigascience. 2019 May 30;8(6):giz053. doi: 10.1093/gigascience/giz053 (PMC6541803; doi:10.1093/gigascience/giz053)
Supplement: giz053_Response_to_Reviewer_Comments_Original_Submission [file giz053_response_to_reviewer_comments_original_submission.pdf]

Author's Response To Reviewer Comments

Close

--- Please note that the response letter to reviewers' comments is also attached as a PDF file ---

"Over-Optimization of Academic Publishing Metrics: Observing Goodhart's Law in Action" Response to Reviewers

We would like to thank the reviewers for their highly valuable and constructive criticism. The comments have been very helpful in the preparation of the revised manuscript.
We have addressed the reviewer’s concerns and have improved the article accordingly.
The following is a description of the revisions we have made in order to address the comments pointed out by the reviewer.

Reviewer 1:

- Introduction

Comment 1: Figure 1 is very interesting, however, it appeared far too early in the paper.
It makes the figure not very understandable (readers at this stage have no idea on how these data have been collected and analyzed). Maybe Figure 1 should be provided as a summary-of-results, later in the paper (in the discussion section?)

Response 1: We thank the reviewer for this comment. We have moved Figure 1 (now labeled as Figure 22) to the Discussion section.

Comment 2: A citation (or several citations if needed) from the literature would be sufficient to describe the exponential growth of academic publishing.
Introduction shouldn't reports results from the present study (so Figures 14 and S17 shouldn't be mentioned in this section) -
numbering of figures should also be checked in the entire article (Figure 14 shouldn't follow the Figure 1 in the order of apparition).

Response 2: As suggested, we have added citations to the relevant work for describing the exponential growth of academic publishing.
In addition, we have removed the reference to figures from the current study. Furthermore, we have updated the numbering of the figures so they are ordered correctly.

Comment 3: Again in the Introduction section, paragraphs Papers, Authors, Journals and Fields of Research are in fact summary of results.
It should be reported later in the paper. The introduction section should present hypotheses that were formulated before analyses were performed.
Paragraphs "These observations support the hypothesis []... (see the Results of Paper Trends section and Figure S13)" and
"It is time to consider [...] academic publishing world" are in fact discussion paragraphs.
There is a need in the introduction section to formulate the general objective of the paper.

Response 3: We have revised the Introduction section according to these helpful comments. The paragraphs related to results have been removed, as have those that fit better in
the Discussion section. Moreover, we have inserted a paragraph to make the general objective of the paper very clear.

- Background

Comment 4: The sentence "In this section, we give a short overview of the relevant scientometric papers to this study" is unclear.
Suggestion: "In this section, we present studies that analyze changes in academic publications in recent years ..."

Response 4: In the revised manuscript, we have changed the text according to the above suggestion.

Comment 5: Paragraph " Our study is greatly influenced by a recent study by [...](and hence the status) of the research." would be better in the introduction section.

Response 5: We agree with the reviewer's comment, and we have moved this paragraph to the Introduction section.

- Data Description

Comment 6: DOI is a good way of identifying an article, but the "unique author ID value" is not very clearly explained.

Response 6: One of the interesting challenges in analyzing bibliometric datasets is solving the author disambiguation problem.
In other words, in order to calculate various statistics, it would very helpful to know which papers were written by the same author.
The problem is that in many cases matching an author to a paper can be extremely challenging. For example, researchers can change their last names, affiliations, and even their research domains
. If a researcher published only few papers under one name and then changed his/her name, it is very difficult to match the old papers to the new name.
Moreover, there are some names that are extremely common. To tackle this challenge, the MAG dataset uses an author disambiguation algorithm and sets
a unique author ID value to each author identified by the algorithm. Recently, Microsoft Academic released a post that explains how they address the problem of conflation/disambiguation.

In the revised manuscript, we have elaborated on the unique author ID value and added relevant references.

Comment 7: it is quite uncommon to use these datasets for scientometric purposes (if not, please provide examples of such previous use).
Authors should better explain why they use these datasets instead of more traditional databases (e.g, for biomedical research, scopus, embase, medline, psychinfo etc).
They should also explain how fields of research are integrated into these datasets, how complete they are, how representative of the literature they are.
Main comment of this reviewing: More precisions on datasets that have been used are very important to assess external validity of the present analyses
(are the references included in these datasets representative of the overall knowledge?).:

Response 7: In recent years with the significant advantages of data science tools, the availability of big-scale datasets, and the advancements in cloud computing,
it has finally become possible for researchers to analyze big datasets, such as MAG and AMiner. For example, about six years ago when we needed to analyze a large-scale dataset,
we required a strong Hadoop cluster with dozens and even hundreds of nodes (in one case we used thousands of nodes).
For this study, we could simply use a strong cloud instance with 1-2TB of RAM and dozens of virtual CPUs.

While traditional scientometric datasets, such as Scopus, Mendeley, Medline, PsychINFO, etc., are useful for scientometric research, they are usually limited to specific domains and time.
This limits the ability to observe global trends, such as those presented throughout this study. Moreover, the MAG dataset has additional benefits, such as author disambiguation and mapping papers to topics.
In fact, the use of the MAG dataset for scientometrics has gained increasing popularity in recent years.
Moreover, a recent study by Herrmannova and Knoth [1] describes in detail the properties of the MAG dataset,
including various statistics like the number of papers in each field of study and the comparison of the

dataset to other scientometric datasets, such Mendeley.
For example, according to Herrmannova and Knoth’s study, the MAG dataset contains nearly 15 million papers in the field of biology, while the Mendeley dataset contains fewer than 300,000 biological science papers [1].

While MAG is a great tool for scientometric research, the MAG dataset didn’t contain all the paper features we required for this research.
Therefore, we utilized the AMiner dataset to add additional features and to compare results with those obtained using the MAG dataset in order to validate the existence of observed patterns in both datasets.
The AMiner is indeed a relatively new dataset, and we are among the first to use it for a scientometric study.

In the revised manuscript, we have elaborated on the MAG dataset and its increasing popularity. We also have added a reference to Herrmannova and Knoth’s paper [1].

Comment 8: Authors should consider to better explain how Q1, Q2, Q3 and Q4 are defined in the SCImago journal rank dataset.

Response 8: In the revised manuscript, we have elaborated on the quartile definition and use.

Comment 9: Authors should also better describe the L0 to L3 classification: on which value is based the hierarchy ranking?

Response 9: In the revised manuscript, we have further developed our explanation of the field-of-study classifications.
Additionally, we have added a reference to the Herrmannova and Knoth study [1], which contains an in-depth analysis of the various fields of study in the MAG dataset.

- Analyses

Comment 10: Authors should better explain how they deal with non-English papers (since a specific analysis on languages appears in the beginning of the Results section).

Response 10: We thank the reviewer for this comment. In order to detect paper language, we utilized the pycld2 python package, which can identify a text language.
The main advantage of using pycld2 is its speed, which is critical for analyzing over 100 million titles and abstracts, and we also appreciate its ease of use (one line of code).

In the revised manuscript, we elaborate on how we use pycld2 for language detection. Moreover, in the code section of the project’s website there are more details on the creation of each result, including identifying non-English papers and presenting additional results regarding publication trends of non-English papers.

Comment 11: Y axis of Fig 5 should be labelled.

Response 11: We have added a Y-axis label to Figure 5.

Comment 12: The analysis of the total number of papers with no citations (Fig 9) should be presented using proportion data (%), so Fig S11 should be preferred to Fig 9 in the main text (+ there is a typo in the title of Fig 9 "aftetr"). Presenting a crude increase is not very useful, given the overall growth of yearly number of publications.

Response 12: As recommended, in the revised manuscript, we have swapped Figure S11 with Figure 9 and fixed the typo.

- Results of Author Trends

Comment 13: A global information on how many unique author ID have been identified would be important. Footnote number 10 should be quantified: what is the proportion of unique authors with several IDs?

Response 13: We have mentioned the number of unique author IDs in the Data Description section (22.4 million authors with a unique author ID).
Unfortunately, there are no available data for the performance of the author disambiguation algorithm used in the MAG dataset. Therefore, we aren’t able to add an estimation of the number of authors with several IDs.

- Results of Journal Trends

Comment 14: The authors should avoid to give information about methods in the result section : "We matched the journals' titles and ISSNs ..." and subsequent sentences would be better in the Methods section.

Response 14: We agree with the reviewer, and these sentences are now located more appropriately in the Analyses section.

Comment 15: Y-axis of Figure 8 should be labelled more precisely (number of pages?)

Response 15: We appreciate the reviewer’s close attention to detail. We have updated the figure’s Y-axis to be “Papers’ Average Number of Pages.“

References

[1] Herrmannova, Drahomira, and Petr Knoth. "An analysis of the Microsoft academic graph." D-Lib Magazine 22.9/10 (2016)

Close
