# Supplementary material for: Over-optimization of academic publishing metrics: observing Goodhart’s Law in action
Source: Gigascience. 2019 May 30;8(6):giz053. doi: 10.1093/gigascience/giz053 (PMC6541803; doi:10.1093/gigascience/giz053)
Supplement: giz053_Response_to_Reviewer_Comments_Revision_2 [file giz053_response_to_reviewer_comments_revision_2.pdf]

Author's Response To Reviewer Comments

Close

-

Close
