# Supplementary material for: Over-optimization of academic publishing metrics: observing Goodhart’s Law in action
Source: Gigascience. 2019 May 30;8(6):giz053. doi: 10.1093/gigascience/giz053 (PMC6541803; doi:10.1093/gigascience/giz053)
Supplement: giz053_Reviewer_1_Report_Original_Submission -- Jean-Noel Vergnes — 12/7/2018 Reviewed [file giz053_reviewer_1_report_original_submission.pdf]

Reviewer Report

Title: Over-Optimization of Academic Publishing Metrics: Observing Goodhart's Law in Action

Version: Original Submission **Date:** 12/7/2018

Reviewer name: Jean-Noel Vergnes

Reviewer Comments to Author:

This article is interesting and provides important information on the evolution of academic publishing across different fields of research.
Here are some comments that could help readers to better appreciate the paper:
1) Introduction.
Figure 1 is very interesting, however, it appeared far too early in the paper. It makes the figure not very understandable (readers at this stage have no idea on how these data have been collected and analyzed). A citation (or several citations if needed) from the literature would be sufficient to describe the exponential growth of academic publishing.
Introduction shouldn't reports results from the present study (so Figures 14 and S17 shouldn't be mentioned in this section) - numbering of figures should also be checked in the entire article (Figure 14 shouldn't follow the Figure 1 in the order of apparition).
Maybe Figure 1 should be provided as a summary-of-results, later in the paper (in the discussion section?)
Again in the Introduction section, paragraphs Papers, Authors, Journals and Fields of Research are in fact summary of results. It should be reported later in the paper.
The introduction section should present hypotheses that were formulated before analyses were performed.
Paragraphs "These observations support the hypothesis []... (see the Results of Paper Trends section and Figure S13)" and
"It is time to consider [...] academic publishing world" are in fact discussion paragraphs.
There is a need in the introduction section to formulate the general objective of the paper.
2) Background
The sentence "In this section, we give a short overview of the relevant scientometric papers to this study" is unclear. Suggestion: "In this section, we present studies that analyze changes in academic publications in recent years ..."
Paragraph " Our study is greatly influenced by a recent study by [...](and hence the status) of the research." would be better in the introduction section.
3) Data description.
Clearly written.
DOI is a good way of identifying an article, but the "unique author ID value" is not very clearly explained. However, it is quite uncommon to use these datasets for scientometric purposes (if not, please provide examples of such previous use).
Authors should better explain why they use these datasets instead of more traditional databases (e.g,

for biomedical research, scopus, embase, medline, psychinfo etc). They should also explain how fields of research are integrated into these datasets, how complete they are, how representative of the literature they are.
Main comment of this reviewing: More precisions on datasets that have been used are very important to assess external validity of the present analyses (are the references included in these datasets representative of the overall knowledge?).
Authors should consider to better explain how Q1, Q2, Q3 and Q4 are defined in the SCImago journal rank dataset.
Authors should also better describe the L0 to L3 classification: on which value is based the hierarchy ranking?
4) Analyses
Authors should better explain how they deal with non-English papers (since a specific analysis on languages appears in the beginning of the Results section).
Y axis of Fig 5 should be labelled.
The analysis of the total number of papers with no citations (Fig 9) should be presented using proportion data (%), so Fig S11 should be preferred to Fig 9 in the main text (+ there is a typo in the title of Fig 9 "aftetr"). Presenting a crude increase is not very useful, given the overall growth of yearly number of publications.
Results of authors trends
A global information on how many unique author ID have been identified would be important. Footnote number 10 should be quantified: what is the proportion of unique authors with several IDs?
Results of Journal trends
The authors should avoid to give information about methods in the result section : "We matched the journals' titles and ISSNs ..." and subsequent sentences would be better in the Methods section.
Y-axis of Figure 8 should be labelled more precisely (number of pages?)
Discussion
Very well written, understandable and very interesting.

Methods

Are the methods appropriate to the aims of the study, are they well described, and are necessary controls included? Choose an item.

Conclusions

Are the conclusions adequately supported by the data shown? Choose an item.

Reporting Standards

Does the manuscript adhere to the journal's guidelines on minimum standards of reporting? Choose an item.

Choose an item.

Statistics

Are you able to assess all statistics in the manuscript, including the appropriateness of statistical tests used? Choose an item.

Quality of Written English

Please indicate the quality of language in the manuscript: Choose an item.

Declaration of Competing Interests

Please complete a declaration of competing interests, considering the following questions:

- Have you in the past five years received reimbursements, fees, funding, or salary from an organisation that may in any way gain or lose financially from the publication of this manuscript, either now or in the future?
- Do you hold any stocks or shares in an organisation that may in any way gain or lose financially from the publication of this manuscript, either now or in the future?
- Do you hold or are you currently applying for any patents relating to the content of the manuscript?
- Have you received reimbursements, fees, funding, or salary from an organization that holds or has applied for patents relating to the content of the manuscript?
- Do you have any other financial competing interests?
- Do you have any non-financial competing interests in relation to this paper?

If you can answer no to all of the above, write 'I declare that I have no competing interests' below. If your reply is yes to any, please give details below.

No

I agree to the open peer review policy of the journal. I understand that my name will be included on my report to the authors and, if the manuscript is accepted for publication, my named report including any attachments I upload will be posted on the website along with the authors' responses. I agree for my report to be made available under an Open Access Creative Commons CC-BY license (http://creativecommons.org/licenses/by/4.0/). I understand that any comments which I do not wish to be included in my named report can be included as confidential comments to the editors, which will not be published.

I agree to the open peer review policy of the journal

To further support our reviewers, we have joined with Publons, where you can gain additional credit to further highlight your hard work (see: https://publons.com/journal/530/gigascience). On publication of this paper, your review will be automatically added to Publons, you can then choose whether or not to claim your Publons credit. I understand this statement.

Yes Choose an item.
